# Microbiome eco-evolution of cultivated and wild rice species across the genus *Oryza* and its importance in supporting rice growth

**DOI:** 10.1186/s40168-026-02359-z

**Published:** 2026-03-06

**Authors:** Fei Luo, Yicong Cai, Yujie Cui, Xiangyang He, Jiawang Xu, Wanqiu Tang, Xiaoqing Wang, Yaohui Cai, Hongwei Xie, Wei Chen, Wenzhuo Li, Xia Ding

**Affiliations:** 1 School of Life Sciences, Jiangxi Province Key Laboratory of Drug Target Discovery and Validation, Nanchang University, Nanchang, China; 2 Institute of Biomedical Innovation, Jiangxi Medical College, Nanchang University, Nanchang, China; 3 Ministry of Education Key Laboratory of Crop Physiology, Ecology and Genetic Breeding, Jiangxi Agricultural University, Nanchang, China; 4 Jiangxi Super-Rice Research and Development Center, Jiangxi Academy of Agricultural Sciences, Nanchang, China

**Keywords:** Crop microbiome, Wild rice, Eco-evolution, Core microbiome, SynCom

## Abstract

**Background:**

Crop wild relatives and their microbiomes are essential for sustainable crop production. However, the co-evolution of wild rice species and their microbiomes remains poorly understood. Herein, we investigated microbiome assembly across 17 wild rice and one cultivated rice species under controlled conditions spanning ~15 million years of evolution.

**Results:**

Our data reveal distinct eco-evolutionary patterns for bacteria and fungi. Host divergence time was the predominant driver of root microbiota structure, outweighing polyploidy and life cycle, and exerted a stronger effect on bacteria than fungi. Bacterial community exhibited a significant phylosymbiosis with its host, but fungi did not. Over evolutionary time, bacterial diversity decreased while phylogenetic clustering increased. Deterministic and stochastic processes co-drove bacteria assembly, whereas stochastic processes strongly drove fungi assembly. Potentially functional taxa, including nitrogen-fixing and methane-cycle bacteria, were differentially enriched across evolutionary time and polyploidization events. Notably, co-speciating bacteria better predicted grain weight than fungi, with core species making a major contribution. Using a synthetic community (SynCom) derived from the wild rice core microbiome and four nitrogen-fixing strains enriched in early- and medium-diverging *Oryza* species, we demonstrated that the SynCom strongly promoted rice growth, with the removal of key members markedly reducing its impact.

**Conclusions:**

These results reveal co-phylogenetic patterns between *Oryza* and root-associated bacteria, highlighting the closer functional linkage between rice traits and bacteria than fungi, likely due to their co-evolution. Our findings provide new insights into crop–microbiome symbiosis from an eco-evolutionary perspective and underscore the importance of co-speciating microbiomes from wild relatives in supporting crop growth.

Video Abstract

**Supplementary Information:**

The online version contains supplementary material available at 10.1186/s40168-026-02359-z.

## Introduction

Rice (*Oryza sativa* L.) is a staple food for more than half of the world’s population. The genus *Oryza* is a model system for the study of evolution over timescales ranging from a few thousand to 15 million years. The genus *Oryza* consists of 25 wild rice species and 2 cultivated rice species (*Oryza sativa* and *Oryza glaberrima*) [1–3]. *O. sativa* is the most widely grown of the two cultivated species. These wild rice are currently divided into 5 complexes (Sativa, Officinalis, Meyeriana, Ridleyi, Unclassified), 11 genome types (AA, BB, CC, EE, FF, GG, BBCC, CCDD, HHJJ, HHKK, and KKLL), and 2 chromosome types (diploid and allotetraploid), making the genus an excellent model system for testing how host genotypes influence the microbiome and are invaluable resources for understanding host and microbiome co-evolution mechanisms [3–7]. Wild rice contains many valuable traits, including high biomass on poor soils, high nitrogen use efficiency, and resistance to pests and diseases. Humans domesticated wild rice into cultivated rice, and its agronomic traits, such as plant height and yield, changed significantly. However, the genetic diversity of cultivated rice is narrower than ever before, and many excellent traits have been lost [8]. Wild species serve as valuable reservoirs of genetic diversity and desirable agronomic traits that can be used to improve rice [9–11]. There are many successful cases of using wild relatives of the genus *Oryza* to introgress favorable traits into cultivated rice [12, 13].

Crop wild relatives and their microbiome are essential to unlock novel mechanisms for crop resilience and new strategies for achieving food security [14]. The crop microbiome influences plant growth and health, and is considered one of the most promising long-term solutions to achieve global food security [15, 16]. However, how the microbiome is structured during *Oryza* speciation and whether important functional microbiota is lost remain elusive. We know little about whether cultivated rice has also lost many excellent functional microorganisms during evolution. Importantly, little research has been conducted on how to apply the wild rice microbiota to rice cultivation. Therefore, there is an urgent need to fully understand the structure and function of the wild rice microbiome and its interactions with host species to develop effective microbiome-based technologies for sustainable agriculture.

In agriculture, we need to determine whether the host-associated microbiome has shifted due to crop speciation, including domestication and polyploidization, and whether potentially beneficial microbial symbionts have been lost or acquired during crop evolution. Several studies have demonstrated that the domestication of plants has resulted in many changes in the microbiome and their effects on agroecosystems [2, 17–19]. However, only a few studies have explored the effects of polyploidization on biotic interactions. Polyploidy, or whole-genome duplication (WGD), is common in crops. Many important crop species are polyploids, such as cotton, wheat, rapeseed, and tobacco [20–22]. There is evidence indicating that polyploidization might increase mutualistic associations, such as legume–rhizobium interactions [23]. Therefore, understanding the microbiome response to crop evolution, including polyploidization and domestication, is not only crucial for illustrating the natural history of crop–microbiome interactions in general but would also facilitate crop domestication and crop improvements.

A key question regarding the host–microbiome interaction is the extent to which the beneficial microbes have coevolved with the host. Phylosymbiosis has recently been proposed to describe the host–microbiome eco-evolutionary pattern, whereby host-associated microbial community composition parallels host phylogenetic relationships [24–26]. Phylosymbiosis has been reported for many host-microbial symbiosis systems, such as the marine sponge microbiome, coral microbiome, root microbiome of flowering plants, ant microbiome, bat microbiome, ape microbiome, the gut microbiome of terrestrial mammals, and mammalian skin microbiome [24, 27, 28]. Significant degrees of phylosymbiosis are prevalent in microbiomes of aquatic and terrestrial plants and animals, but are not universal [29]. No strict patterns of phylosymbiosis have been observed in termites, flies [24, 30], birds [31–34], and western chipmunks [35]. Although host–microbiota interactions are frequently characterized as cooperative or mutualistic, the evolutionary processes that generate and maintain such cooperation remain unclear. The holobiont or hologenome concept proposes that hosts and their microbiota can act as a single evolutionary unit, but this view has been challenged, particularly for systems with highly diverse and environmentally acquired microbiomes. Evolutionary conflicts may arise both between the host and its microbiota and among microbial taxa themselves. Recent evolutionary modeling has shown that stable cooperation is difficult to maintain in microbiomes with many strains and rapid turnover, unless hosts evolve mechanisms of control that selectively favor beneficial symbionts [36, 37]. Such host-control mechanisms—including immune filtering and selective nutrient provisioning—can in turn promote cooperative evolution when microbial partners cannot easily evade host regulation [38]. Lewin-Epstein and Hadany reported that host–microbiome co-evolution could promote cooperation in rock-paper-scissors dynamics [39]. The accumulated evidence showed that microbiotas could affect the host’s health and behavior, but hosts could evolve mechanisms to resist microbial manipulation. At present, host control mechanisms suggest that cooperative evolution can occur when host control is effective [36].

**Fig. 1:**
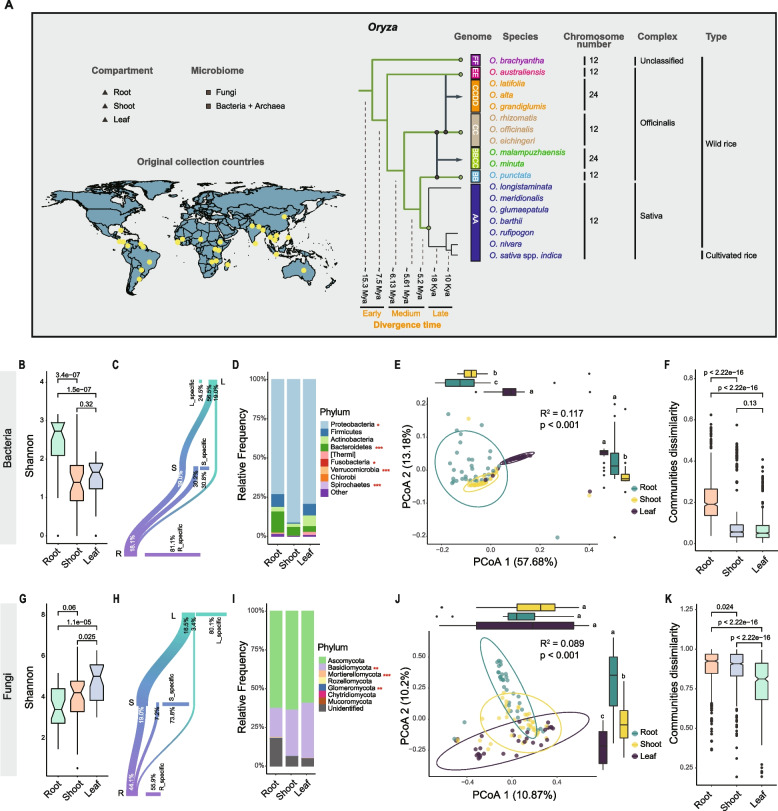
Overview of host diversity and microbiome structure across the *Oryza* genus. **A** Overview of the evolutionary and geographic diversity of the *Oryza* accessions sampled in this study. Information on the 89 *Oryza* accessions. The compartment, chromosome number, complex, genome, and species of the genus *Oryza* and their original collection countries of the genus *Oryza*. The diagram indicated the diverse worldwide distribution origin of wild and cultivated *Oryza*. Phylogenetic tree of the genus *Oryza* (modified from REFS [3, 4, 7]). Closed circles represented the diploids, and arrows indicated the origin of the polyploids. Mya, million years ago. Kya, thousand years ago. Tip colors in the phylogenetic tree indicate the genome types of each *Oryza* species. Yellow circles on the world map indicate the original geographic collection sites of the seeds for the *Oryza* accessions used in this study. **B**–**K** Overview of bacterial and fungal community structure across root, shoot, and leaf compartments across the *Oryza* genus. Alpha diversity (Shannon indices) of the root, shoot, and leaf bacteria (**B**) and fungi (**G**). *p* values reflected the Wilcoxon test. Numbers of different ASVs of bacteria (**C**) and fungi (**H**) communities among each compartment in the Sankey diagram. R: root, S: shoot, L: leaf. R_specific: ASVs unique to the root compartment, S_specific: ASVs unique to the shoot compartment, L_specific: ASVs unique to the leaf compartment. The relative abundance of bacterial (**D**) and fungal (**I**) phyla was significantly affected by compartments (red asterisk denotes significance: GLM, *p < 0.05, **p < 0.01, ***p < 0.001). PCoAs of bacterial (**E**) and fungal (**G**) community composition among different compartments based on Bray–Curtis distance. Boxplots (top and right panels) indicate the distribution of samples from each compartment along the first (PC1) and second (PC2) principal coordinates, respectively. Significant differences (Tukey’s HSD, *p* < 0.05) were shown by different letters. **F**, **K** Within-compartment heterogeneity of microbial communities. Boxplots show the distribution of pairwise Bray–Curtis dissimilarities among samples from the same compartment. Lower values indicate more homogeneous communities within that tissue. Significant differences among compartments were assessed using the Wilcoxon test (*p* < 0.001)

Here, we investigated how natural variation among 18 *Oryza* species, spanning ~15 million years of evolution and grown under controlled conditions in a natural paddy field, shapes microbiome assembly (Fig. 1A; Table S1). This panel included one cultivated rice (*O. sativa* ssp. *indica*) and 17 wild species, representing two chromosome numbers (2n = 24 or 48), three complexes (Sativa, Officinalis, and unclassified), and seven genome types (AA, BB, BBCC, CC, CCDD, EE, and FF). Based on published divergence time estimates [3–5, 7, 8, 40–42], *Oryza* evolution was categorized into three broad phases: an early phase (~15.3–7.5 Mya) comprising EE- and FF-genome species, a medium phase (~6.13–5.2 Mya), and a late phase (~18–10 Kya) encompassing recently diverged AA-genome species (Fig. 1A). An initial overview of microbial community structure across root, shoot, and leaf compartments was conducted to establish general compartment-level patterns within the *Oryza* genus (Fig. 1B–K). Building on this framework, we subsequently focused our analyses on the root compartment, where host–microbiome interactions are most directly linked to nutrient acquisition and plant performance. Within this root-centered context, we addressed three interconnected questions: (1) How does host evolutionary divergence structure root-associated microbial communities, and are evolutionarily conserved or functionally important microbial taxa lost or retained over time? (2) Do host phylogenetic relationships correspond to microbial community relationships, consistent with phylosymbiosis? (3) Can conserved root microbial assemblages or key functional taxa from wild rice be reconstructed to enhance the growth of cultivated rice? By integrating community-level diversity analyses, cophylogenetic comparisons, functional inference, and experimental validation using synthetic communities, this study links microbiome eco-evolutionary patterns with agronomic relevance.

## Results

### Compartmental organization of bacterial and fungal communities across the *Oryza* genus

All *Oryza* accessions, despite their diverse geographic origins (Fig. 1A), were cultivated under a common-garden paddy field to control for environmental variation. To elucidate the microbial community structure of *Oryza* species, we analyzed the fungal and bacterial taxonomic structures and patterns in the roots, shoots, and leaves of samples grown under the same paddy field with the same cultivation practices during the 2015 growing season (Fig. 1A, Table S1). Across the compartments, the bacteria exhibited higher alpha diversity in the roots than in the leaves and shoots, while the fungi exhibited higher alpha diversity in the leaves than in the roots and shoots (*p* < 0.001, Wilcoxon test) (Fig. 1B, G, S1). Analysis of the Sankey diagrams, now augmented with percentage values, revealed clear compartmentalization patterns. For bacteria, most leaf (75.5%) and shoot (69.2%) ASVs were also present in roots, and the root compartment harbored the highest proportion of unique ASVs (81.1%). This indicates a nested structure in which aboveground bacterial communities are largely derived from the root reservoir. In contrast, fungal communities displayed strong compartment specificity, with leaves (80.1%), shoots (73.8%), and roots (55.9%) each containing substantial proportions of unique ASVs (Fig. 1C, H, S2, Table S2).

Taxonomic classification revealed that the dominant bacterial phyla were Proteobacteria, Firmicutes, Actinobacteria, and Bacteroidetes (Fig. 1D). At the phylum level, Proteobacteria, Firmicutes, Actinobacteria, Bacteroidetes, and Verrucomicrobia exhibited significantly different relative abundances across the compartments (*p* < 0.05, GLM) (Fig. 1D, Table S3). At the genus level, 21% of the bacterial genera exhibited significantly different relative abundances across compartments, such as *Xanthomonas*, *Halomonas*, *Pseudomonas*, *Stenotrophomonas*, *Herbaspirillum*, and *Shewanella* (*p* < 0.05, GLM) (Fig. S3, Table S3). The dominant fungal phyla were Ascomycota, Basidiomycota, and Mortierellomycota (Fig. 1I). At the phylum level, Mortierellomycota and Glomeromycota exhibited significantly different relative abundances across the compartments (*p* < 0.05, GLM) (Fig. 1I, Table S4). At the genus level, 13.2% of the fungal genera exhibited significantly different relative abundances across compartments, such as *Gibberella*, *Apodus*, *Fusarium*, *Sarocladium*, *Lactifluus*, *Meyerozyma*, and *Hannaella* (*p* < 0.05, GLM) (Fig. S3, Table S4).

We found clear differences in the microbial community composition of the root, shoot, and leaf compartments (*p* < 0.001, PERMANOVA) (Fig. 1E, J), and all pairwise comparisons between compartments (root vs. shoot, root vs. leaf, shoot vs. leaf) were statistically significant (*p* < 0.05, pairwise PERMANOVA) (Table S5). The Bray–Curtis dissimilarity showed that the bacterial communities in roots exhibited a greater dissimilarity than those in shoots and leaves, and the fungal communities in roots and shoots exhibited a greater dissimilarity than those in leaves (*p* < 0.001, Wilcoxon test) (Fig. 1F, K).

In summary, plant compartments exert strong structuring effects on the endosphere microbiota of wild rice. Notably, wild rice exhibits clear kingdom-specific assembly patterns: bacterial communities largely follow a root-reservoir model, whereas fungal communities show pronounced compartment specificity, reflecting functionally distinct niches of kingdom-specific microbiomes across wild rice tissues. Given our focus on host genetic and eco-evolutionary effects on nutrition-related microbiota, the root—serving as the primary interface for nutrient uptake and belowground interactions—provides the ideal compartment for investigating host-driven microbiome assembly. To determine whether host–root microbiome associations were robust to environmental variability, we conducted a cross-year analysis using the subset of *Oryza* accessions sampled in both 2015 and 2019. This analysis revealed strong inter-annual effects; however, even in the presence of pronounced year-to-year variation, a host genetic signal remained detectable (Fig. S4). Given the confounding influence of inter-annual variation, we proceeded with the larger, environmentally uniform 2019 dataset to maximize statistical power for detecting subtler host evolutionary patterns. With this root-associated ecological niche and temporal baseline established, we next investigated how host evolutionary divergence shapes the root microbiome.

### Host evolutionary divergence structures root-associated microbial communities

**Fig. 2:**
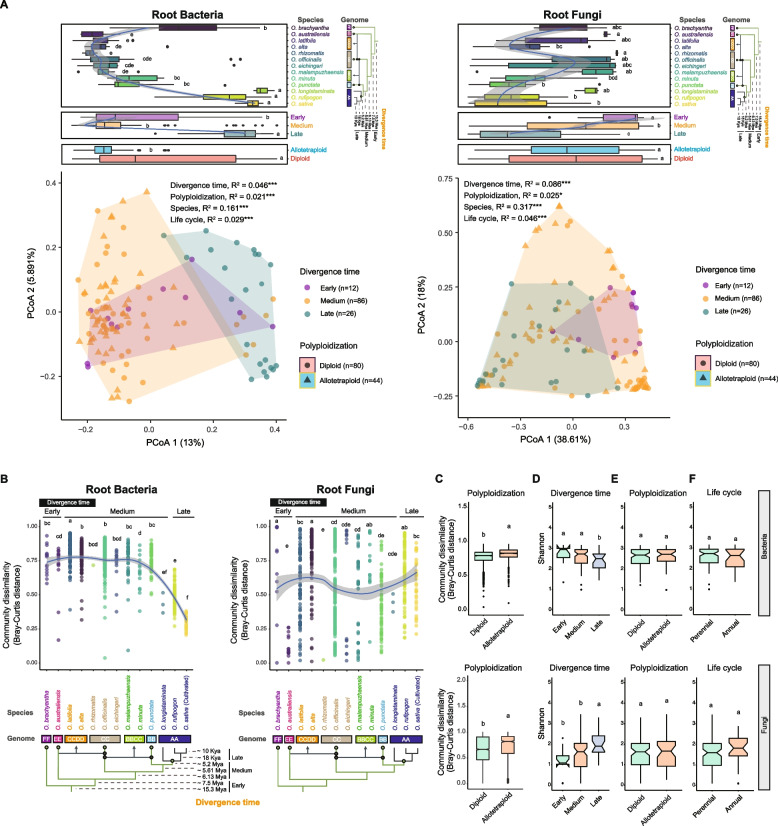
Root microbial community alpha and beta diversity across host species. **A** Principal coordinate analysis (PCoA) of root bacterial and fungal communities based on the Bray–Curtis distance matrix, using root samples collected from the 2019 common-garden field experiment. Boxplots (top panel) indicate the distribution of each host factor group along the first principal coordinate (PCoA1). Species labels in the phylogenetic tree are color-coded to match the corresponding host groups in the boxplots, enabling direct visual linkage between host evolutionary relationships and microbiome variation. PERMANOVA showed significant differences in community composition (*p < 0.05, **p < 0.01, ***p < 0.001). **B** The Bray–Curtis dissimilarity of the root bacterial and fungal communities. Temporal dynamics of the Bray–Curtis dissimilarity of the root bacterial and fungal communities among divergence times within the genus *Oryza*. Loess trend curves were shown. **C** The Bray–Curtis dissimilarity of the root bacterial and fungal communities was colored by polyploidy types. **D** Shannon of root bacterial and fungal communities was colored by divergence time. **E** Shannon of root bacterial and fungal communities colored by polyploidy types. **F** Shannon diversity of root bacterial and fungal communities colored by life cycle types. Mya, million years ago. Kya, thousand years ago. Significant differences (*p* < 0.05, Wilcoxon test) were shown by different letters

To reveal how host genetic factors shaped the root microbiome across the genus *Oryza*, we next examined the impact of host speciation on the root microbial taxonomic composition of samples grown under the same paddy field with the same cultivation practices during the 2019 growing season (Table S1). Taxonomic classification of the root bacterial sequences at the phylum level revealed the prevalence of Proteobacteria as the dominant phylum (81.32%), followed by Bacteroidetes (6.01%), Firmicutes (5.58%), and Actinobacteria (2.26%) (Fig. S5, Table S6). The root fungal reads were dominated by the phyla Basidiomycota (82.93%) and Ascomycota (16.40%) (Fig. S6, Table S7). Principal coordinate analysis (PCoA) revealed significant differences in root bacterial (*p* < 0.002) and fungal (*p* < 0.05) community composition associated with host divergence time, species, polyploidy, and life cycle. For bacteria, host divergence time, species, polyploidy, and life cycle explained 4.6% (*R*^2^ = 0.046, *p* = 0.001), 16.1% (*R*^2^ = 0.161, *p* = 0.001), 2.1% (*R*^2^ = 0.021, *p* = 0.001), and 2.9% (*R*^2^ = 0.029, *p* = 0.001) of the total community variability, respectively. For fungi, the same factors explained 8.6% (*R*^2^ = 0.086, *p* = 0.001), 31.7% (*R*^2^ = 0.317, *p* = 0.001), 2.5% (*R*^2^ = 0.025, *p* = 0.012), and 4.6% (*R*^2^ = 0.046, *p* = 0.001) of the variability, respectively (Fig. 2A, S7, Table S8).

To further explore the strong species effect, we examined root microbiome structure across taxonomic complexes and domestication status. At the complex level, root communities differed significantly between the Officinalis complex (non-AA genomes) and the Sativa complex (AA genomes) (bacteria: *R*^2^ = 10.3%, *p* = 0.001; fungi: *R*^2^ = 4.5%, *p* = 0.002) (Fig. S8). Strikingly, within the AA-genome Sativa complex, the impact of domestication and breeding was even more pronounced: cultivated *O. sativa* ssp. *indica* harbored root microbiomes that were highly distinct from those of their wild AA-genome relatives (bacteria: *R*^2^ = 25.7%, *p* = 0.001; fungi: *R*^2^ = 9.0%, *p* = 0.046) (Fig. S8). Notably, this domestication effect alone explained a larger fraction of bacterial community variation than the deep evolutionary divergence across the genus, emphasizing the rapid and powerful influence of human selection. Together, these results revealed a hierarchical pattern in which ancient evolutionary divergence and recent domestication act as two sequential filters shaping the composition of the rice root microbiome.

While ecological differences among host species can influence microbiome composition, evolutionary history has had a strong effect on the root bacterial communities. Quantitative analysis of community dissimilarity showed that Bray–Curtis dissimilarity of the root bacterial community significantly declined within the late-diverged *Oryza* species (*O. longistaminata*, *O. rufipogon*, and *O. sativa*) (*p* < 0.05, Wilcoxon test), with the cultivated species (*O. sativa*) exhibiting the lowest dissimilarity (Fig. 2B, S9). This pattern indicates a progressive homogenization and host-specific specialization of the root bacterial assemblage. In stark contrast, the root fungal community did not display this pattern of increasing similarity (*p* > 0.05, Wilcoxon test). Additionally, polyploidization significantly increased the Bray–Curtis dissimilarity of root bacteria and fungi (*p* < 0.05, Wilcoxon test) (Fig. 2C).

Analysis of alpha diversity revealed consistent trends across diversity metrics but distinct patterns for bacteria and fungi over evolutionary time. Bacterial Shannon was significantly decreased within the late-diverged *Oryza* species (*p* < 0.05, Wilcoxon test; Fig. 2D), a pattern corroborated by the Faith’s PD index (Fig. S10). Conversely, fungal Shannon significantly decreased within the early-diverged *Oryza* species (*p* < 0.05, Wilcoxon test; Fig. 2D), with the Faith’s PD showing a similar trend (Fig. S10). In addition, polyploidy and life cycle had no significant effect on the bacterial and fungal Shannon and Faith’s PD (*p* > 0.05, Wilcoxon test) (Fig. 2E, F, S10). Together with PCoA, host divergence time remains the strongest predictor of microbiome structure and function, while polyploidy and life cycle traits have only minor and comparable impacts.

Cophylogeny of the root microbiome and *Oryza*

**Fig. 3:**
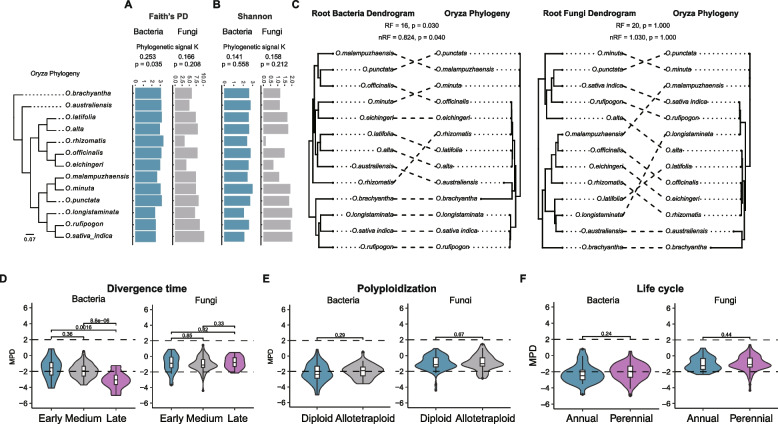
Estimating the phylosymbiosis signal and the phylogenetic structure of root microbial communities. **A**, **B** The effect of host plant species on the microbial Faith’s PD and Shannon. Significant phylogenetic signals are assessed using Blomberg’s *K* statistics and *p* values. **C** Phylosymbiosis between host phylogeny and microbial community dendrogram relationships. **D** Variation in the MPD of microbial communities during *Oryza* evolution. Horizontal dashed lines indicate the upper (+2) and lower (−2) significance thresholds. **E** Variation in MPD of microbial communities under polyploidization. **F** Variation in the MPD of microbial communities under life cycle

To test whether phylosymbiosis has occurred during the eco-evolution of *Oryza*–microbiome associations, we tested whether closely related *Oryza* species shared a similar microbiome. The bacterial Faith’s PD diversity index (Blomberg’s *K* = 0.253, *p* = 0.035) exhibited a significant phylogenetic signal, but fungi did not (Fig. 3A, B).

Comparing host plant phylogeny to dendrograms of corresponding root microbial communities (constructed using hierarchical clustering of Bray–Curtis distances) for each *Oryza* species indicated that relationships among bacteria were more similar to their host evolutionary relationships than those of fungi (Fig. 3C). The bacterial dendrograms and host phylogenies exhibited significant co-phylogeny (Robinson–Foulds (RF) = 16, *p* = 0.030), while the fungal dendrograms and host phylogenies did not (RF = 20, *p* = 1.000). Next, the bacteria–host holobiont possessed a normalized Robinson–Foulds distance (nRF) score of 0.824 and demonstrated a weak but significant topological congruence with the *Oryza* phylogeny and bacterial dendrogram (nRF = 0.824, *p* = 0.040). However, the fungi–host holobiont demonstrated little congruence, as indicated by the nRF score of 1.030, and no significant congruence with the *Oryza* phylogeny and fungal dendrogram (nRF = 1.030, *p* = 1.000). The significance of this topological congruence was further confirmed using the Clustering Information Distance (CID), a metric sensitive to shared evolutionary partitions. The host–bacteria association was significant (CID = 9.75, *p* = 0.039), while the host–fungi association was not (CID = 11.03, *p* = 0.142), validating the phylosymbiosis signal specific to bacteria. These results indicated that the bacteria–host holobiont exhibited significant phylosymbiosis, but that of the fungi–host holobiont did not.

Next, we examined how *Oryza* speciation processes (divergence time, polyploidy, and life cycle) shaped the phylogenetic structure of root-associated microbial communities. For bacteria, phylogenetic MPD (standardized effect sizes) was significantly lower in late-diverged *Oryza* species than in early- or medium-diverged species (*p* < 0.05, Wilcoxon test; MPD < −2), indicating stronger phylogenetic clustering (Fig. 3D). In contrast, fungal MPD showed no significant differences among early, medium, and late divergence categories. To provide a complementary, tree-independent perspective, we computed a *k*-mer–based MPD-like metric. This analysis reproduced the key trends: bacterial *k*-mer MPD-like metric was significantly lower in late-diverged species, whereas fungal *k*-mer MPD-like metric showed no divergence-time effect (Fig. S11). These consistent results reinforce that host divergence time is the primary determinant of bacterial community structure. Neither polyploidy nor life cycle significantly affected bacterial or fungal phylogenetic MPD (*p* > 0.05, Wilcoxon test; Fig. 3E, F), and the *k*-mer–based results corroborated this absence of effect (Fig. S11). Together, these findings indicate that host divergence time exerted a strong impact on shaping the root microbiome assembly compared to polyploidy and life cycle, with late-diverged *Oryza* species exhibiting pronounced bacterial phylogenetic clustering, while polyploidy and life cycle exert minimal influence.

### Deterministic and stochastic processes in root microbiome assembly

Null model analysis and neutral community model (NCM) analysis showed that stochasticity processes contributed mainly to the fungal community, while stochasticity and deterministic processes co-structured the bacterial community (Figs. S12 and S13). For the bacterial assembly, the relative contributions of stochasticity and determinism were 64.1% and 35.9%, respectively (Fig. S13). The effects of deterministic processes dramatically increased from early- to late-diverged *Oryza* species. For the fungal assembly, the relative contributions of stochasticity and determinism were 95.9% and 4.1%, respectively. Moreover, polyploidy did not significantly affect the bacterial and fungal assembly processes. However, life cycle significantly affected the bacterial assembly processes, but not the fungi community. The relative contributions of determinism processes were increased in the perennial bacterial community.

In summary, our findings underscore the dominance of host divergence time in shaping microbiome structure and function, while polyploidy and life cycle traits exert only minor, comparable influences. These results highlight the importance of deep evolutionary history over more recent life-history transitions in the eco-evolutionary relationship between *Oryza* and its root microbiota.

### Potentially functional root microbial taxa responsive to *Oryza* speciation

Next, we examined potentially functional root microbial taxa involved in nitrogen fixation and methane metabolism that are retained or lost during *Oryza* evolution, due to their central roles in the carbon and nitrogen cycles of rice paddies. Nitrogen-fixing bacteria provide bioavailable nitrogen crucial for rice growth, while methane-cycling microbes (methanogens and methanotrophs) influence greenhouse-gas fluxes and, in some cases, also fix nitrogen, making them dual-function taxa of ecological and agronomic relevance. We then assessed how host evolutionary divergence—including divergence time, ploidy, genome type, reproductive complex type, and species identity—shapes the assembly of these microbial groups across the *Oryza* lineage.

**Fig. 4:**
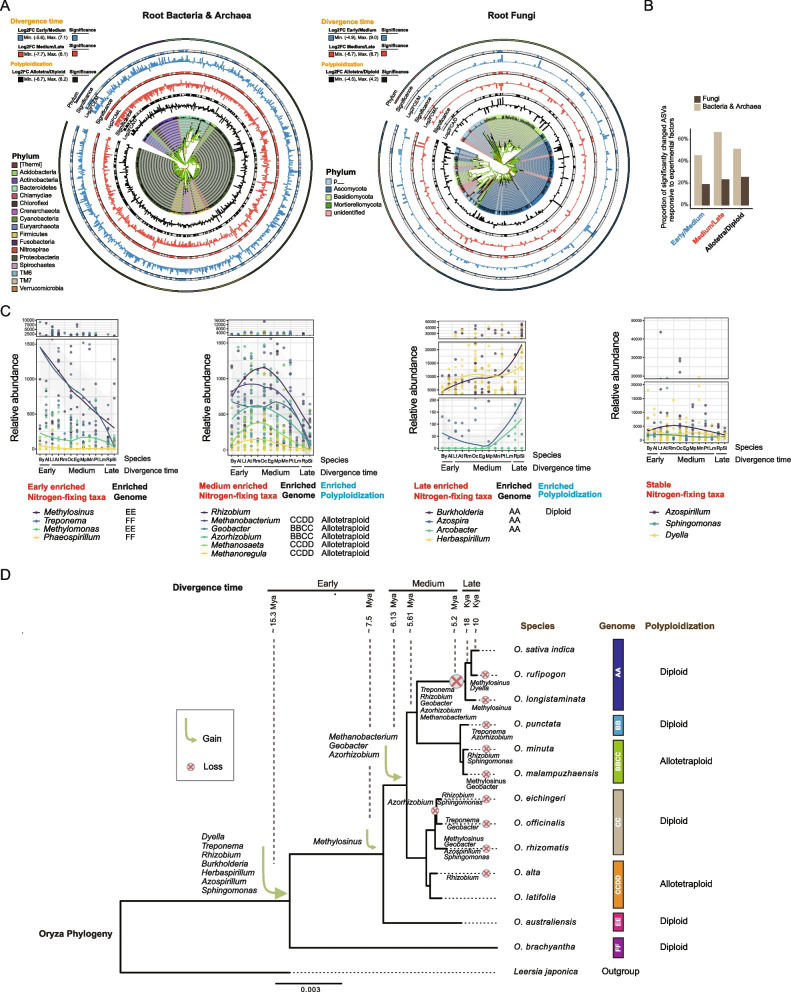
Potential root microbial function taxa responsive to *Oryza* evolution. **A** Phylogenetic tree showing fold-changes in the relative abundance of ASVs between different *Oryza* evolutionary phases and between diploid and allotetraploid species. Bar charts indicate log-transformed fold-changes, and the heatmap indicates statistical significance (p.adj < 0.05, DESeq); gray indicates non-significant changes. Outer circles and branch colors denote major phyla. Phylogenetic trees for significant ASVs were constructed and visualized using IQ-TREE and iTOL. **B** Proportion of bacterial and fungal ASVs that vary significantly between rice evolutionary time and ploidies, based on the significance values from **A**. **C** Dynamics of differential abundance of nitrogen-fixing bacterial taxa across *Oryza* species. Their potential functions were inferred using three complementary approaches: phylogenetic placement, prior literature, and FAPROTAX predictions, rather than by direct experimental verification. The plot shows the relative abundance of putative nitrogen-fixing bacterial genera that were significantly enriched in specific host species (identified by LEfSe and DESeq2). Species are ordered by their divergence time and labeled by their genome type. Species abbreviations: By (*O. brachyantha*), Al (*O. australiensis*), Lt (*O. latifolia*), At (*O. alta*), Rm (*O. rhizomatis*), Oc (*O. officinalis*), Eg (*O. eichingeri*), Mp (*O. malampuzhaensis*), Mn (*O. minuta*), Pt (*O. punctata*), Lm (*O. longistaminata*), Rp (*O. rufipogon*), Si (*O. sativa indica*). **D** Phylogenetic relationships of *Oryza* species, with parsimony-inferred shifts in the microbiota over *Oryza* evolution. Only gains and losses of the *Oryza*-specific lineages are plotted. Taxa with <1% abundance and <20% prevalence are considered losses

Phylogenetic analysis demonstrated that the significantly changed lineages were distributed throughout all mainly bacterial and fungal phyla (*p* < 0.05, DESeq) (Fig. 4A). We acknowledge that bacterial and fungal communities are profiled using different marker genes, which differ in taxonomic resolution and detection efficiency. Therefore, direct comparisons of absolute abundance or richness between kingdoms are not appropriate. Instead, we focused on the relative proportion of ASVs responding to host factors within each kingdom. Using this approach, we consistently observed that a higher proportion of bacterial ASVs varied in response to host genetic factors—including divergence time, ploidy, genome type, complex type, and species—compared with fungi (*p* < 0.05, GLM) (Fig. 4B, S14), indicating that bacterial communities are more strongly structured by host genetics in our system.

We found that several members of methanotrophs, including *Methylosinus*, *Methylomonas*, and *Methylocaldum*, were enriched in the early-diverged *Oryza* species, which were mainly the EE-genome (*p* < 0.05, LEfSe) (Figs. S15–S18). Several members of methanogens, including *Methanobacterium*, *Methanosaeta*, and *Methanoregula*, were enriched in the medium-diverged *Oryza* species, which were mainly allotetraploid *Oryza* species belonging to the CCDD-genome (*p* < 0.05, LEfSe). Several members of methanol oxidation, including *Methylotenera* and *Microvigula*, were enriched in the late-diverged *Oryza* species, which were mainly the AA-genome (*p* < 0.05, LEfSe).

We tested potential nitrogen-fixing taxa associated with *Oryza* speciation [43–46]. Interestingly, the different members of the potential nitrogen-fixing taxa were enriched in *Oryza* species with different divergence times or ploidy (Fig. 4C, S16–S18). *Methylosinus*, *Treponema*, *Methylomonas*, *Phaeospirillum*, and *Dyella* were enriched in the early-diverged *Oryza* species, which were mainly either EE-genome or FF-genome (*p* < 0.05, LEfSe). *Rhizobium*, *Methanobacterium*, *Geobacter*, *Azorhizobium*, *Methanosaeta*, and *Methanoregula* were enriched in the medium-diverged *Oryza* species, which were mainly allotetraploid *Oryza* species belonging to either the BBCC-genome or the CCDD-genome (*p* < 0.05, LEfSe). *Burkholderia*, *Azospira*, *Arcobacter*, and *Herbaspirillum* were enriched in the late-diverged *Oryza* species, which were mainly the AA-genome (*p* < 0.05, LEfSe).

In addition, several bacterial taxa were identified as differentially abundant between life cycle types (*p* < 0.05, LEfSe). Notably, *Burkholderia*, which is involved in nitrogen fixation, was enriched in annual species, while no significant life cycle-related patterns emerged for other functional taxa (Fig. S19).

Assuming that microbial communities are highly heritable, shifts in the root microbiome during the *Oryza* evolution can be estimated by mapping the community compositions of extant *Oryza* species onto an *Oryza* phylogeny. Here, we observed the shifts of the potential nitrogen-fixing taxa over the *Oryza* evolution (Fig. 4D). Gains of potential nitrogen-fixing taxa were detected between ~15.3 and ~6.13 Mya. However, a severe loss of potential nitrogen-fixing taxa was detected at ~5.2 Mya, which mainly were the late-diverging *Oryza* species.

### Co-speciating bacteria are more associated with grain weight

**Fig. 5:**
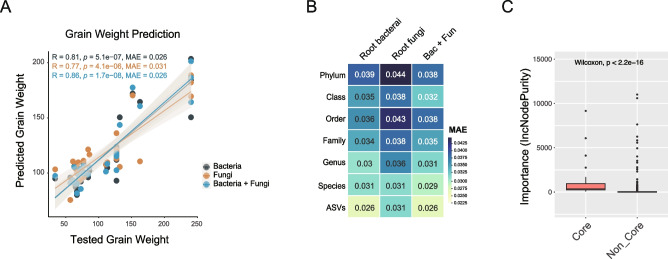
Grain weight was linked to the root microbiome. **A** Prediction of grain weight using the bacterial and fungal ASV data using the RF model. A line fit to the data is also shown using Spearman’s correlation. The gray areas denote 95% confidence intervals. A lower MAE value indicated higher prediction accuracy. **B** The prediction accuracy of grain weight regression models was dependent on different bacterial and fungal taxonomic levels. **C** Random forest mean predictor importance of core and non-core bacterial taxa for grain weight

To evaluate the agronomic relevance of root microbiome variation observed across *Oryza* evolution, we assessed whether microbial community features could predict grain weight in cultivated rice. We noticed that bacteria ASVs (the mean absolute error (MAE) = 0.026) were more accurate than fungi ASVs (MAE = 0.031) (Fig. 5A, B). Moreover, for both fungi and bacteria, the ASV data provided the strongest ability to predict the grain weight among taxonomic levels (Fig. 5B).

In addition, we assessed bacteria–fungi–grain weight interactions using the SparCC network. Grain weight was associated with 13 nodes in the correlation network, with two fungi and 11 bacteria (Fig. S20, Table S9). The correlations between microbial ASVs and grain weight were primarily positive (76.9%). These results confirmed that grain weight was more correlated with bacteria than fungi, and these correlations were primarily positive.

Next, we identified 36 core bacteria ASVs covering 20 genera (Fig. S21). We found that core bacteria, as opposed to non-core taxa, made a major contribution to the prediction of grain weight (Fig. 5C). The results indicated that bacterial ASV data can accurately predict more grain weight than fungal ASV data, with the main contribution coming from core bacteria.

Further analysis of the stability and niche breadth index of core bacteria of wild rice showed that, based on PCoA, non-core bacteria taxa clustered more significantly according to divergence time and polyploidy (Fig. S22), indicating that non-core bacteria taxa contributed more to evolution time and polyploidy variation, whereas core bacteria taxa were relatively ecologically stable. The niche width of core bacteria of wild rice was significantly higher than that of non-core bacteria (*p* < 0.05, Wilcoxon test) (Fig. S23), illustrating that core bacteria taxa of wild rice could adapt to a wider ecological niche.

### Growth-promoting effect of root core bacteria and potential nitrogen-fixing taxa of wild rice on cultivated rice

Building on our evolutionary framework across the *Oryza* genus (Fig. 1), we showed that host divergence time was a major determinant of root microbiome assembly, with clear phylosymbiosis patterns in bacterial communities (Figs. 2 and 3). Despite substantial host evolutionary divergence, a subset of bacterial taxa remained consistently associated with wild rice roots, forming a conserved core microbiome enriched in functional groups such as nitrogen fixation and methane metabolism (Fig. 4). These findings suggest that host–microbiome co-evolution has preserved ecologically stable and functionally relevant microbial assemblages over millions of years of *Oryza* evolution.

Given that these core root bacteria were also the strongest predictors of grain weight (Fig. 5C), we hypothesized that the conserved core microbiome of wild rice harbors collective growth-promoting functions. To test this hypothesis and to disentangle the effects of ecological conservation versus functional specialization, we conducted a controlled synthetic community (SynCom) experiment using a dual-pipeline design.

The SynCom was constructed from wild rice core bacteria, defined as ASVs that were both highly abundant (top 10% in relative abundance) and ubiquitous (present in >50% of all root samples) (Table S10) [47]. Importantly, the resulting 19-strain core SynCom represents a functional competent microbiome, as it includes multiple nitrogen-fixing taxa identified in Fig. 4 (e.g., *Herbaspirillum*, *Burkholderia*, *Sphingomonas*, and *Dyella*), thereby retaining key functional capacities characteristic of wild rice roots. In parallel, four potential nitrogen-fixing strains enriched in early- and medium-diverging *Oryza* species (Fig. 4C) were selected as functional specialists. This dual-pipeline design allowed us to disentangle the growth-promoting contributions of a conserved core microbiome from those of individual functional taxa.

**Fig. 6:**
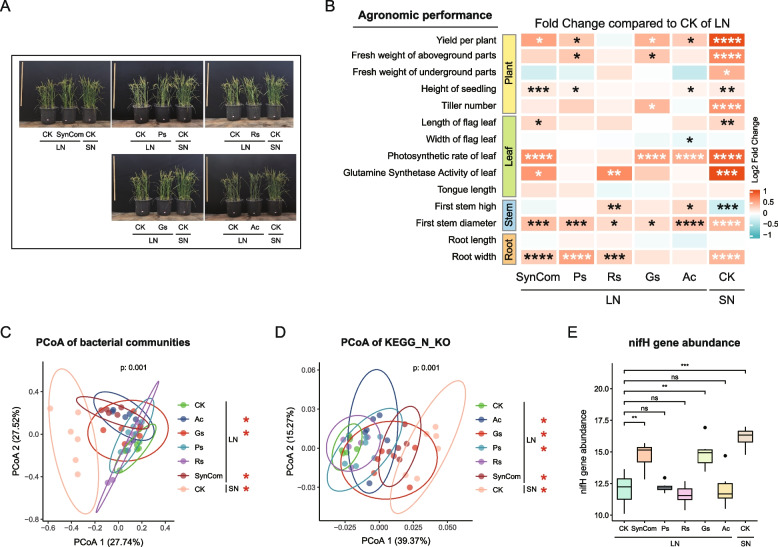
Growth-promoting effect of SynCom inoculation and single inoculation of four nitrogen-fixing bacteria on cultivated rice. **A** Under low-nitrogen condition, SynCom (synthetic communities) inoculation and single inoculation of the four potential nitrogen-fixing strains. CK: control, SynCom: synthetic community, Ps: *Phaeospirillum* NC6, Rs: *Rhizobium* NC2, Gs: *Geobacter sulfurreducens* NC8, Ac: *Azorhizobium caulinodans* NC7. LN: low-fertility soil; SN: normal-fertility soil. Fertilizer application rates are detailed in Table S12. **B** Effects of rhizosphere soil inoculated with SynCom and four nitrogen-fixing taxa on cultivated rice traits under low-nitrogen conditions. **C** Metagenome showed PCoAs of bacterial communities based on the Bray–Curtis distance matrix in the rhizosphere soil inoculated with SynCom and four nitrogen-fixing taxa on cultivated rice under low-nitrogen conditions. Asterisk denotes significance compared with uninoculation control (CK) of low-fertility rhizosphere soil: pairwise PERMANOVA, *p < 0.05. **D** Metagenome showed PCoAs of KEGG nitrogen metabolism functional groups based on the Bray–Curtis distance matrix in the rhizosphere soil inoculated with SynCom and four nitrogen-fixing taxa on cultivated rice under low-nitrogen conditions. Asterisk denotes significance compared with uninoculation control (CK) of low-fertility rhizosphere soil: pairwise PERMANOVA, *p < 0.05. **E** Metagenome showed nitrogenase-encoding *nifH* gene relative abundance of the rhizosphere microbial communities in the rhizosphere soil inoculated with SynCom and four nitrogen-fixing taxa on cultivated rice under low-nitrogen conditions. Asterisk denotes significance: *t*-test, *p < 0.05, **p < 0.01, ***p < 0.001, ****p < 0.0001

We conducted pot experiments to study the growth-promoting potential of the single potential nitrogen-fixing strains and SynCom for cultivated rice (*Oryza sativa* L. subsp. *japonica* cv. Zhonghua 11) in low-fertility soil (Fig. 6A, Tables S11, S12). Among these agronomic traits, photosynthetic rate (Pn), first stem diameter, and root width were most improved by strain inoculation (Fig. 6B). Compared with uninoculated plants in low-fertility soil, SynCom inoculation significantly improved all three traits (*p* < 0.001), and a single inoculation of the four potential nitrogen-fixing strains showed significant promoting effects on some of these traits. Inoculation with SynCom, *Rhizobium* sp. (Rs), *Geobacter sulfurreducens* (Gs), and *Azorhizobium caulinodans* (Ac) significantly increased yield per plant, with SynCom showing the strongest growth-promoting effect. Inoculation with *Phaeospirillum* sp. (Ps) and *Geobacter sulfurreducens* (Gs) significantly increased the fresh weight of the aboveground parts. Inoculation with SynCom and *Rhizobium* sp. (Rs) significantly increased the activity of glutamine synthetase in leaves. The results showed that SynCom inoculation and single inoculation of four nitrogen-fixing bacteria showed different degrees of growth-promoting effects on cultivated rice, and SynCom had the strongest growth-promoting effect.

Next, we analyzed the effects of SynCom inoculation and single inoculation of four nitrogen-fixing bacteria on the function of the rhizosphere microbiome using metagenome. PCoAs of bacterial community structure showed that SynCom, *Geobacter sulfurreducens* (Gs), and *Azorhizobium caulinodans* (Ac) inoculation differ significantly compared with the uninoculated control in low-fertility rhizosphere soil (*p* < 0.001, PERMANOVA; *p* < 0.05, pairwise PERMANOVA) (Fig. 6C). PCoAs of KEGG nitrogen metabolism (KEGG_N) functional groups showed that all microbiome inoculations except *Rhizobium* sp. (Rs) differ significantly compared with the uninoculated control in low-fertility rhizosphere soil (*p* < 0.001, PERMANOVA; *p* < 0.05, pairwise PERMANOVA) (Fig. 6D). The analysis of metagenomic short reads indicated that *nifH* gene abundance was significantly increased in rhizosphere soil with SynCom and *Geobacter sulfurreducens* (Gs) inoculations compared with uninoculation control in low-fertility rhizosphere soil (*p* < 0.05) (Fig. 6E).

### The nutrient strategy and keystone taxa of root core bacteria of wild rice

**Fig. 7:**
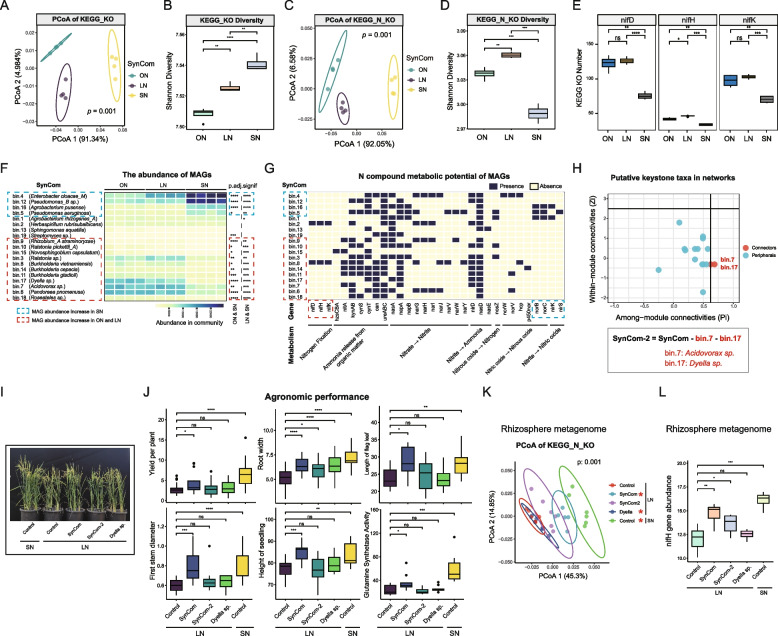
Nutrient acquisition strategy and keystone taxa of the core bacterial community. **A** PCoAs of KEGG functional groups based on the Bray–Curtis distance matrix in the metagenomic data under three nitrogen concentrations. ON: 0 mM NH_4_NO_3_; LN: 0.1 mM NH_4_NO_3_; SN: 1.0 mM NH_4_NO_3_. **B** Shannon diversity metric applied to KEGG functional groups in the metagenomic data. **C** PCoAs of KEGG nitrogen metabolism functional groups based on the Bray–Curtis distance matrix under three nitrogen concentrations in the metagenomic data. **D** Shannon diversity metric applied to KEGG nitrogen metabolism functional groups in the metagenomic data. **E** Nitrogenase-encoding *nifHDK* gene abundance of the microbial communities in the metagenomic data. **F** The abundance of the metagenome-assembled genomes (MAGs). **G** Nitrogen metabolic potential of the MAGs. **H** Putative keystone taxa were identified based on the node topological roles in networks in the metagenomic data. **I** Microbiota were inoculated in the rhizosphere soil of cultivated rice in low-fertility soil. SynCom-2: two connectors (*Dyella *sp. and *Acidovorax *sp.) were removed from SynCom. LN: low-fertility soil; SN: normal-fertility soil. Fertilizer application rates are detailed in Table S12. **J** Growth-promoting effect of different treatments on cultivated rice. **K** Metagenome showed PCoAs of KEGG nitrogen metabolism functional groups based on the Bray–Curtis distance matrix. Asterisk denotes significance compared with uninoculation control in low-fertility rhizosphere soil: pairwise PERMANOVA, *p < 0.05. **L** Metagenome showed nitrogenase-encoding *nifH* gene abundance of the rhizosphere microbial communities. Asterisk denotes significance: *t*-test, *p < 0.05, **p < 0.01, ***p < 0.001, ****p < 0.0001

To understand the nutrient acquisition strategy of root core bacteria of wild rice, we cultured SynCom in serum bottles under different nitrogen concentrations (Tables S10, S11). Comparisons of KEGG functional groups in the SynCom metagenomes revealed that functional groups of SynComs were significantly altered by nitrogen concentration (*p* < 0.002, PERMANOVA; *p* < 0.05, pairwise PERMANOVA) (Fig. 7A) and SynComs with a higher nitrogen concentration had a greater functional group diversity (*p* < 0.05, Wilcoxon) (Fig. 7B). Notably, when comparing the KEGG nitrogen metabolism (KEGG_N) functional groups, we found that the microbiotas of ON and LN (nitrogen limitation conditions) have a larger ability to utilize nitrogen than those of SN (*p* < 0.05, Wilcoxon) (Fig. 7C, D). The analysis of metagenomic short reads confirmed that *nifHDK* genes were significantly enriched in ON and LN (*p* < 0.05) (Fig. 7E). Furthermore, genome-resolved analysis revealed that all 18 metagenome-assembled genomes (MAGs) were present in all nitrogen concentrations, but their abundance was altered by nitrogen concentration (Fig. 7F). Under nitrogen-limitation conditions (ON and LN), nine MAGs (bin.8, bin.14, bin.17, bin.6, bin.7, bin.11, bin.18, bin.10, and bin.15) were significantly enriched (*p* < 0.05). As expected, among the nitrogen-limitation enriched MAGs, bin.8 (*Burkholderia vietnamiensis*) harbored the nitrogenase-encoding *nifHDK* genes (Fig. 7G). The results suggested that SynComs may rapidly adjust their nitrogen metabolism to cope with the nitrogen limitation conditions and maintain relatively ecological stability.

Co-occurrence network analysis detected two connectors in SynCom belonging to *Dyella* sp. and *Acidovorax* sp. (Fig. 7H). Moreover, we found that the two connectors were significantly enriched under nitrogen limitation conditions (Fig. 7F). To test the functional importance of the two connectors, we removed two connectors (*Dyella* sp. and *Acidovorax* sp.) from SynCom and constructed a new synthetic community (SynCom-2). SynCom-2 was inoculated into the rhizosphere soil of cultivated rice in low-fertility soil using pot experiments (Fig. 7I). SynCom inoculation significantly improved rice yield, flag leaf length, first stem diameter, height of seeding, and glutamine synthetase activity of leaf, while SynCom-2 inoculation did not show growth-promoting effects on these traits (*p* < 0.05) (Fig. 7J). In addition, inoculation with nitrogen-fixing bacteria *Dyella* sp. alone did not show obvious growth-promoting effects on these traits. The result indicated that *Dyella* sp., as a key member of SynCom, plays an important role in promoting rice growth rather than through its nitrogen-fixing function. PCoAs of KEGG nitrogen metabolism (KEGG_N) functional groups based on metagenome data showed that SynCom inoculation differed significantly compared with the uninoculated control in low-fertility rhizosphere soil, but SynCom-2 inoculation did not (*p* < 0.001, PERMANOVA; *p* < 0.05, pairwise PERMANOVA) (Fig. 7K). The analysis of metagenomic short reads indicated that *nifH* gene abundance was significantly increased in rhizosphere soil with SynCom and SynCom-2 inoculations compared with uninoculated control in low-fertility rhizosphere soil (*p* < 0.05), but the *nifH* gene abundance of SynCom inoculation was higher than that of SynCom-2 inoculation (Fig. 7L). The results suggested that SynCom requires the cooperation of its members to effectively play a performance growth-promoting function in cultivated rice, and single bacteria or the absence of key members in SynCom greatly reduces the growth-promoting effect.

### Genomic and phenotypic safety assessment of key SynCom strains

Although *Pseudomonas* sp. WN26, *Burkholderia* sp. CD4, and *Burkholderia* sp. W1 originate from genera that include opportunistic pathogens, targeted PHI-base screen confirmed that none of these isolates harbors the core virulence genes required for initiating plant disease (Table S13). To validate this prediction experimentally, we inoculated axenic rice seedlings grown in autoclaved soil under both low- and standard-nitrogen conditions, alongside uninoculated plants and two non-pathogenic controls (*Rhizobium* W17 and *Herbaspirillum* HS). After 6 days, seedlings inoculated with WN26, CD4, or W1 remained uniformly healthy and phenotypically indistinguishable from the controls, with no disease symptoms observed (Figs. S24 and S25). Together, these genomic and in-planta assessments provide strong evidence that all three isolates are non-pathogenic environmental strains suitable for inclusion in the SynCom.

## Discussion

### Is there co-evolution between the root microbiome and the genus *Oryza*?

The success of using crop microbiomes is driven by eco-evolutionary interactions between crop species and the microbiome. In agriculture, it is necessary to determine how crop microbial communities are structured and whether the host-associated microbiome has shifted as a result of crop speciation, including polyploidization and domestication events. However, few studies have analyzed the effects of multiple host genetic factors on bacteria and fungi during crop speciation in the same crop. The genus *Oryza* consists of 27 species, 4 complexes, 11 genome types, and 2 chromosome types, allowing us to address how *Oryza* speciation shapes microbial communities [1–3].

Our findings support the emerging view that crop genotype strongly shapes the root microbiome [48–51], and the evolutionary history of the genus *Oryza* explains a substantial portion of microbiome variation (Fig. 2A). In the root compartment, host speciation exerted a greater effect on bacterial than on fungal communities (Figs. 2, 3, and 4). Interestingly, root bacterial alpha diversity and community dissimilarity were significantly reduced as the genus *Oryza* evolved into the young AA clade, particularly following domestication (Fig. 2B, D). This domestication signal was striking: the microbiome differences between cultivated *O. sativa* ssp. *indica* and its wild AA-genome relatives alone (*R*^2^ = 25.7%) exceeded the variation explained by deep evolutionary divergence across the genus (*R*^2^ = 4.6–8.6%) (Fig. 2A, S8). Together, these results reveal a hierarchical pattern in which ancient evolutionary divergence establishes broad microbial differences, while recent human-driven selection acts as a rapid secondary filter, reshaping the root microbiome on a timescale comparable to millions of years of natural evolution. This intensive filter likely underlies the increased phylogenetic clustering observed in bacterial communities of late-diverging *Oryza* species, a pattern not observed in fungal communities (Fig. 3D). A phylogenetic clustering pattern can be explained by the phylogenetic aggregation of closely related taxa formed by a strong selection of important traits that facilitate adaptation to a particular environment [52]. Consequently, the phylogenetic clustering pattern of bacterial communities could be partially explained by the fact that phylogenetically clustered bacterial communities were prone to occur under strong selection forces related to the host’s filtering processes, and host filtering forces on bacterial communities increased in late-diverged *Oryza* species. Nevertheless, some studies have shown that fungal communities are more strongly influenced by genetic factors of the host plant than bacterial communities [17]. Moreover, deterministic and stochastic processes co-drove bacteria assembly, while stochastic processes strongly drove fungi assembly across *Oryza* species (Figs. S12 and S13). These results illustrated that the host imposed greater *Oryza* selection on bacterial diversity and composition than did fungi in the evolution of *Oryza* [2].

The stochastic nature of fungal assembly was further underscored by the inter-annual variation we observed. Despite using a common garden approach, the root fungal community composition shifted dramatically between the 2015 (Ascomycota-dominated) and 2019 (Basidiomycota-dominated) sampling years (Fig. 1D, I, S5 and S6). This contrasts with the relatively consistent bacterial community structure across years, suggesting that fungal recruitment is more susceptible to environmental fluctuations, such as yearly variations in temperature, precipitation, or the regional airborne spore bank. This sensitivity highlights a potential challenge in harnessing plant-fungal associations for agriculture, as they may be less temporally stable than their bacterial counterparts.

To provide insight into the evolutionary history between *Oryza* and its microbiome, we observed whether phylosymbiosis has occurred in *Oryza* species. We identified a phylosymbiotic signal between *Oryza* and its root bacteria, but not fungi (Fig. 3C). A recent study provided evidence that the fungal community did not exhibit a clear relationship with host phylogeny across 592 plant species from different taxa, which were mainly composed of Dothideomycetes, Eurotiomycetes, Leotiomycetes, Sordariomycetes, and Tremellomycetes [53]. However, the different responses of bacteria and fungi to the host plant were not consistent. For example, plant-associated fungal communities are more strongly influenced by host genetic factors and plant breeding than bacterial communities in *Helianthus annuus* [17]. Phylosymbiosis could result from host and microbiome co-evolution and be deterministically assembled by host selection [2, 54]. Plant species employ different strategies to strongly affect their endophytes via the host immune system, genetic networks, and plant metabolites [55]. Moreover, our data show that the bacterial diversity and composition significantly decreased in late-diverging *Oryza* species (Figs. 2 and 3, S10), indicating intensified host filtering during *Oryza*–bacteria co-evolution. Given that domestication has markedly narrowed the genetic diversity of cultivated rice and led to the loss of many wild traits [8–11], a parallel loss of regulatory capacity to maintain a diverse ancestral microbiome is a plausible explanation for this pattern. Thus, intensified filtering may arise either from stronger host selection toward a simplified consortium or (as appears more consistently with the domestication syndrome) from a reduced capacity to sustain ancestral microbial diversity. Distinguishing between these hypotheses will require future mechanistic studies. If the “loss of regulatory capacity” hypothesis holds, it further highlights the agronomic value of wild rice microbiomes as a reservoir for reintroducing lost beneficial functions into modern varieties.

Congruent speciation events between host and root bacterial lineages reveal long-term associations that represent one way for symbiotic partners to develop functional dependencies that reciprocally impact their fitness. We identified the association between the root microbiome and grain weight and found that bacterial ASV data could well predict more accurately than fungal ASV data, perhaps suggesting a functional link between co-speciating bacteria and host phenotype (Fig. 5). Our work on root microbiomes across *Oryza* species provides evidence of the co-phylogenetic pattern between *Oryza* and bacteria, and the closer connection between *Oryza* trait and bacteria, which may result from their co-evolution. Our findings supported the existence of an *Oryza*–bacteria hologenome that influences *Oryza* traits.

The much weaker effects of polyploidy and life cycle, compared to the strong phylogenetic signal linked with host divergence time (Figs. 2 and 3, S14), indicated that the traits most relevant for shaping root microbiota are those that evolve slowly and remain conserved over long evolutionary scales. Polyploidization or annual–perennial shifts may influence physiology or resource allocation, but these changes appear insufficient to fundamentally restructure the root microbial niche. Instead, deeply conserved traits—such as root exudate chemistry, immune signaling pathways, or nutrient acquisition strategies—likely mediate the stable recruitment of bacterial partners, producing the strong phylosymbiosis observed across the *Oryza* lineage. This highlights that co-evolution, rather than recent trait evolution, plays a central role in determining microbial community assembly.

Finally, the compartment-level differences reinforced the contrasting ecological strategies of bacterial and fungal microbiomes. In bacteria, the root-reservoir pattern indicates that root traits act as a dominant filter (Fig. 1), supplying much of the aboveground community and creating vertical continuity across tissues, which provides a mechanistic basis for the strong bacterial phylosymbiosis observed here. This pattern extends the classical two-step root recruitment model, in which microbial diversity progressively decreases from soil to rhizosphere to root through host-mediated filtering and selective entry [56–59]. In *Oryza*, our data suggest that such host-mediated filtering continues from roots to shoots and leaves, producing nested bacterial communities across compartments. By contrast, fungal communities showed pronounced compartment specificity—especially the highly diverse leaf mycobiome—indicating that fungal assembly is largely shaped by stochastic recruitment from airborne spores and local microhabitats rather than host control [53, 60, 61]. The strikingly high fungal diversity observed in leaves suggests the presence of a distinct foliar mycobiome, which may play important roles in phyllosphere-associated functions, including plant defense—an aspect that remains underexplored in rice and its wild relatives [61]. These contrasting compartment patterns provide critical context for interpreting why host genetic effects were stronger and more phylogenetically structured in bacteria than in fungi. This weaker host filtering explains the limited fungal phylogenetic signal and underscores fundamentally different ecological strategies for bacteria and fungi in the *Oryza* holobiont, which in turn shapes how strongly each group tracks host evolutionary history.

### The importance of the wild rice microbiome in supporting cultivated rice growth

The crop microbiome is considered to hold vast potential to support food production [56, 62–64]. However, we still know very little about how microbial mixtures can help us support crop growth. Microbiota from their wild relatives will be a promising strategy to support crop growth and production, especially in poor soils.

First, it is important to know what potentially beneficial microbial symbionts have been lost or gained during crop speciation, including polyploidization, in agriculture. Previous studies have shown that even between the two main types of Asian cultivated rice, *Oryza sativa* spp. *indica* and *Oryza sativa* spp. *japonica*, the enriched nitrogen metabolism taxa and the nitrogen-use efficiency (NUE) were different [65]. Wild rice contains various valuable traits, including high NUE and large biomass on poor soils [11, 66]. Wild rice relied heavily on microbial symbionts to provide it with growth-limiting nutrients [18, 67]. We observed that some potentially nitrogen-fixing taxa varied during *Oryza* speciation (Fig. 4C, S15–S19), and a dramatic loss of potential nitrogen-fixing taxa was detected at a divergence time of ~5.2 Mya (Fig. 4D). However, fungi did not experience the same dramatic loss during *Oryza*’s evolutionary processes, and alpha diversity of the fungal community significantly increased over evolutionary time (Fig. 2, S10). These results lead us to speculate that root bacteria likely experienced a depletion event in the recently diverged *Oryza* species (~5.2 Mya), but fungi did not. This is not good news for modern agriculture, and the loss of nitrogen-fixing bacteria leads to the need for more nitrogen fertilizers in modern rice cultivation.

Another important agricultural event is *Oryza* polyploidization [68]. Here, we provided evidence that *Oryza* polyploidization shaped root bacterial and fungal structures (Fig. 2A), and the proportion of bacteria significantly changing ASVs was considerably higher than that of fungi (Fig. 4A, B), although *Oryza* polyploidization did not significantly alter the alpha diversity of bacterial and fungal communities (Fig. 2E, S10). A major physiological change in polyploids is thought to be an increased demand for growth-limiting nutrients [69]. However, the mechanism by which polyploidy affects the crop microbiomes is largely unknown. There is evidence indicating that polyploidy might increase mutualistic associations, such as legume–rhizobium interactions [23, 70], and arbuscular mycorrhizal fungi (AMF) interactions in *Heuchera cylindrica* [69]. We revealed that potentially nitrogen-fixing taxa, such as *Pleomorphomonas*, *Treponema*, *Geobacter*, and *Azorhizobium*, and methanogens, including *Methanobacterium*, *Methanosaeta*, and *Candidatus Methanoregula*, were enriched in polyploidy *Oryza* (Fig. 4C, S15, S18). These results suggest that polyploidy *Oryza* might alter belowground nutritional mutualisms with crops by increasing mutualistic associations.

Accumulating evidence reveals that host–microbiome co-evolution could promote cooperation [39]. Moreover, host control mechanisms suggested that cooperative evolution can occur when host control is effective [36]. Our data revealed that bacteria coevolved with *Oryza* and host more efficiently control bacteria, bacterial community exhibited a significant phylosymbiosis with their host, but fungi did not (Fig. 3). Deterministic and stochastic processes co-drove bacteria assembly, and deterministic processes gradually increased from early- to late-diverged *Oryza* species, while stochastic processes strongly drove fungi assembly across *Oryza* species (Figs. S12 and S13). These results indicated that bacteria may play more important ecological roles in *Oryza*’s agronomic performance than fungi. Strikingly, our data showed that the bacterial ASVs could provide a higher prediction accuracy for grain weight than fungal ASVs (Fig. 5). Network analysis further confirmed stronger bacterial correlations with grain weight than with fungi, and these correlations were primarily positive (Fig. S20). These results suggest that more cooperation emerged between the genus *Oryza* and bacteria. We found that the introduction of host control mechanisms could explain why there was more cooperation between *Oryza* and co-speciating bacteria than between *Oryza* and fungi during co-evolutionary processes in our data, even from an ecological perspective. Here, we think co-speciating bacteria of wild rice represent a unique opportunity to support food provision and plant-based industries.

To reduce nitrogen fertilization and improve cultivated rice productivity, it is critical to construct a simple and functional SynCom that considers the relationships and interactions among strains. Many available commercial microbial products rely on easy-to-culture microorganisms, with traits such as indoleacetic acid production, and siderophore secretion, nitrogen fixation, or phosphorus solubilization, often applied individually rather than as interacting communities [62, 71]. Such products frequently fail to consider interactions among strains, which can limit functional expression in natural or agricultural settings.

In contrast, co-speciating bacteria from wild rice offer a unique opportunity to support cultivated rice growth. Core bacteria of wild rice have co-evolved with their hosts for tens of millions of years. The combination of core bacteria results from natural selection of relationships and interactions between the constituent strains. It has been previously reported that core microorganisms boost plant production, and plant core microbiomes harbor the potential to promote nutrient turnover in impoverished substrates of a Brazilian biodiversity hotspot [62, 72]. Similarly, in grasslands and boreal forests, nitrogen-fixing bacteria were the main contributors to the total nitrogen acquired by the vegetation [73].

Building on our insights into compartment-specific microbiome assembly, we experimentally tested the functional relevance of co-speciating bacterial taxa predicted from phylogenetic analyses (Fig. 4). The synthetic community (SynCom), constructed from wild rice core bacteria and nitrogen-fixing specialists, promoted rice growth more effectively than individual strains, revealing synergistic interactions within the core community (Fig. 6). In vitro assays further revealed that the SynCom dynamically adjusted its composition and maintained nitrogen fixation under low-nitrogen conditions, with key taxa such as *Acidovorax* and *Dyella* playing pivotal roles. Notably, removal of two critical strains markedly reduced the growth-promoting effect, underscoring the importance of both functional taxa and their interactions within the core community (Figs. 6 and 7).

Together, these findings link host evolutionary history, root-mediated microbial assembly, and trait-associated microbial functions. Co-speciating bacterial genera are closely associated with crop traits, and SynComs derived from wild rice provide a tractable system to explore the functional ecology of host-associated microbiomes. Our work lays the foundation for leveraging the conserved and co-evolved microbiomes of wild relatives to enhance sustainable crop production, highlighting how evolutionary and ecological processes jointly shape plant–microbe interactions.

### Limitations and future perspectives

The field-based and observational nature of this study emphasizes ecologically realistic associations among host genotypes, microbial taxa, and plant traits within a defined environmental context. This framework is well suited for detecting robust host-associated microbiome patterns under natural conditions; however, the extent to which these relationships are conserved across contrasting soils, climates, and management regimes remains to be evaluated. Systematic assessment of the stability and reproducibility of host–microbiome associations across environmental gradients will therefore be essential for broader generalization.

Advancing toward a predictive and mechanistic understanding of crop–microbiome interactions will require methodological approaches that more directly integrate microbial composition, function, and host traits. First, robust interpretation of microbial function demands integrative evidence, as signals inferred from soil gene abundance (e.g., nifH) can be confounded by community turnover and environmental variability; controlled SynCom assays provide proof-of-concept, but future studies should extend to gnotobiotic systems, semi-natural microcosms, and field trials to connect mechanistic insights with ecosystem outcomes. Second, resolving strain-level dynamics is critical, as conventional metagenomics cannot distinguish introduced strains from closely related residents; strain barcoding, reporters, high-depth meta-transcriptomics, and single-cell genomics will be essential to track SynCom members in situ. Third, correlating microbiome dissimilarity with host genetic distance at the genome-wide SNP level would refine species-level frameworks and quantify how host divergence shapes microbial communities. Finally, for fungi, ITS limitations warrant using more stable loci (e.g., *LSU*, *RPB1*, *RPB2*, *TEF1*) to build robust evolutionary frameworks. Collectively, these methodological directions will enable predictive, mechanistic, and translational insights into crop–microbiome interactions.

## Methods

### Plant cultivation, sample collection, and processing

These *Oryza* seeds came from the International Rice Research Institute (IRRI) (https://www.irri.org/). The genus *Oryza* consists of 18 species (17 wild and 1 cultivated), 3 complexes, 7 genome types, and 2 chromosome types (Fig. 1A, Table S1). All plants were cultivated under standardized common-garden conditions at the Jiangxi Agricultural Institute Station in Sanya, Hainan Province, China (18°21′N, 109°44′E). All plants were grown with uniform irrigation, fertilization, and field management. The experiment followed a randomized block design, with 1-m^2^ plots separated by 30-cm buffer zones to minimize cross-interference among accessions.

We conducted two major sampling campaigns. In 2015, we profiled root, shoot, and leaf endophytic communities, collecting 108 independent tissue samples from 40 *Oryza* accessions. In 2019, we focused on root endophytes and collected 124 independent root samples from 59 accessions, covering all major *Oryza* lineages. Detailed sampling structure is provided in Supplementary Methods.

All harvested tissues were processed using a standardized endophyte-enrichment workflow. Roots were washed to remove adhering soil, and all tissues were surface-sterilized (70% ethanol 1 min, 0.3% NaOCl + 0.01% Tween-20 for 15 min, three sterile water rinses) [11, 66]. Tissue samples were stored at −80 °C until DNA extraction.

### DNA extraction and 16S rRNA and ITS gene sequencing

Genomic DNA was extracted from *Oryza* samples using the E.Z.N.A. HP Plant DNA Kit (Omega Bio-tek, Inc.). We characterized the bacterial and fungal communities by sequencing amplicons of the variable V4 region of the bacterial 16S rRNA gene with primer pairs 515F/806R and the ITS region of the fungal ITS gene with primer pairs ITS1F/ITS2 [74–76]. PCRs were performed with Phusion High-Fidelity polymerase under standard cycling conditions. Amplicons were checked on agarose gels, pooled, purified (Qiagen Gel Extraction Kit), and quantified using Qubit. Sequencing libraries were prepared using the TruSeq PCR-Free kit and sequenced on an Illumina NovaSeq platform to generate paired-end reads.

### Amplicon sequence processing and diversity analyses

Raw sequencing reads were provided by the sequencing company after removal of adapters and primers. The 16S rRNA and ITS gene sequences were processed using QIIME2 (v.2019.7) (https://qiime2.org/) and DADA2 [77]. ASVs were taxonomically assigned using Greengenes 13.8 (bacteria) and UNITE (fungi). ASVs assigned to chloroplasts or mitochondria were removed. ASV tables were normalized to 100,000 reads per sample.

Principal coordinate analysis (PCoA) was conducted with the Bray–Curtis distance metric using the “vegan” R package (v2.6.4). Differentially abundant ASVs were identified using LEfSe (v1.0.8) (LDA > 2, *p* < 0.05). Phylogenetic tree of significant ASVs was constructed with IQ-TREE (v2.0.3) and visualized using iTOL (https://itol.embl.de/). The machine learning framework employed was based on the “randomForest” R package (v4.7.1). The co-occurrence network in microbial communities was conducted using the “igraph” R package. The inferred correlations were restricted to those having abs(cor.r) > 0.6 and *p* < 0.05. We adopted criteria used in previous studies and identified module hubs (Zi ≥ 2.5, Pi < 0.62), connectors (Zi < 2.5, Pi ≥ 0.62), and network hubs (Zi ≥ 2.5, Pi ≥ 0.62).

Faith’s phylogenetic diversity (Faith’s PD) and the mean pairwise phylogenetic distance (MPD) were calculated for bacterial (16S rRNA) and fungal (ITS) communities using the picante R package (v1.8.2) [78]. For fungi, Faith’s PD and MPD metrics were calculated from a de novo phylogenetic tree, but due to the limited resolution of ITS for deep evolutionary relationships, these tree-based metrics reflected relative trends rather than exact phylogenetic distances. To complement phylogenetic metrics, an alignment-free, *k*-mer–based MPD-like metric was computed following Bokulich [79]. ASV sequences were decomposed into overlapping 16-mers using q2-kmerizer (QIIME2, v2024.10), transformed via TF–IDF normalization (5000 features for 16S rRNA, 10,000 for ITS), and sample-level, abundance-weighted MPD-like values were calculated as the mean pairwise cosine distance among ASVs within each sample. We quantified the relative influence of stochastic and deterministic processes using βNTI (999 randomizations) and the neutral community model (NCM).

### Host–microbiome phylogenetic congruence

Phylogenetic signal in microbial community traits was assessed using Blomberg’s *K* and Pagel’s *λ* implemented in the phytools R package (v0.7.90).

Microbial dendrograms were generated by hierarchical clustering of Bray–Curtis distances, and host phylogeny was reconstructed from *Oryza* chloroplast genomes (GenBank accessions KF359901–KF359922) [7]. Topological congruence between host and microbiota trees was quantified using a custom Python script from the Seth R. Bordenstein lab and TreeCmp (v1.0), employing normalized Robinson–Foulds and matching-cluster metrics with 100,000 random trees to evaluate significance [24, 25]. Congruence was further assessed using Clustering Information Distance (CID) with the TreeDist R package (v2.11.1), with significance determined via permutation testing (*n* = 9999) [80].

### Ancestral state reconstruction

A presence/absence matrix of potential nitrogen-fixing taxa was mapped against the *Oryza* phylogeny using Count (v.10.04) [81]. Taxa with <1% relative abundance and <20% prevalence were considered absent, and gains/losses across host lineages were inferred by asymmetrical Wagner parsimony with gain and loss penalties of 1.5 and 1, respectively.

### Bacteria isolation and genome sequencing

Bacterial strains were isolated from 2019 field-collected *Oryza* roots and rhizosphere soil using multiple culture media (Table S11). DNA from each strain isolation was extracted using the E.Z.N.A. Bacterial DNA kit (Omega Bio-tek, Inc). We characterized all isolated bacteria by sequencing amplicons of the bacterial 16S rRNA gene using the 27F/1492R primer pairs on the Sanger sequencing platform. The identity of each microbe was annotated with NCBI BLAST and RDP.

Genomes of strains used in SynCom and pot experiments were sequenced on Illumina platforms. Genome assembly was performed with Unicycler (v0.4.7) [82]. Genes were annotated using Prokka (v1.14.6) [83]. Genome quality was assessed using CheckM (v1.2.3) to evaluate completeness and contamination, and taxonomy was assigned with GTDB-Tk (v2.4.0) to ensure accurate phylogenetic placement [84, 85].

### Construction of the synthetic communities (SynComs)

We implemented a dual-pipeline SynCom strategy. We identified the core root microbiota present in microbial communities according to two criteria: (1) the abundant ASVs were the top 10% in terms of relative abundance across all root samples; (2) microbial ASVs present in more than 50% of individuals across *Oryza* accessions [47]. Representative strains of core bacteria of wild rice were used to construct synthetic communities (SynComs) (Table S10). In parallel, four nitrogen-fixing strains selected independently based on their phylogenetic enrichment in early- and medium-diverging *Oryza* species (Fig. 4D). Treatments included the Core SynCom, each nitrogen-fixer alone, and a non-inoculated control.

### Potted plant experiment

For pot experiments, rice plants (*Oryza sativa* L. subsp. *japonica* cv. Zhonghua 11) were grown in plastic pots containing 20 kg of red clay soil supplemented with defined amounts of nitrogen (N), phosphorus (P), and potassium (K) fertilizers (Table S12). The seeds of rice were surface-sterilized in 75% ethanol for 30 s and 2.5% sodium hypochlorite for 15 min, and washed for 5 min, five times, with sterile water. Seeds were allowed to germinate. When the rice grows to three leaves and one heart, they were transplanted to moist red soil that has been fertilized in advance. Five plants were planted in each pot, and four replicates are made for each different treatment.

Following isolation, bacterial strains were maintained on a standardized set of media for all subsequent experimental work (Table S14). The single-strain cultures were adjusted to a final OD_600_ of ~0.5. The SynComs were an equimolar mixture of strains with a final OD_600_ of ~0.5. Bacterial suspensions were added into rhizosphere soil before rice transplanting, and each plant received in total 2 ml of bacterial suspension. The plant samples and rhizosphere soil were all harvested at the mature stage.

### SynComs co-culture experiment using serum bottles

The SynComs were co-cultured in OS media using serum bottles by adding different concentrations of NH_4_NO_3_ (ON: 0 mM, LN: 0.1 mM, SN: 1.0 mM) for 48 h at 30 °C (Table S11).

### Metagenome sequencing and analysis

DNA was extracted using the E.Z.N.A. Soil DNA Kit (Omega Bio-tek, Inc). The metagenome of the rhizosphere microbiome in the pot experiment was sequenced on the Illumina NovaSeq. The metagenome of SynComs co-cultured in serum bottles was sequenced on the BGISEQ-T7 platform. Metagenome sequencing clean reads were assembled with MEGAHIT (v1.2.9) [86]. Genes were annotated using Prokka (v1.11) and consolidated into non-redundant gene references using CD-HIT (v4.8.1) [83, 87]. The open reading frames (ORFs) were predicted using Prodigal and annotated with EggNOG databases (v5.0.2) using Diamond (v2.0.15) [88]. Non-redundant gene catalogs were constructed using CD-HIT. Genome binning was co-assembled using MetaWRAP (v1.3.2) [89, 90]. Bin quality was assessed with CheckM (v1.2.3) [84]. Bin taxonomy was assigned with GTDB-Tk (v2.4.0) [85]. Abundances were estimated with CoverM (v0.7.0) [91].

### In silico* assessment of plant pathogenic potential*

Protein sequences from each strain were queried against the PHI-base database (v4.17) using BLASTP (v2.13.0+) with an *E*-value cutoff of 1e−5 and minimum query coverage of 50% [92, 93]. Homologs were identified using a ≥30% identity threshold, and high-confidence matches (≥70% identity) were further examined. These high-similarity genes were functionally classified according to PHI-base categories and reported in planta phenotypes (e.g., loss of pathogenicity, reduced virulence).

### Experimental validation of pathogenicity

The pathogenic potential of three selected strains (*Pseudomonas* sp. WN26, *Burkholderia* sp. CD4, and *Burkholderia* sp. W1) was assessed alongside two non-pathogenic control strains (*Rhizobium* sp. W17 and *Herbaspirillum* sp. HS) in an autoclaved soil assay under both low-nitrogen (LN) and standard-nitrogen (SN) conditions. Surface-sterilized rice seeds were germinated for 2 days, treated with bacterial suspensions (OD_600_ = 0.2) or sterile PBS (control), and transplanted into autoclaved soil. Plants were grown in a greenhouse for 6 days and monitored daily for disease symptoms. Detailed methods are provided in the Supplementary Materials.

### Statistical analysis

Permutational Multivariate Analysis of Variance (PERMANOVA) and pairwise PERMANOVA were performed using the adonis () and pairwise.adonis () function from the “vegan” R package (v2.6.4), respectively. Differential abundance was tested using DESeq2 (v1.32.0) (padj < 0.05) and generalized linear model (GLM) with negative binomial regression was fitted using the “mvabund” R package (v4.2.1). Pearson correlations, Wilcoxon test, and *t*-test were computed using the cor.test, Wilcox.test, and t.test() function from the R (v.4.1.3).

## Supplementary Information

Additional file 1. Supplementary figures. Figure S1 Alpha diversity varies across host compartments. Observed features indices of the roots, shoots, and leaves bacteria (A) and fungi (C). Chao1 indices of the roots, shoots, and leaves bacteria (B) and fungi (D). *p* values reflect the Wilcoxon test. Figure S2 The number of bacterial and fungal ASVs found exclusively in particular compartments. Venn diagrams illustrating bacterial (A) and fungal (B) ASVs shared among host compartments. Figure S3 The relative abundance of microbial taxa across host compartments at the genus level. Relative abundance of bacterial (A) and fungal (B) genera in the roots, shoots, and leaves. Figure S4 Inter-annual and host genetic effects on root microbiome composition. Principal coordinate analysis (PCoA) based on Bray–Curtis distances, performed on the subset of accessions (*n* = 10) sampled in both the 2015 and 2019 campaigns. (A) Samples colored by sampling year (2015 vs. 2019). (B) Samples colored by host evolutionary complex (Officinalis, Sativa, and unclassified groups). (C) Samples colored by host divergence time (early, medium, and late). Note: Because the 2015 and 2019 campaigns included largely non-overlapping accessions, only a limited number of accessions were sampled in both years. Figure S5 The relative abundance of bacterial taxa in the roots of 40 *Oryza* accessions. The relative abundance in the (A) phylum and (B) genus level of bacterial taxa. Figure S6 The relative abundance of fungal taxa in the roots of 40 *Oryza* accessions. The relative abundance in the (A) phylum and (B) genus level of fungal taxa. Figure S7 PCoAs of bacterial and fungal communities among different life cycle based on the Bray–Curtis distance matrix. Figure S8 PCoA of root microbiomes across host evolutionary scales. Principal coordinate analysis (PCoA) based on Bray–Curtis distances. (A) Differentiation between the Officinalis (non-AA genome) and Sativa (AA genome) complexes. (B) Differentiation within the AA-genome Sativa complex, comparing cultivated *Oryza sativa* ssp. *indica* with wild AA-genome relatives. Statistical significance (PERMANOVA) is indicated. Figure S9 The Bray–Curtis dissimilarity of the root bacterial and fungal communities across the host genotype. Figure S10 Faith’s PD index of root bacterial and fungal community across different host factors. Figure S11 The *k*-mer MPD-like metric of root bacterial and fungal communities derived from *k*-mer alpha diversity estimates. (A) The *k*-mer MPD-like metric of root bacterial communities, colored by divergence time, polyploidy types, and life cycle types. (B) *k*-mer MPD-like metric of root fungal communities, colored by divergence time, polyploidy types, and life cycle types. *k*-mers were generated using an n-gram size of *k* = 16. Figure S12 Deterministic and stochastic processes in root microbiome assembly using neutral community model (NCM) analysis. The fit of the NCM for community assembly. The predicted occurrence frequencies for bacterial and fungal communities. The solid blue lines indicate the best fit to the NCM, and the dashed blue lines represent 95% confidence intervals around the model prediction. ASVs that occur more or less frequently than predicted by the NCM are shown in different colors. *R*^2^ indicates the fit to this model. Figure S13 Deterministic and stochastic processes in root microbiome assembly using null model analysis. (A) The relative contribution of determinism and stochasticity to bacterial and fungal assembly based on the βNTI values. The percentages above and below the violin plot represented the proportion of stochastic and deterministic processes in microbiome assembly, respectively. (B) The relative contribution of determinism and stochasticity on bacteria and fungi assembly among *Oryza* evolutionary phases based on the βNTI value. (C) The relative contribution of determinism and stochasticity on bacteria and fungi assembly between diploid and allotetraploid based on the βNTI value. (D) The relative contribution of determinism and stochasticity on bacteria and fungi assembly between annual and perennial based on the βNTI value. Figure S14 The proportion of microbial ASVs responsive to host factors exhibited differential abundance. The proportion of bacterial and fungal ASVs responsive to host factors was shown (*p* < 0.05, GLM). Negative binomial generalized linear models tested whether individual ASV exhibited different abundance across experimental factors (divergence time, polyploidy, complex, genome, and species). Figure S15 The relative abundance of the potential different bacterial and archaeal function taxa across *Oryza* species among the different *Oryza* evolutionary phases based on LEfSe analysis. Their potential functions were inferred using three complementary approaches: phylogenetic placement, prior literature, and FAPROTAX predictions, rather than by direct experimental verification. Species abbreviations: By (*O. brachyantha*), Al (*O. australiensis*), Lt (*O. latifolia*), At (*O. alta*), Rm (*O. rhizomatis*), Oc (*O. officinalis*), Eg (*O. eichingeri*), Mp (*O. malampuzhaensis*), Mn (*O. minuta*), Pt (*O. punctata*), Lm (*O. longistaminata*), Rp (*O. rufipogon*), Si (*O. sativa indica*). Figure S16 LEfSe analysis revealed significant bacterial and archaeal genera differences among *Oryza* evolutionary phase. The genera belonging to methanogen were marked by green asterisks. The genera belonging to methanol oxidation were marked by orange asterisks. Figure S17 LEfSe analysis revealed significant bacterial and archaeal genera differences among *Oryza* genome types at the genus level. The genera belonging to methanotrophs were marked by blue asterisks. The genera belonging to nitrogen-fixing taxa were marked by red asterisks. The genera belonging to methanogen were marked by green asterisks. The genera belonging to methanol oxidation were marked by orange asterisks. Figure S18 LEfSe analysis revealed significant bacterial and archaeal genera differences between diploid and allotetraploid. The genera belonging to methanotrophs were marked by blue asterisks. The genera belonging to nitrogen-fixing taxa were marked by red asterisks. The genera belonging to methanogen were marked by green asterisks. The genera belonging to methanol oxidation were marked by orange asterisks. Figure S19 LEfSe analysis revealed significant bacterial and archaeal genera differences between annual and perennial. The genera belonging to nitrogen-fixing taxa were marked by red asterisks. Figure S20 Root microbial cross-kingdom network associated with grain weight. (A) SparCC correlation-based network of root microbial ASVs detected in the endosphere across the *Oryza* species. Nodes were color-coded by microbial type and host trait (grain weight). ASVs belonging to different microbial kingdoms had distinct color codes, and node size reflects their relative abundance (RA) (log10-transformed) in the root endosphere. ASVs associated with grain weight were marked by green asterisks. Edges between nodes correspond to either positive (black) or negative (red) correlations inferred from abundance profiles using the SparCC method (pseudo *p* < 0.05, correlation values > 0.4). Intra-kingdom correlations were represented with dash lines and interkingdom correlations by solid lines. Microbiota and grain weight correlations were represented with vertical slash lines. (B) The proportion of edges showing positive (black) or negative (red) correlations in the network. B, bacteria; F, fungi; G, grain weight. (C) Cumulative correlation edge number in the network between bacterial and fungal ASVs. Bacterial were grouped at the phylum level according to their cumulative correlation edge number with fungal ASVs. (D) Cumulative correlation edge number in the network between bacterial and fungal ASVs. Fungal were grouped at the phylum level according to their cumulative correlation edge number with bacterial ASVs. (E) Cumulative correlation edge number in the network between grain weight and microbial ASVs. Bacterial and fungal were grouped at the phylum level according to their cumulative correlation edge number with grain weight. Figure S21 The taxonomic distribution of root core and non-core bacterial taxa. (A) The taxonomic distribution of root core and non-core bacterial taxa at different genome type of *Oryza* species. The taxonomic distribution of root core and non-core bacterial taxa at the phylum (B) and genus (C) level. Figure S22 PCoAs of root core and non-core bacterial communities based on the Bray–Curtis distance matrix. PERMANOVA showed significant differences in community composition (*p < 0.05, **p < 0.01, ***p < 0.001). Figure S23 Mean habitat niche breadths (Bcom) of core and non-core bacterial communities. Figure S24 In situ growth of rice seedlings in the soil-autoclaved pathogenicity assay. (A) Seedlings grown in standard-nitrogen (SN) soil for 6 days. (B) Seedlings grown in low-nitrogen (LN) soil for 6 days. In both panels, from left to right: Uninoculated control (PBS-treated), and seedlings inoculated with *Pseudomonas* sp. WN26, *Burkholderia* sp. CD4, *Burkholderia* sp. W1, *Rhizobium* sp. W17, and *Herbaspirillum* sp. HS. Strains W17 and HS served as non-pathogenic controls. The soil was autoclaved prior to planting. All seedlings exhibited healthy aboveground morphology with no signs of disease after 6 days of growth in the greenhouse. Figure S25 Root systems of rice seedlings from the soil-autoclaved pathogenicity assay after gentle rinsing. (A) Seedlings grown under standard-nitrogen (SN) conditions for 6 days. (B) Seedlings grown under low-nitrogen (LN) conditions for 6 days. In both panels, from left to right: Uninoculated control (PBS-treated), and seedlings inoculated with *Pseudomonas* sp. WN26, *Burkholderia* sp. CD4, *Burkholderia* sp. W1, *Rhizobium* sp. W17, and *Herbaspirillum* sp. HS. Strains W17 and HS served as non-pathogenic controls. Soil was autoclaved prior to planting. All seedlings exhibited healthy aboveground morphology with no signs of disease after 6 days of growth in the greenhouse. Side-by-side comparison shows that all root systems were healthy and intact, with no observable morphological differences or disease symptoms (e.g., necrosis, discoloration) between inoculated seedlings and uninoculated controls. This uniform, healthy phenotype across treatments indicates that none of the tested bacterial strains caused pathogenic effects under these controlled conditions.

Additional file 2. Supplementary tables. Table S1 Rice accessions in the 2015 and 2019 growing season. Table S2 Overlap or specific bacterial and fungal ASVs across rice compartments. Table S3 The relative abundance of bacterial taxa was significantly affected by compartments using generalized linear model (GLM) analysis. Table S4 The relative abundance of fungal taxa was significantly affected by compartments using generalized linear model (GLM) analysis. Table S5 Pairwise permanova results across rice compartments. Table S6 The relative abundance of bacterial phylum associated with different Oryza species. Table S7 The relative abundance of fungal phylum associated with different Oryza species. Table S8 Pairwise permanova results in light of the host’s genetic background. Table S9 The SparCC network statistics. Table S10 Synthetic community (SynCom) composition. Table S11 Media composition. Table S12 Fertilizer application rates. Table S13 Summary of PHI-base plant pathogenicity analysis for key SynCom strains. Table S14 Media used for bacterial strain culture in SynCom experiments.

Additional file 3. Supplementary methods.

## Data Availability

All raw sequences derived from this project were submitted to the Short Read Archive of NCBI and can be found under the BioProject accession numbers PRJNA851112, PRJNA1000725, and PRJNA1271606.
