# Supplementary material for: Microbiome eco-evolution of cultivated and wild rice species across the genus Oryza and its importance in supporting rice growth
Source: Microbiome. 2026 Mar 6;14:83. doi: 10.1186/s40168-026-02359-z (PMC12973805; doi:10.1186/s40168-026-02359-z)
Supplement: Supplementary file 4 — Additional file 3. Supplementary methods. [file 40168_2026_2359_MOESM3_ESM.pdf]

Supplementary Methods

1. Plant Cultivation, Sample Collection and Processing

These *Oryza* seeds came from the International Rice Research Institute (IRRI). The genus *Oryza* consists of 18 species (17 wild and 1 cultivated), 3 complexes, 7 genome types, and 2 chromosome types (Fig. 1a, Table S1). All plants were cultivated under standardized common-garden conditions at the Jiangxi Agricultural Institute Station in Sanya, Hainan Province, China (18°21′N, 109°44′E). The site is characterized by a tropical maritime monsoon climate with an annual mean temperature of 25.7 °C and mean annual precipitation of 1347.5 mm, approximately 85% of which occurs from May to October. Plants were grown in the same paddy field with uniform management in a randomized block design (1 m^2 plots with 30 cm buffers).

Two major sampling campaigns were conducted. In the 2015 season, we profiled endophytic bacterial and fungal communities from roots, shoots and leaves, yielding 108 tissue samples from 40 *Oryza* accessions (34 roots, 40 shoots and 34 leaves). In the 2019 season, we focused on root endophytes and collected 124 root samples from 59 accessions spanning all major *Oryza* lineages. Sampling represented distinct evolutionary groups, including early-diverging species (12 samples from *O. brachyantha* and *O. australiensis*), medium-diverging species (86 samples from CC, BBCC and CCDD genome groups) and late-diverging AA-genome species (26 samples from *O. longistaminata*, *O. rufipogon* and *O. sativa* subsp. *indica*). Sampling also captured contrasts in ploidy (80 diploid and 44 allotetraploid samples) and life-history strategy (71 annual and 53 perennial samples).

All harvested tissues were processed using a standardized endophyte-enrichment workflow. Roots were first shaken to remove loosely adhering soil and then thoroughly washed with sterile water to eliminate any remaining soil particles. All tissues (roots, shoots, and leaves) were then surface-sterilized by sequential immersion in 70% ethanol (1 min) followed by 0.3% sodium hypochlorite with 0.01% Tween 20 (15 min), and finally rinsed three times with sterile water [1, 2]. Samples were stored at −80°C until DNA extraction.

2. DNA extraction and 16S rRNA and ITS gene sequencing

Genomic DNA was extracted from *Oryza* tissue samples using the E.Z.N.A. HP Plant DNA Kit (Omega Bio-tek, Inc) following the manufacturer's protocol. DNA quality and concentration were assessed by 1.0% agarose gel electrophoresis and a NanoDrop 2000 spectrophotometer (Thermo Scientific, USA). Extracted DNA was stored at -80 °C until further use.

For bacterial community profiling, the V4 hypervariable region of the 16S rRNA gene was amplified using primers 515F (5′-GTGCCAGCMGCCGCGGTAA-3′) and 806R (5′-GGACTACHVGGGTWTCTAAT-3′) [3]. For fungal community profiling, the ITS1 region was amplified using primers ITS1F (5′-CTTGGTCATTTAGAGGAAGTAA-3′) and ITS2 (5′-GCTGCGTTCTTCATCGATGC-3′) [4]. PCR reactions (15 μL) contained Phusion® High-Fidelity PCR Master Mix (New England Biolabs), 2 μM of each primer, and approximately 10 ng template DNA. Thermal cycling for both 16S rRNA and ITS amplification consisted of initial denaturation at 98 °C for 1 min, followed by 30 cycles of 98 °C for 10 s, 50 °C for 30 s, 72 °C for 30 s, with a final extension at 72 °C for 5 min. PCR products were verified on 2% agarose gels with SYBR Green loading buffer, pooled at equal concentrations, and purified using the Qiagen Gel Extraction Kit (Qiagen, Germany). Purified amplicons were quantified using Qubit 4.0 (Thermo Fisher Scientific, USA). Sequencing libraries were prepared with the TruSeq® DNA PCR-Free Sample Preparation Kit (Illumina, USA) according to the manufacturer's instructions, and library quality was assessed on a Qubit 2.0 Fluorometer and an Agilent Bioanalyzer 2100 system. Libraries were sequenced on the Illumina NovaSeq platform (Illumina, USA) to generate paired-end reads. Raw sequences have been deposited in the NCBI Sequence Read Archive (SRA).

3. 16S rRNA and ITS gene sequences processing

Analyses were performed using the clean, quality-filtered FASTQ files provided by Majorbio Bio-Pharm Technology Co., Ltd., in which raw sequencing reads had been pre-processed to remove low-quality sequences, merge paired-end reads, and demultiplex samples according to barcodes and primer sequences. The 16S rRNA and ITS gene sequences were processed using QIIME2 (v.2019.7) (https://qiime2.org/) and DADA2 [5].

For the 2015 paired-end datasets (16S rRNA V4 and ITS1), sequences were denoised using DADA2 in paired-end mode (qiime dada2 denoise-paired), with 20 bp trimmed from the 5′ end of forward and reverse reads (--p-trim-left-f 20 --p-trim-left-r 20) and truncated at 200 bp (forward) and 150 bp (reverse) (--p-trunc-len-f 200 --p-trunc-len-r 150) to remove low-quality tails while preserving sufficient overlap for merging. For the 2019 single-end datasets (16S rRNA V4 and ITS1), DADA2 was run in single-end mode (qiime dada2 denoise-single) with 30 bp trimmed from the 5′ end (--p-trim-left 30) and truncated at 240 bp (--p-trunc-len 240). Chimeric sequences were removed using the consensus method implemented in DADA2. Sequence quality and length distributions were inspected in QIIME2 (demux.qzv) prior to trimming and truncation. Within each sampling year, 16S rRNA and ITS sequences were processed with the same trimming parameters, as their per-base quality profiles were similar. The quality of the Illumina reads was additionally checked with FastQC (v.0.11.5). Taxonomic assignment of bacterial ASVs was performed using the RDP 2021.4 classifier with QIIME2's classify-sklearn method, and fungal ASVs were assigned using the UNITE (UNITE_ver7_dynamic, Jan 2017) database. ASVs assigned to chloroplasts or mitochondria were removed from all datasets. To standardize sequencing depth for downstream α- and β-diversity analyses, ASV counts were normalized in R by scaling to 100,000 sequences per sample (relative abundance × 100,000).

Principal coordinates analysis (PCoA) was performed using the Bray-Curtis distance metric in the 'vegan' R package (v2.6.4). A maximum-likelihood phylogenetic tree of significant ASVs was constructed with IQ-TREE (v2.0.3) and visualized using iTOL (https://itol.embl.de/). Potential functional taxa, including methanotrophs and nitrogen-fixers, were identified via Linear Discriminant Analysis Effect Size (LEfSe, v1.0.8) analysis (LDA score > 2.0, $p < 0.05$), with results visualized using custom R scripts. The machine learning framework employed was based on the 'randomForest' R package (v4.7.1).

Co-occurrence network was conducted using the 'igraph' R package (v1.2.6). The inferred correlations were restricted to those having abs(cor.r) > 0.6 and $p < 0.05$. We adopted criteria used in previous studies and identified module hubs ($Zi \geq 2.5$, $Pi < 0.62$), connectors ($Zi < 2.5$, $Pi \geq 0.62$) and network hubs ($Zi \geq 2.5$, $Pi \geq 0.62$).

Faith's phylogenetic diversity (Faith's PD) and the mean pairwise phylogenetic distance (MPD) were calculated for bacterial (16S rRNA) and fungal (ITS) communities using the picante R package [6]. A de novo phylogenetic tree was generated from 16S rRNA ASVs using QIIME 2 (qiime phylogeny align-to-tree-mafft-fasttree) and rooted with qiime phylogeny midpoint-root. MPD and Faith's PD were then calculated using the pruned phylogenetic tree aligned to the ASV abundance table. Standardized effect sizes (SES) for MPD were computed with the null model "taxa.labels" and 999 randomizations, allowing assessment of phylogenetic clustering or overdispersion.

Because ITS is a high-entropy, non-coding locus with limited phylogenetic signal across divergent fungal clades, phylogenetically aware metrics derived from ITS trees (e.g., Faith's PD, MPD) must be interpreted with caution. These ITS-based metrics are suitable for capturing *relative* patterns within a restricted taxonomic or ecological dataset, but they do not provide accurate estimates of deep evolutionary distance. To complement phylogenetic metrics, an alignment-free, k-mer–based MPD-like metric was computed following Bokulich [7]. ASV sequences were decomposed into overlapping 16-mers using q2-kmerizer (QIIME 2 v2024.10). TF–IDF weighting and L2 normalization were applied, low-frequency k-mers (<10 samples) were removed, and feature counts were capped (16S rRNA: 5,000 k-mers, ITS: 10,000 k-mers). For each sample, abundance-weighted pairwise cosine distances among ASVs were calculated, and their mean defined the k-mer MPD-like metric. Importantly, this metric reflects sequence composition similarity rather than true evolutionary relatedness and serves as a complementary, tree-independent validation.

To assess the relative contributions of determinism and stochasticity processes in microbiome assembly, we calculated the beta Nearest Taxon Index (βNTI) using the null model with 999 randomizations, considering $|\beta NTI| \geq 2$ as evidence of deterministic assembly and $|\beta NTI| < 2$ as stochastic. Additionally, a neutral community model (NCM) was used to assess the relationship between ASV detection frequency and relative abundance across the metacommunity, with 95% confidence intervals determined via 1,000 bootstrap replicates. Data visualization and statistical analyses were performed in R (v.4.1.3).

4. Quantification of host-microbiota phylogenetic congruence

To investigate how plant evolution shapes root microbial communities, we quantified phylogenetic signal using Blomberg's K and Pagel's λ statistics for each simulated trait with the phytools R package (v0.7.90).

Cophylogenetic relationships between hosts and their root microbiota were evaluated using a combination of hierarchical clustering and phylogenetic tree comparisons. Microbiome dendrograms were constructed by hierarchical clustering of Bray-Curtis distance using the hclust() function in R (v.4.1.3). Chloroplast genome sequences for 13 *Oryza* species were obtained from NCBI GenBank (accession numbers KF359901–KF359922) [8] to generate the host phylogeny.

Topological congruence between host and microbiota trees was quantified using a custom Python script from the Seth R. Bordenstein lab and the TreeCmp (v1.0) program [9, 10]. The topologies of both trees were constructed, and the normalized Robinson–Foulds score and normalized matching cluster score were calculated as the number of differences between the two topologies divided by the total possible congruency score for the two trees. Next, 100,000 random trees were constructed with the same number of leaf nodes, and each was compared to the host phylogeny. The number of trees which had an equivalent or better score than the actual microbiota dendrogram were used to calculate the significance of observing that topology under stochastic assembly. Normalized results of both statistics have been provided to facilitate comparison. Matching cluster and Robinson–Foulds p-values were determined by the probability of 100,000 randomized bifurcating dendrogram topologies yielding equivalent or more congruent phylosymbiotic patterns than the microbiota dendrogram**.**

Additionally, topological congruence was evaluated using the Clustering Information Distance (CID) metric with the TreeDist R package (v2.11.1) in R (v.4.3.1), with statistical significance determined by 9,999 permutations [11]. Both normalized metrics and p-values were reported to allow quantitative comparison across metrics and sampling years.

5. Ancestral state reconstruction

To reconstruct the ancestral states of the root microbiome, a presence/absence matrix of potential nitrogen-fixing taxa was mapped against the *Oryza* phylogeny using Count software (v.10.04) [12]. Taxa with <1% relative abundance and <20% prevalence were considered absent,

and gains/losses across host lineages were inferred by asymmetrical Wagner parsimony with gain and loss penalties of 1.5 and 1, respectively.

6. Bacteria isolation and genome sequencing

Bacteria strains were isolated from 2019 field-collected *Oryza* roots and their rhizosphere soil using multiple culture media (Table S11). Homogenized plant tissues and rhizosphere soil were centrifuged for 15 min, and the supernatants were empirically diluted, distributed, and cultivated in microtiter plates in different media at 30 °C (Table S11). Genomic DNA from each bacterial isolation was extracted using the E.Z.N.A. Bacterial DNA kit (Omega Bio-tek, Inc). Bacterial identities were initially determined by sequencing the 16S rRNA gene using the 27F/1492R primer pair on the Sanger sequencing platform, followed by taxonomic annotation with NCBI BLAST and the Ribosomal Database Project (RDP).

For all bacterial strains selected for the synthetic community (SynCom) and subsequent pot experiments, whole-genome sequencing was performed on Illumina platforms (Novogene Co., Ltd., Beijing, China). Genome assembly was conducted with Unicycler (v0.4.7) [13], and gene annotation was carried out using Prokka (v1.14.6) [14]. Genome quality was assessed using CheckM (v1.2.3) to evaluate completeness and contamination, and taxonomy was assigned with GTDB-Tk (v2.4.0) to ensure accurate phylogenetic placement [15, 16].

7. Constructing Synthetic Communities (SynCom)

Synthetic communities were constructed using a dual-selection strategy aimed at disentangling the effects of (i) ecologically conserved core taxa and (ii) functionally specialized nitrogen-fixing bacteria. The Core SynCom (19 strains) was derived from representative bacterial isolates corresponding to core Amplicon Sequence Variants (ASVs), defined as those that were highly abundant (top 10% by mean relative abundance) and ubiquitous prevalent (present in >50% of wild *Oryza* accessions) (Table S10) [17]. Nineteen isolates spanning these core taxa were assembled at equal abundance to capture the ecologically conserved structure of the wild rice root microbiome.

To separately evaluate functional contributions, we selected four putative nitrogen-fixing strains—*Phaeospirillum* sp. (Ps), *Rhizobium* sp. (Rs), *Geobacter sulfurreducens* (Gs), and

Azorhizobium caulinodans (Ac)—based on significant phylogenetic enrichment of their corresponding genera in early- and medium-diverging *Oryza* species (Fig. 4D). These isolates were used as single-strain inoculants in parallel with the Core SynCom.

This experimental design generated six treatments: a non-inoculated control, the 19-strain Core SynCom, and four individual nitrogen-fixing strain treatments. Together, these conditions enabled direct comparison between microbiome-level synergistic effects and the functional contributions of individual beneficial taxa.

8. Pot Experiment (Growth-Promotion Assay)

For pot experiments, rice plants (*Oryza sativa* L. subsp. *japonica* cv. Zhonghua 11) were cultivated in plastic pots filled with 20 kg of red clay soil supplemented with defined nitrogen, phosphorus, and potassium fertilizers (application rates in Table S12). Rice seeds were surface-sterilized following a standardized multistep protocol. Seeds were first rinsed with sterile water for 30 s and then washed in 70% ethanol for 30 s. Seeds were subsequently immersed in 3% sodium hypochlorite solution supplemented with 0.01% Tween-20 for 15 min, with gentle agitation every 5 min. After sterilization, seeds were washed rinsed five times with sterile water (5 min each). Finally, excess surface moisture was removed using sterile filter paper. Seeds were allowed to germinate. When the rice grows to three leaves and one heart, they are transplanted to moist red soil that has been fertilized in advance. Five plants are planted in each pot, and four replicates are made for each different treatment. Watering is carried out according to agricultural requirements during the rice development period.

After isolation, all bacterial strains were subcultured and maintained on a simplified set of media to ensure consistent growth. Most strains were maintained on LB or TSB media, while *Streptomyces* (MJ8) was maintained on R2A and *Herbaspirillum* (Hs) on Ashby medium (Table S14). Although initial isolation used multiple media types (Table S11), all experimental culturing was performed exclusively on the maintenance media. Bacterial cells were harvested by centrifugation and resuspended in sterile PBS. Single-strain cultures were adjusted to an OD_{600} of ~0.5, and SynCom inocula were prepared as equimolar mixtures of component strains to a final OD_{600} of ~0.5.

Bacterial suspensions were applied to the rhizosphere soil immediately before seedling transplantation, with each plant receiving a total of 2 mL of bacterial suspension (or PBS for the control treatment). Plants and rhizosphere soils were harvested at the mature stage for downstream analyses.

9. Serum Bottle SynCom Culture Experiment

The SynComs were co-cultured in OS media using serum bottles by adding different concentrations of NH_4NO_3 (ON: 0 mM, LN: 0.1 mM, SN: 1.0 mM) for 48h at 30 °C (Table S11). Each condition had 4 biological replicates. Biomass was harvested for metagenomic sequencing.

10. Metagenome sequencing and analysis

DNA extracted from rhizosphere and SynCom co-culture samples was sequenced on Illumina NovaSeq (pot experiment) and BGISEQ-T7 (SynCom serum bottle experiment) platforms. Raw sequencing reads were preprocessed by the sequencing provider to remove adapters, low-quality sequences, and potential contaminants. Downstream assembly and analysis were performed starting from these clean reads. Clean reads were assembled with MEGAHIT (v1.2.9, -m 0.9) to generate contigs [18]. Open reading frames (ORFs) were predicted using Prodigal and annotated with Prokka (v1.11) [14]. Redundant genes were clustered using CD-HIT (v4.8.1, cd-hit-est -c 0.95 -aS 0.9) to generate a non-redundant gene catalog, and gene abundance was quantified with Salmon (v1.10.3) [19]. Functional annotation of predicted ORFs was performed against the EggNOG database (v5.0.2) using Diamond (v2.1.6) [20].

Genome binning was performed using the MetaWRAP pipeline (v1.3.2), integrating three algorithms (CONCOCT, MaxBin2, MetaBAT2) to generate initial bins [21, 22]. The bins were refined using MetaWRAP bin_refinement (-c 50 -x 10) and subsequently dereplicated with dRep (v3.5.0, -sa 0.95 -nc 0.3 -comp 50 -con 10) to remove redundancy. Bin quality was assessed with CheckM (v1.2.3), and only high-quality MAGs with ≥90% completeness and ≤5% contamination were retained [15]. Taxonomic assignment of MAGs was performed using GTDB-Tk (v2.4.0) based on the GTDB reference database [16]. Coverage-based abundance estimation of MAGs across samples was calculated using CoverM (v0.7.0), enabling downstream quantitative analyses of MAG distribution in pot and SynCom experiments [23]. This workflow ensures the recovery of high-quality, non-redundant MAGs with confident functional and taxonomic annotation,

supporting ecological and functional inference of microbial communities in rice rhizosphere and synthetic community experiments.

11. In Silico Assessment of Plant Pathogenic Potential

To evaluate the potential plant pathogenicity of bacterial isolates selected for the Synthetic Community (SynCom), protein sequences from each strain were queried against the Pathogen-Host Interactions database (PHI-base, v4.17) using BLASTP (v2.13.0+) [24, 25]. PHI-base is a manually curated repository of experimentally verified genes that affect pathogen–host interactions. High-stringency BLASTP parameters were applied (E-value ≤ 1e-5, minimum query coverage 50%), and only hits associated with plant pathogens were retained. Homologous genes were identified with a sequence identity threshold of ≥30%, and a subset of high-similarity genes (≥70% identity) was further analyzed for functional annotation based on PHI-base classifications (e.g., effector, toxin, regulatory protein) and in planta phenotypes (e.g., reduced virulence, loss of pathogenicity). Strains were assigned a risk category as follows: low risk, if no high-similarity core virulence factors were detected or only regulatory genes were present; medium risk, if isolated virulence-associated genes were present but complete pathogenic systems (e.g., intact secretion system gene clusters) were absent. No strains were classified as high risk.

12. Experimental Validation of Pathogenicity

To assess the pathogenic potential of selected SynCom members toward rice, we performed a soil-based assay under controlled conditions. Three strains—*Pseudomonas* sp. WN26, *Burkholderia* sp. CD4, and *Burkholderia* sp. W1—were tested alongside two non-pathogenic control strains (*Rhizobium* sp. W17 and *Herbaspirillum* sp. HS). Rice seeds were surface-sterilized, germinated for two days, and then dipped in bacterial suspensions (OD_{600} = 0.2) for 10 min. Seeds treated with sterile phosphate-buffered saline (PBS) served as the uninoculated control. Treated seedlings were transplanted into autoclaved soil (121 °C for 30 min on three consecutive days) and grown under both low-nitrogen (LN) and standard-nitrogen (SN) conditions in a greenhouse for six days. Plants were observed daily for disease symptoms such as chlorosis, necrosis, water-soaked lesions, or developmental arrest.

Statistical Analysis

All statistical analyses were performed in R (v4.1.3). Differences in microbial community composition across host species, ploidy levels, and divergence times were assessed using Permutational Multivariate Analysis of Variance (PERMANOVA) and pairwise PERMANOVA implemented via the adonis () and pairwise.adonis () functions from the vegan R package (v2.6.4), respectively. To identify Amplicon Sequence Variants (ASVs) responsive to *Oryza* evolutionary history, differential abundance analyses were performed using DESeq2 (v1.32.0) with Benjamini-Hochberg adjusted p-values (padj < 0.05). In parallel, generalized linear models (GLMs) with negative binomial regression were fitted using the mvabund R package (v4.2.1) to quantify ASV responses across host divergence times and ploidy levels. Correlations between microbial abundances and host traits were computed using Pearson correlation. Non-parametric Wilcoxon tests and parametric t-tests were applied as appropriate for group comparisons, with exact p-values reported in the text. This integrated approach allowed us to combine community-level multivariate analyses with ASV-specific and trait-specific tests to robustly characterize host-microbiome associations.

Supplementary References

1. Xie J, Wang X, Xu J, Xie H, Cai Y, Liu Y, Ding X. Strategies and Structure Feature of the Aboveground and Belowground Microbial Community Respond to Drought in Wild Rice (Oryza longistaminata). Rice (N Y). 2021;14l(1):79l.
2. Peng X, Xie J, Li W, Xie H, Cai Y, Ding X. Comparison of wild rice (Oryza longistaminata) tissues identifies rhizome-specific bacterial and archaeal endophytic microbiomes communities and network structures. PLoS One. 2021;16l(2):e0246687l.
3. Caporaso JG, Lauber CL, Walters WA, Berg-Lyons D, Lozupone CA, Turnbaugh PJ, Fierer N, Knight R. Global patterns of 16S rRNA diversity at a depth of millions of sequences per sample. Proc Natl Acad Sci U S A. 2011;108 Suppl 1l(Suppl 1):4516-22l.
4. Walters W, Hyde ER, Berg-Lyons D, Ackermann G, Humphrey G, Parada A, Gilbert JA, Jansson JK, Caporaso JG, Fuhrman JA, Apprill A, Knight R. Improved Bacterial 16S rRNA Gene (V4 and V4-5) and Fungal Internal Transcribed Spacer Marker Gene Primers for Microbial Community Surveys. mSystems. 2016;1l(1):
5. Bolyen E, Rideout JR, Dillon MR, Bokulich NA, Abnet CC, Al-Ghalith GA, Alexander H, Alm EJ, Arumugam M, Asnicar F, Bai Y, Bisanz JE, Bittinger K, Brejnrod A, Brislawn CJ, Brown CT, Callahan BJ, Caraballo-Rodriguez AM, Chase J, Cope EK, Da Silva R, Diener C, Dorrestein PC, Douglas GM, Durall DM, Duvallet C, Edwardson CF, Ernst M, Estaki M, Fouquier J, Gauglitz JM, Gibbons SM, Gibson DL, Gonzalez A, Gorlick K, Guo J, Hillmann B, Holmes S, Holste H, Huttenhower C, Huttley GA, Janssen S, Jarmusch AK, Jiang L, Kaehler BD, Kang KB, Keefe CR, Keim P, Kelley ST, Knights D, Koester I, Kosciolek T, Kreps J, Langille MGI, Lee J, Ley R, Liu YX, Loftfield E, Lozupone C, Maher M, Marotz C, Martin BD, McDonald D, McIver LJ, Melnik AV, Metcalf JL, Morgan SC,

Morton JT, Naimey AT, Navas-Molina JA, Nothias LF, Orchanian SB, Pearson T, Peoples SL, Petras D, Preuss ML, Pruesse E, Rasmussen LB, Rivers A, Robeson MS, 2nd, Rosenthal P, Segata N, Shaffer M, Shiffer A, Sinha R, Song SJ, Spear JR, Swafford AD, Thompson LR, Torres PJ, Trinh P, Tripathi A, Turnbaugh PJ, Ul-Hasan S, van der Hooft JJJ, Vargas F, Vazquez-Baeza Y, Vogtmann E, von Hippel M, Walters W, Wan Y, Wang M, Warren J, Weber KC, Williamson CHD, Willis AD, Xu ZZ, Zaneveld JR, Zhang Y, Zhu Q, Knight R, Caporaso JG. Reproducible, interactive, scalable and extensible microbiome data science using QIIME 2. Nat Biotechnol. 2019;37l(8):852-7l.

6. Kembel SW, Cowan PD, Helmus MR, Cornwell WK, Morlon H, Ackerly DD, Blomberg SP, Webb CO. Picante: R tools for integrating phylogenies and ecology. Bioinformatics. 2010;26l(11):1463-4l.

7. Bokulich NA. Integrating sequence composition information into microbial diversity analyses with k-mer frequency counting. mSystems. 2025;10l(3):e0155024l.

8. Gao LZ, Liu YL, Zhang D, Li W, Gao J, Liu Y, Li K, Shi C, Zhao Y, Zhao YJ, Jiao JY, Mao SY, Gao CW, Eichler EE. Evolution of Oryza chloroplast genomes promoted adaptation to diverse ecological habitats. Commun Biol. 2019;2l(278l.

9. Lim SJ, Bordenstein SR. An introduction to phylosymbiosis. Proc Biol Sci. 2020;287l(1922):20192900l.

10. Brooks AW, Kohl KD, Brucker RM, van Opstal EJ, Bordenstein SR. Phylosymbiosis: Relationships and Functional Effects of Microbial Communities across Host Evolutionary History. PLoS Biol. 2016;14l(11):e2000225l.

11. Smith MR. Information theoretic generalized Robinson-Foulds metrics for comparing phylogenetic trees. Bioinformatics. 2021;37l(14):2077-8l.

12. Csuros M. Count: evolutionary analysis of phylogenetic profiles with parsimony and likelihood. Bioinformatics. 2010;26l(15):1910-2l.

13. Wick RR, Judd LM, Gorrie CL, Holt KE. Unicycler: Resolving bacterial genome assemblies from short and long sequencing reads. PLoS Comput Biol. 2017;13l(6):e1005595l.

14. Seemann T. Prokka: rapid prokaryotic genome annotation. Bioinformatics. 2014;30l(14):2068-9l.

15. Parks DH, Imelfort M, Skennerton CT, Hugenholtz P, Tyson GW. CheckM: assessing the quality of microbial genomes recovered from isolates, single cells, and metagenomes. Genome Res. 2015;25l(7):1043-55l.

16. Chaumeil PA, Mussig AJ, Hugenholtz P, Parks DH. GTDB-Tk: a toolkit to classify genomes with the Genome Taxonomy Database. Bioinformatics. 2019;36l(6):1925-7l.

17. Jiao S, Qi J, Jin C, Liu Y, Wang Y, Pan H, Chen S, Liang C, Peng Z, Chen B, Qian X, Wei G. Core phylotypes enhance the resistance of soil microbiome to environmental changes to maintain multifunctionality in agricultural ecosystems. Glob Chang Biol. 2022;28l(22):6653-64l.

18. Li D, Liu CM, Luo R, Sadakane K, Lam TW. MEGAHIT: an ultra-fast single-node solution for large and complex metagenomics assembly via succinct de Bruijn graph. Bioinformatics. 2015;31l(10):1674-6l.

19. Li W, Godzik A. Cd-hit: a fast program for clustering and comparing large sets of protein or nucleotide sequences. Bioinformatics. 2006;22l(13):1658-9l.

20. Huerta-Cepas J, Szklarczyk D, Heller D, Hernandez-Plaza A, Forslund SK, Cook H, Mende DR, Letunic I, Rattei T, Jensen LJ, von Mering C, Bork P. eggNOG 5.0: a

hierarchical, functionally and phylogenetically annotated orthology resource based on 5090 organisms and 2502 viruses. Nucleic Acids Res. 2019;47l(D1):D309-D14l.

21. Uritskiy GV, DiRuggiero J, Taylor J. MetaWRAP-a flexible pipeline for genome-resolved metagenomic data analysis. Microbiome. 2018;6l(1):158l.

22. Bai D, Chen T, Xun J, Ma C, Luo H, Yang H, Cao C, Cao X, Cui J, Deng YP, Deng Z, Dong W, Dong W, Du J, Fang Q, Fang W, Fang Y, Fu F, Fu M, Fu YT, Gao H, Ge J, Gong Q, Gu L, Guo P, Guo Y, Hai T, Liu H, He J, He ZY, Hou H, Huang C, Ji S, Jiang C, Jiang GL, Jiang L, Jin LN, Kan Y, Kang D, Kou J, Lam KL, Li C, Li C, Li F, Li L, Li M, Li X, Li Y, Li ZT, Liang J, Lin Y, Liu C, Liu D, Liu F, Liu J, Liu T, Liu T, Liu X, Liu Y, Liu B, Liu M, Lou W, Luan Y, Luo Y, Lv H, Ma T, Mai Z, Mo J, Niu D, Pan Z, Qi H, Shi Z, Song C, Sun F, Sun Y, Tian S, Wan X, Wang G, Wang H, Wang H, Wang H, Wang J, Wang J, Wang K, Wang L, Wang SK, Wang X, Wang Y, Xiao Z, Xing H, Xu Y, Yan SY, Yang L, Yang S, Yang Y, Yao X, Yousuf S, Yu H, Lei Y, Yuan Z, Zeng M, Zhang C, Zhang C, Zhang H, Zhang J, Zhang N, Zhang T, Zhang YB, Zhang Y, Zhang Z, Zhou M, Zhou Y, Zhu C, Zhu L, Zhu Y, Zhu Z, Zou H, Zuo A, Dong W, Wen T, Chen S, Li G, Gao Y, Liu YX. EasyMetagenome: A user-friendly and flexible pipeline for shotgun metagenomic analysis in microbiome research. Imeta. 2025;4l(1):e70001l.

23. Aroney STN, Newell RJP, Nissen JN, Camargo AP, Tyson GW, Woodcroft BJ. CoverM: read alignment statistics for metagenomics. Bioinformatics. 2025;41l(4):

24. Urban M, Cuzick A, Seager J, Nonavinakere N, Sahoo J, Sahu P, Iyer VL, Khamari L, Martinez MC, Hammond-Kosack KE. PHI-base - the multi-species pathogen-host interaction database in 2025. Nucleic Acids Res. 2025;53l(D1):D826-D38l.

25. Camacho C, Coulouris G, Avagyan V, Ma N, Papadopoulos J, Bealer K, Madden TL. BLAST+: architecture and applications. BMC Bioinformatics. 2009;10l(421l.
