# Supplementary material for: Microbiome eco-evolution of cultivated and wild rice species across the genus Oryza and its importance in supporting rice growth
Source: Microbiome. 2026 Mar 6;14:83. doi: 10.1186/s40168-026-02359-z (PMC12973805; doi:10.1186/s40168-026-02359-z)
Supplement: Supplementary file 2 — Additional file 1. Supplementary figures. Figure S1 Alpha diversity varies across host compartments. Observed features indices of the roots, shoots, and leaves bacteria (A) and fungi (C). Chao1 indices of the roots, shoots, and leaves bacteria (B) and fungi (D). p values reflect the Wilcoxon test. Figure S2 The number of bacterial and fungal ASVs found exclusively in particular compartments. Venn diagrams illustrating bacterial (A) and fungal (B) ASVs shared among host compartments. Figure S3 The relative abundance of microbial taxa across host compartments at the genus level. Relative abundance of bacterial (A) and fungal (B) genera in the roots, shoots, and leaves. Figure S4 Inter-annual and host genetic effects on root microbiome composition. Principal coordinate analysis (PCoA) based on Bray–Curtis distances, performed on the subset of accessions (n = 10) sampled in both the 2015 and 2019 campaigns. (A) Samples colored by sampling year (2015 vs. 2019). (B) Samples colored by host evolutionary complex (Officinalis, Sativa, and unclassified groups). (C) Samples colored by host divergence time (early, medium, and late). Note: Because the 2015 and 2019 campaigns included largely non-overlapping accessions, only a limited number of accessions were sampled in both years. Figure S5 The relative abundance of bacterial taxa in the roots of 40 Oryza accessions. The relative abundance in the (A) phylum and (B) genus level of bacterial taxa. Figure S6 The relative abundance of fungal taxa in the roots of 40 Oryza accessions. The relative abundance in the (A) phylum and (B) genus level of fungal taxa. Figure S7 PCoAs of bacterial and fungal communities among different life cycle based on the Bray–Curtis distance matrix. Figure S8 PCoA of root microbiomes across host evolutionary scales. Principal coordinate analysis (PCoA) based on Bray–Curtis distances. (A) Differentiation between the Officinalis (non-AA genome) and Sativa (AA genome) complexes. (B) Differentiation within t [file 40168_2026_2359_MOESM1_ESM.pdf]

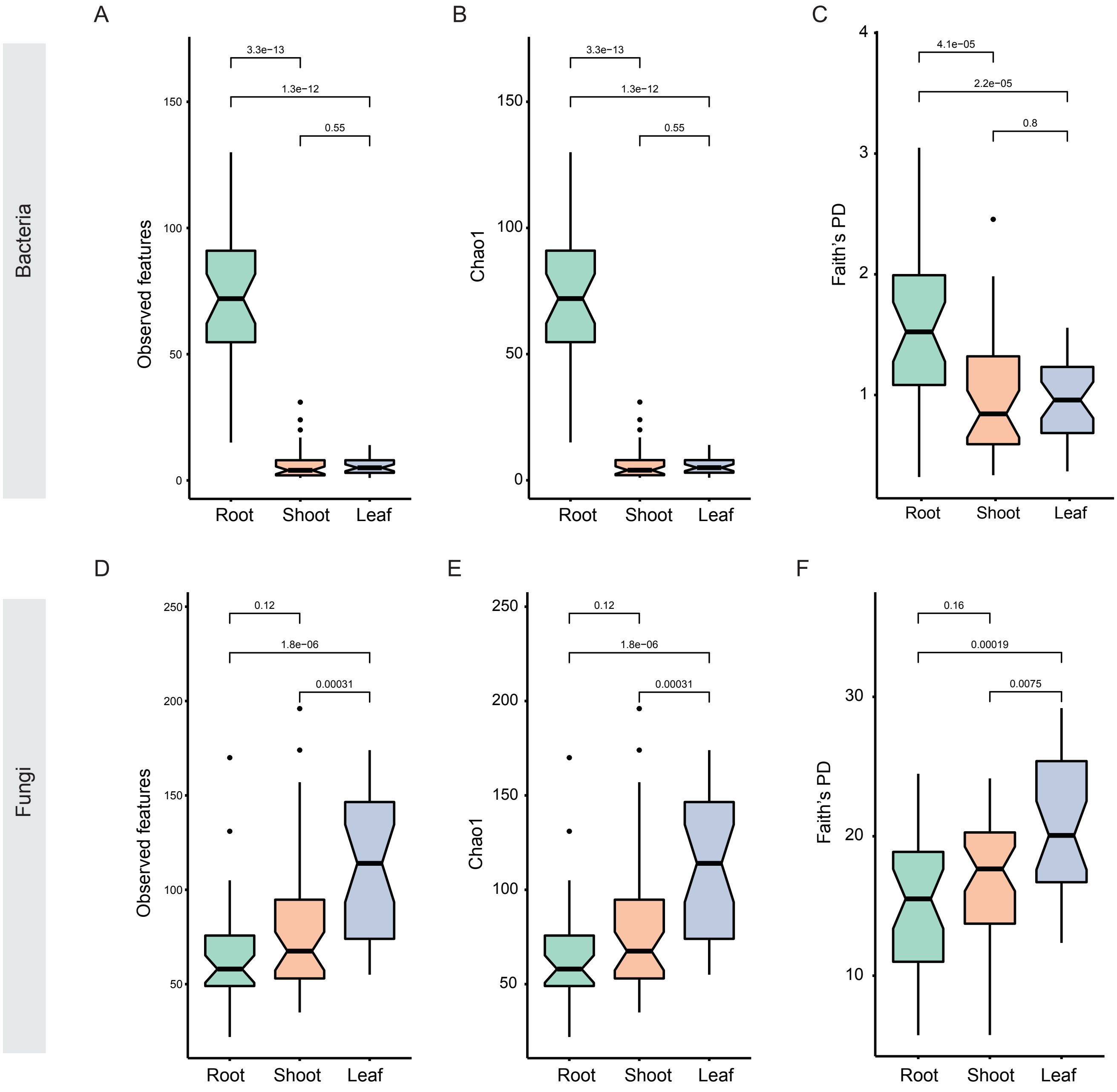

Fig. S1. Alpha diversity varies across host compartments.
Observed features indices of the roots, shoots, and leaves bacteria (A) and fungi (D).
Chao1 indices of the roots, shoots, and leaves bacteria (B) and fungi (E).
Faith's PD indices of the roots, shoots, and leaves bacteria (C) and fungi (F).
P-values reflect the Wilcoxon test.

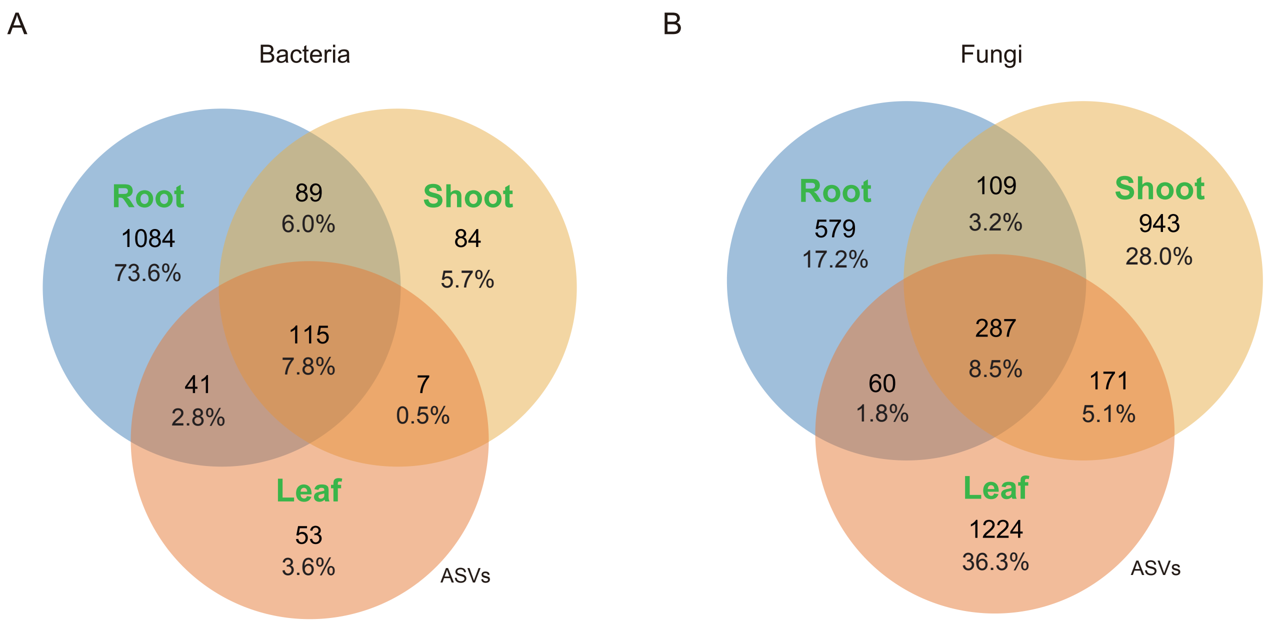

Fig. S2. The number of bacterial and fungal ASVs found exclusively in particular compartments. Venn diagrams illustrating bacterial (A) and fungal (B) ASVs shared among host compartments.

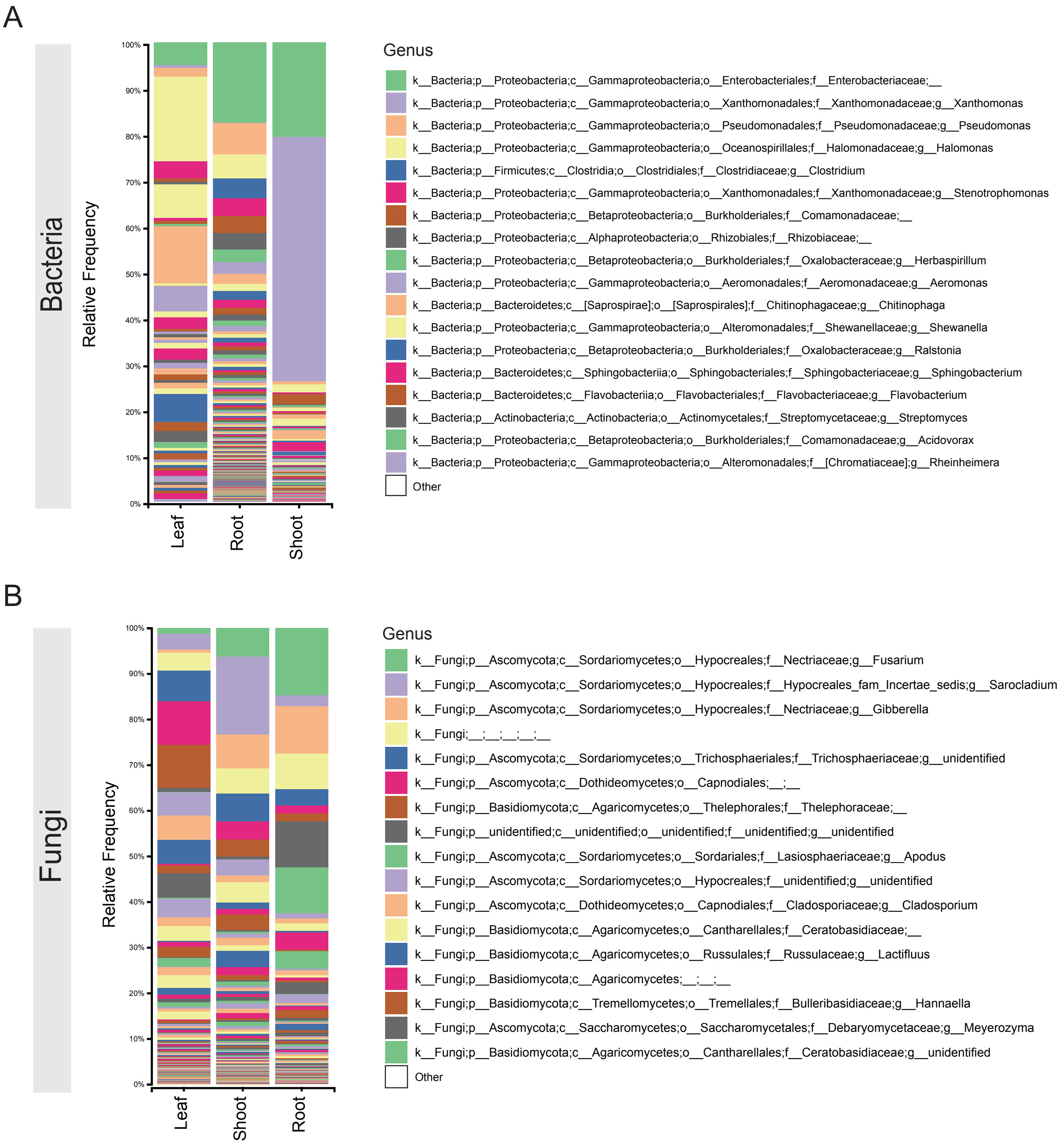

Fig. S3. The relative abundance of microbial taxa across host compartments at the genus level.
Relative abundance of bacterial (A) and fungal (B) genera in the roots, shoots, and leaves.

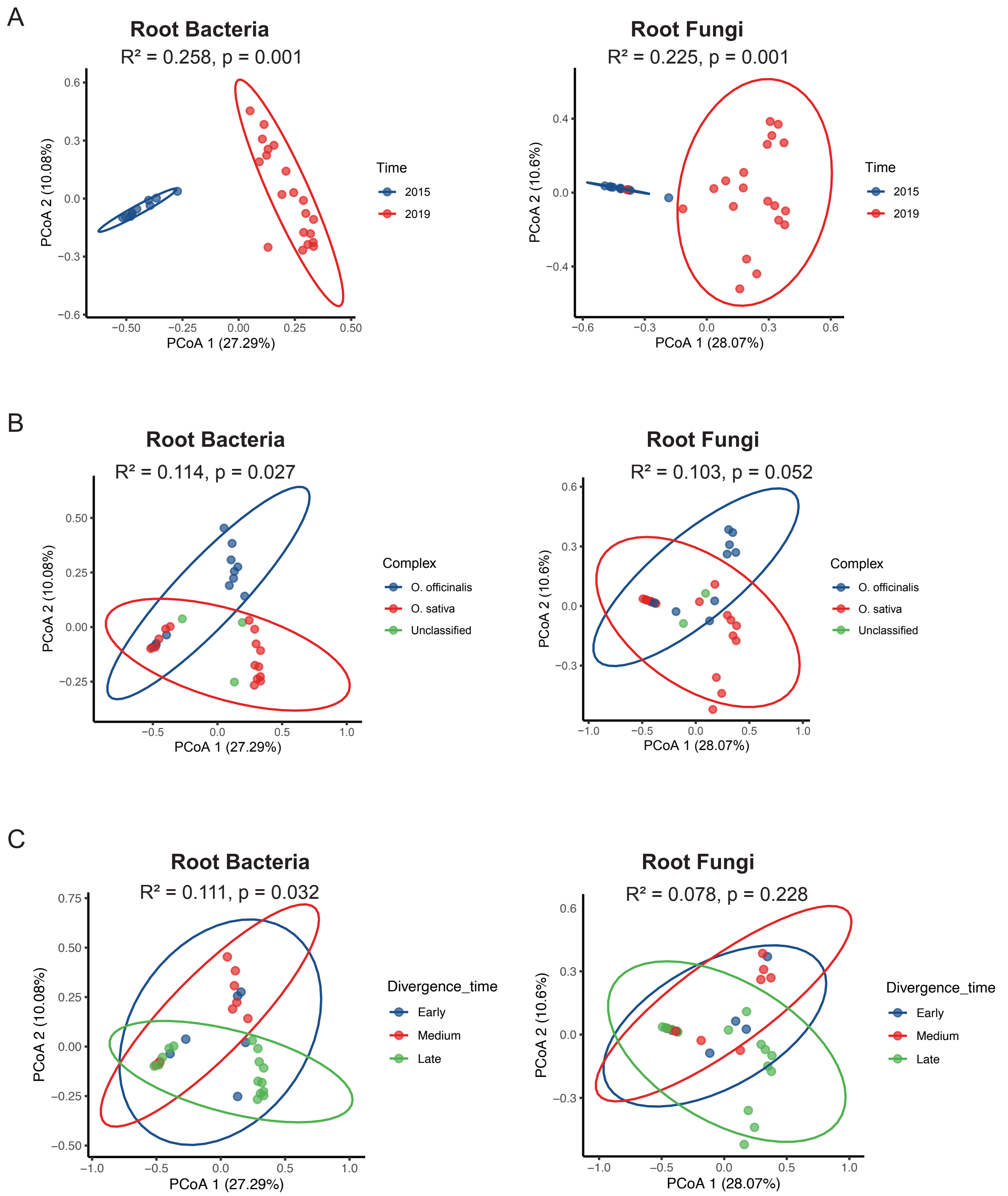

Figure S4 Inter-annual and host genetic effects on root microbiome composition.

Principal-coordinates analysis (PCoA) based on Bray–Curtis distances, performed on the subset of accessions (n = 10) sampled in both the 2015 and 2019 campaigns.

(A) Samples colored by sampling year (2015 vs. 2019).

(B) Samples colored by host evolutionary complex (Officinalis, Sativa, and unclassified groups).

(C) Samples colored by host divergence time (early, medium and late).

Note: Because the 2015 and 2019 campaigns included largely non-overlapping accessions, only a limited number of accessions were sampled in both years.

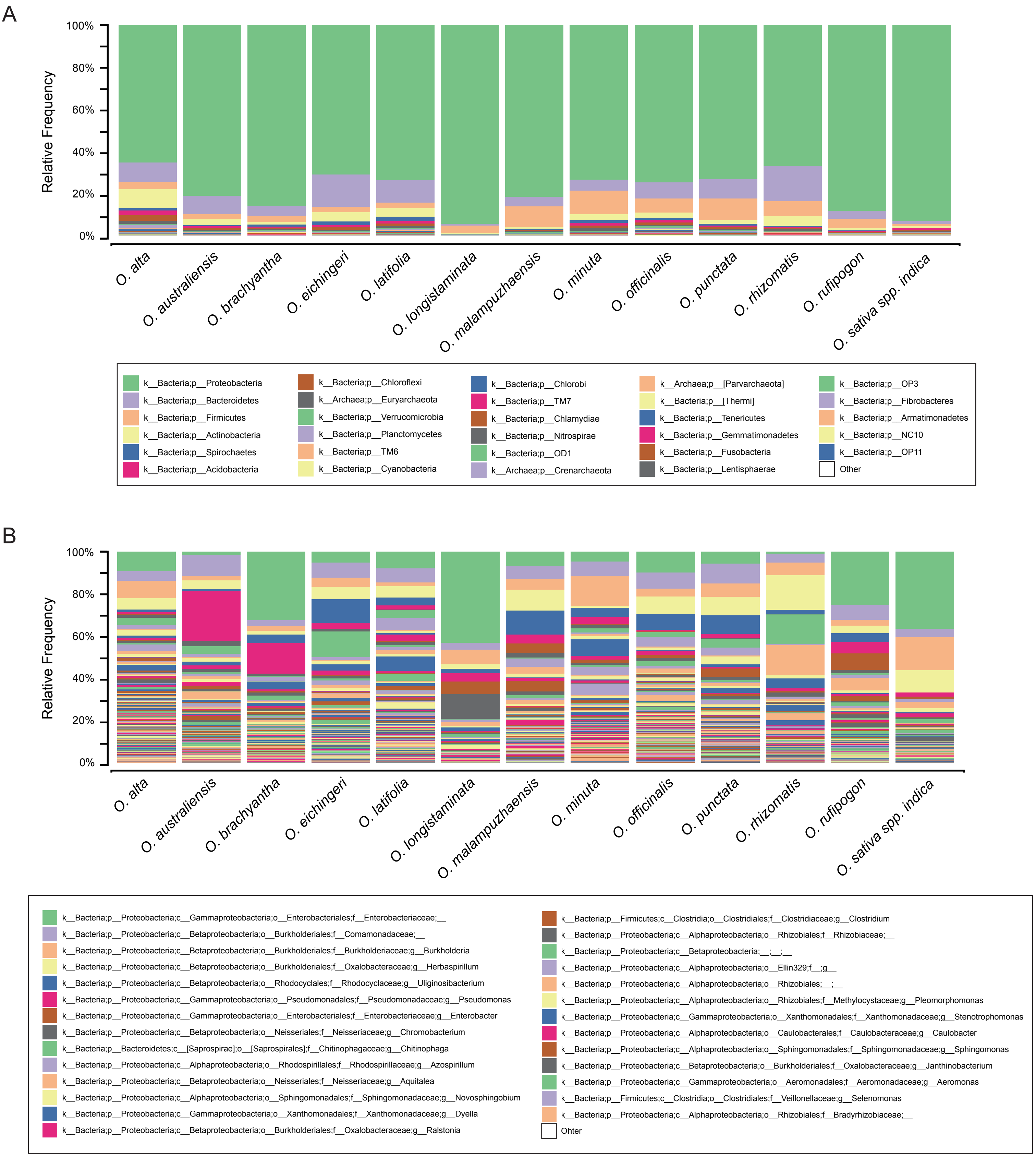

Fig. S5. The relative abundance of bacterial taxa in the roots of 40 *Oryza* accessions. The relative abundance in the (A) phylum and (B) genus level of bacterial taxa.

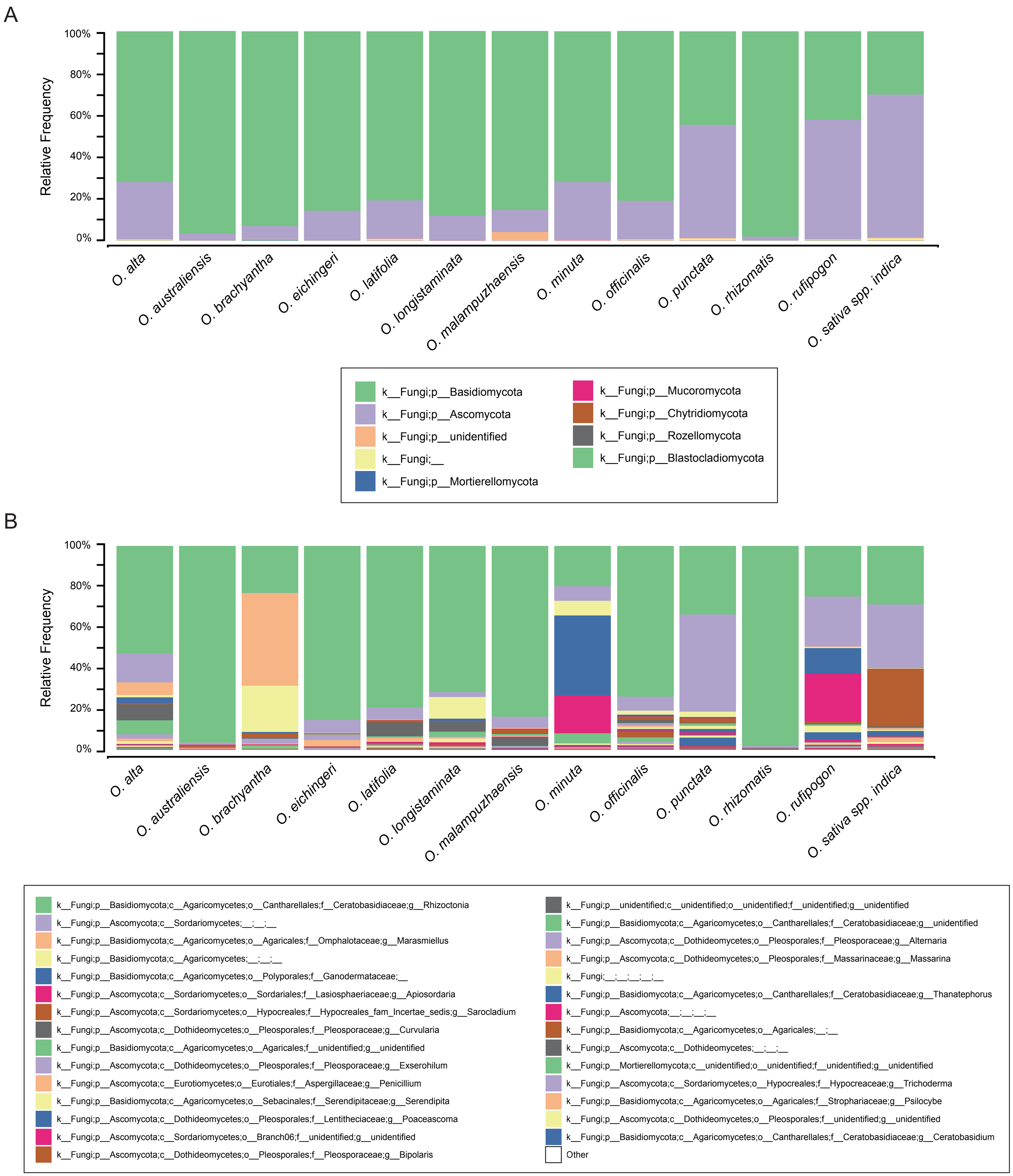

Fig. S6. The relative abundance of fungal taxa in the roots of 40 *Oryza* accessions. The relative abundance in the (A) phylum and (B) genus level of fungal taxa.

The relative abundance in the (A) phylum and (B) genus level of fungal taxa.

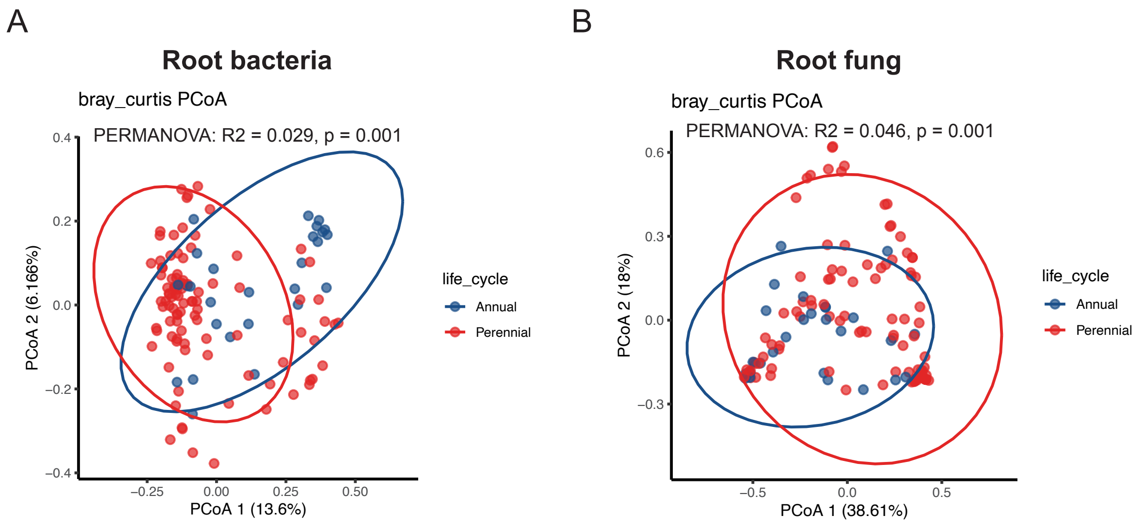

Figure S7 PCoAs of bacterial and fungal communities among different life cycle based on the Bray-Curtis distance matrix.

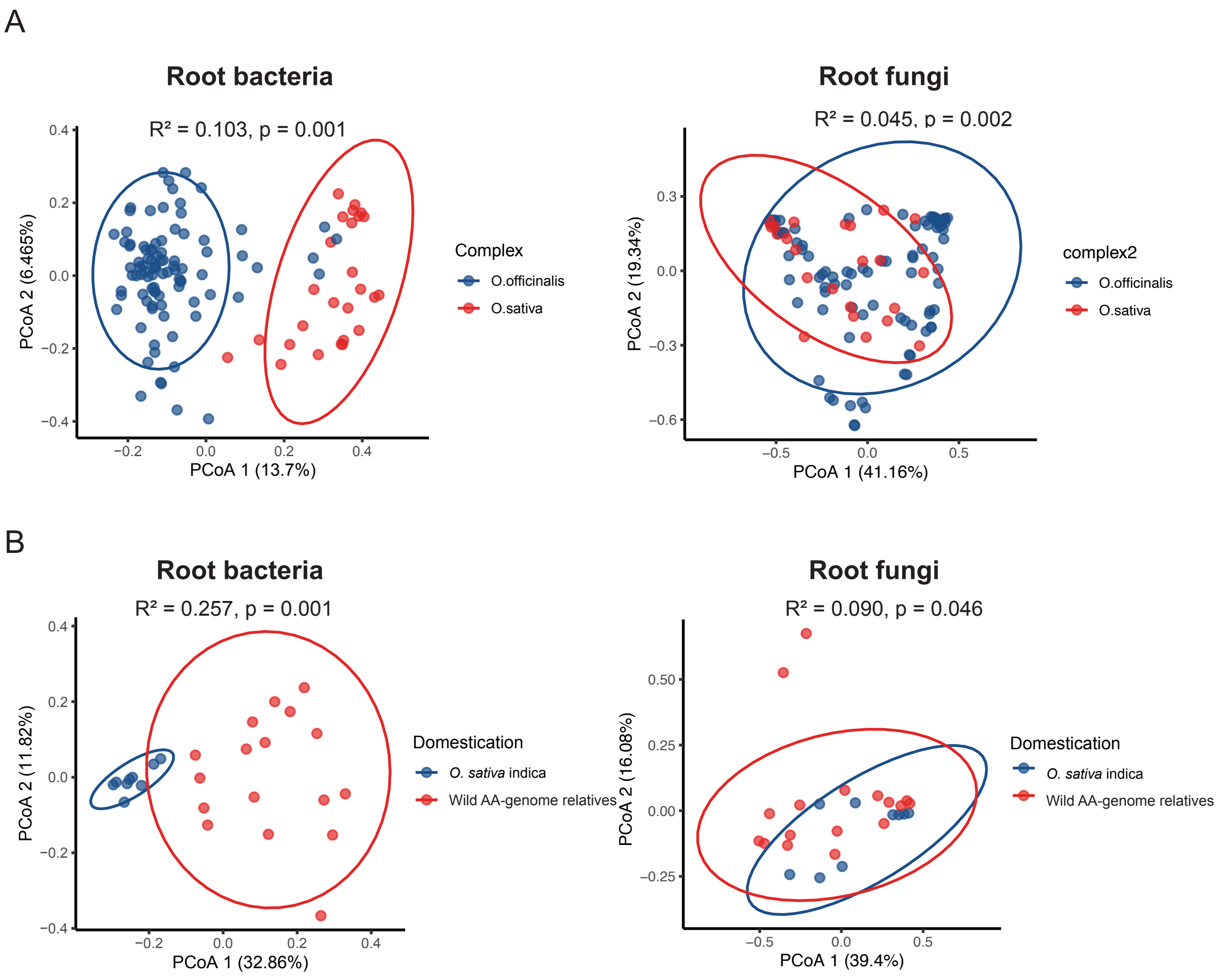

Figure S8 PCoA of root microbiomes across host evolutionary scales.

Principal-coordinates analysis (PCoA) based on Bray–Curtis distances. (A) Differentiation between the Officinalis (non-AA genome) and Sativa (AA genome) complexes.
(B) Differentiation within the AA-genome Sativa complex, comparing cultivated Oryza sativa ssp. indica with wild AA-genome relatives. Statistical significance (PERMANOVA) is indicated.

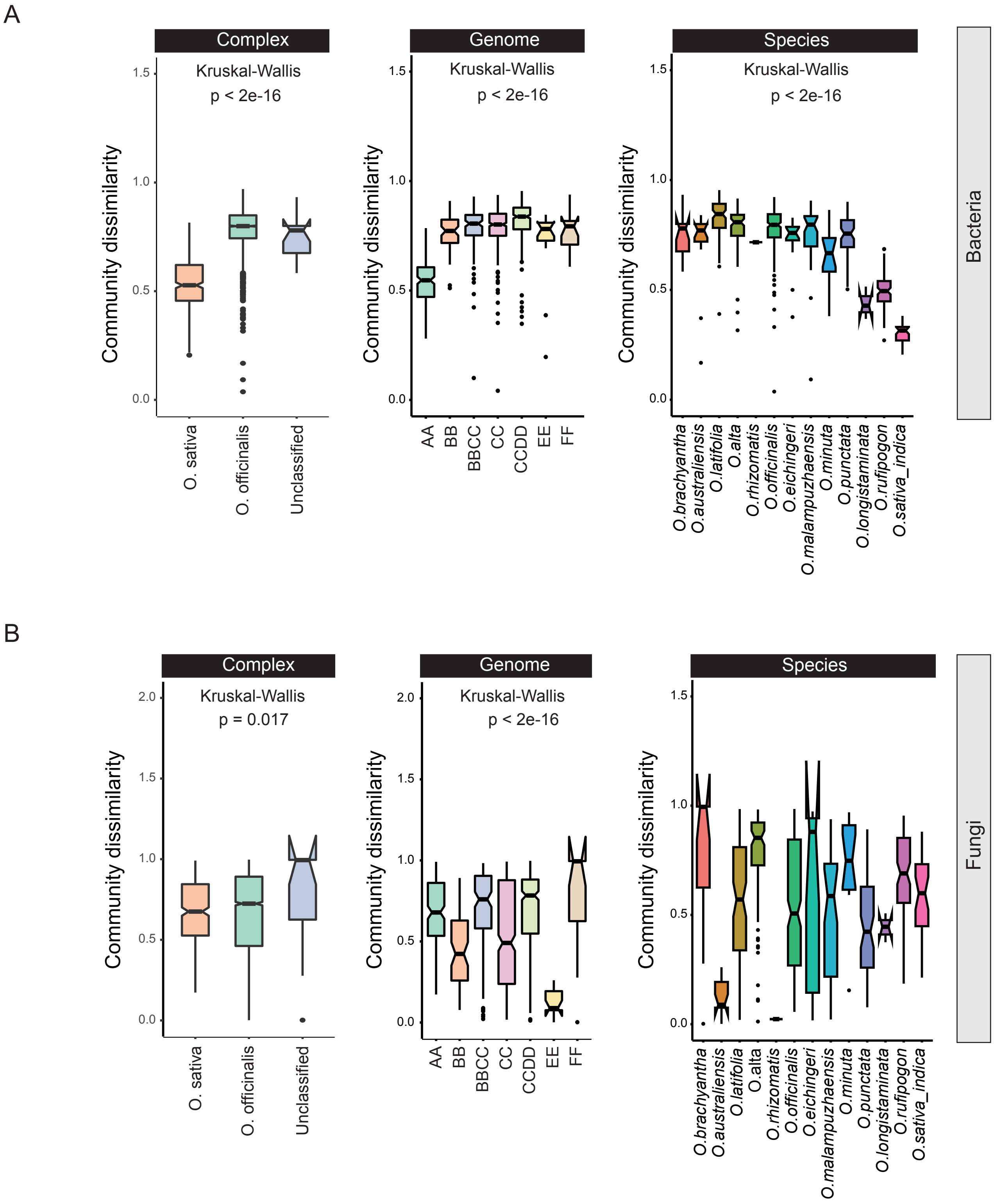

Figure S9 The Bray-Curtis dissimilarity of the root bacterial and fungal communities across the host genotype.

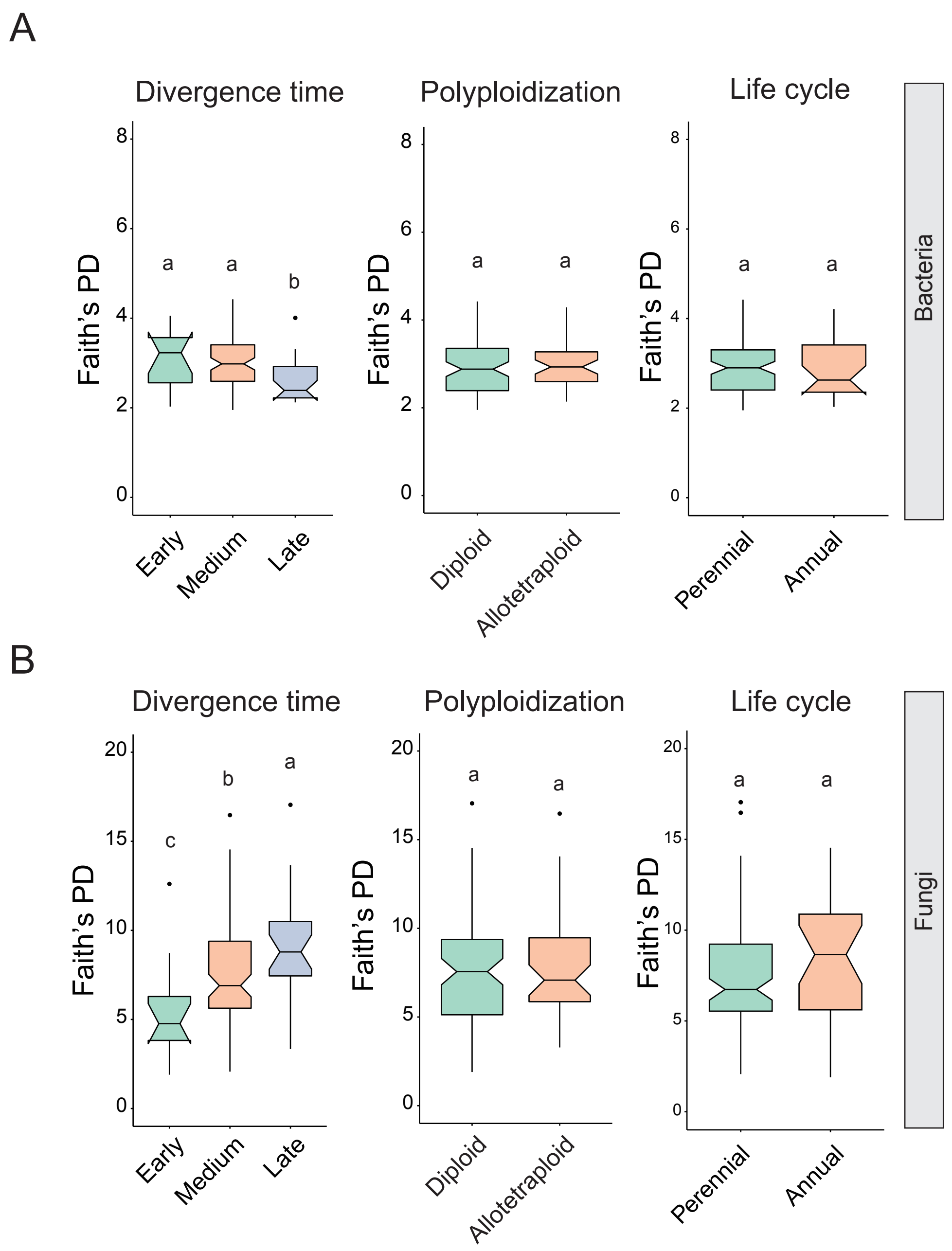

Figure S10 Faith's PD index of root bacterial and fungal community across different host factors.

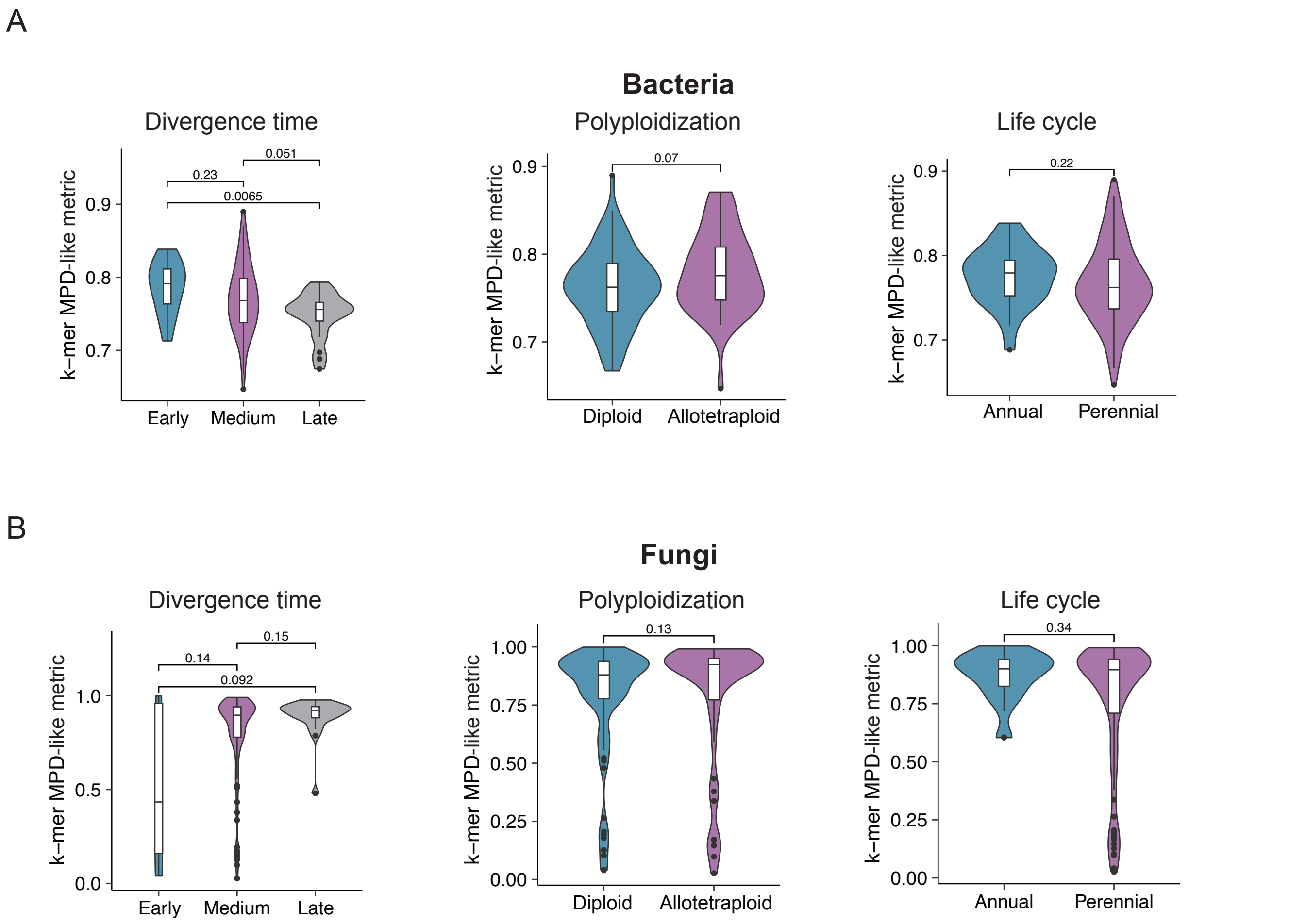

Figure S11. The k-mer MPD-like metric of root bacterial and fungal communities derived from k-mer alpha-diversity estimates.

(A) k-mer MPD-like metric of root bacterial communities, colored by divergence time, polyploidization types, and life cycle types.
(B) k-mer MPD-like metric of root fungal communities, colored by divergence time, polyploidization types, and life cycle types.
k-mers were generated using an n-gram size of k = 16.

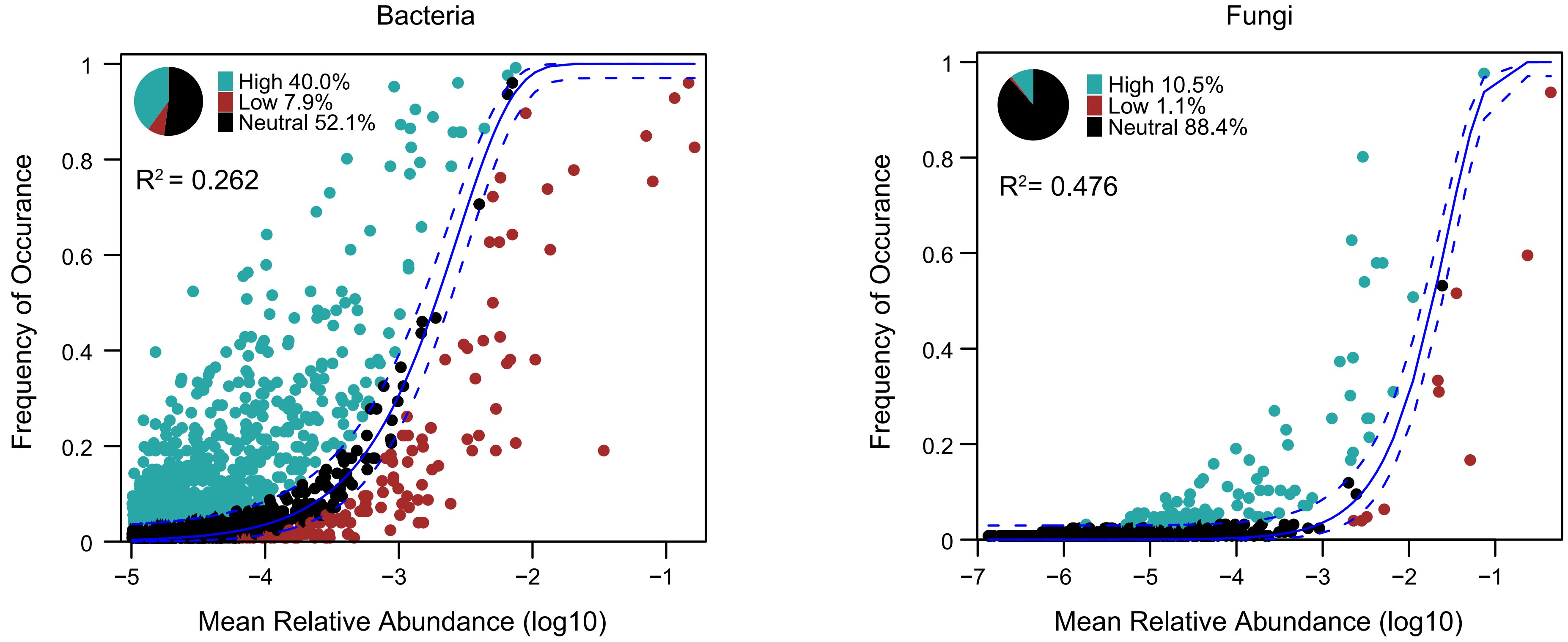

Figure S12 Deterministic and stochastic processes in root microbiome assembly using neutral community model (NCM) analysis.
The fit of the NCM for community assembly. The predicted occurrence frequencies for bacterial and fungal communities. The solid blue lines indicate the best fit to the NCM, and the dashed blue lines represent 95% confidence intervals around the model prediction. ASVs that occur more or less frequently than predicted by the NCM are shown in different colors. R^2 indicates the fit to this model.

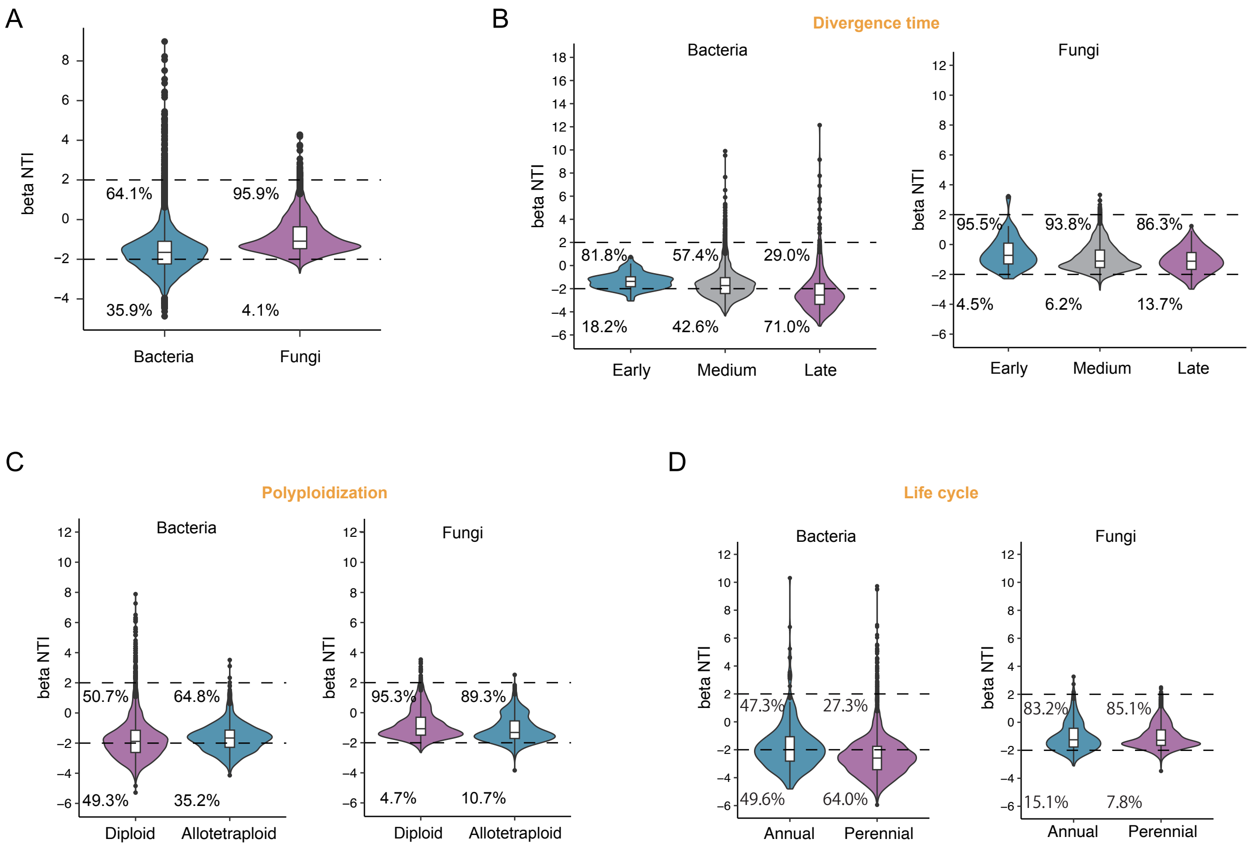

Figure S13 Deterministic and stochastic processes in root microbiome assembly using null model analysis .

(A) The relative contribution of determinism and stochasticity to microbiome assembly based on the βNTI values. The percentages above and below the violin plot represented the proportion of stochastic and deterministic processes in microbiome assembly, respectively.

(B) The relative contribution of determinism and stochasticity on bacteria and fungi assembly among Oryza evolutionary phases based on the βNTI value.

(C) The relative contribution of determinism and stochasticity on bacteria and fungi assembly between diploid and allotetraploid based on the βNTI value.

(D) The relative contribution of determinism and stochasticity on bacteria and fungi assembly between annual and perennial based on the βNTI value.

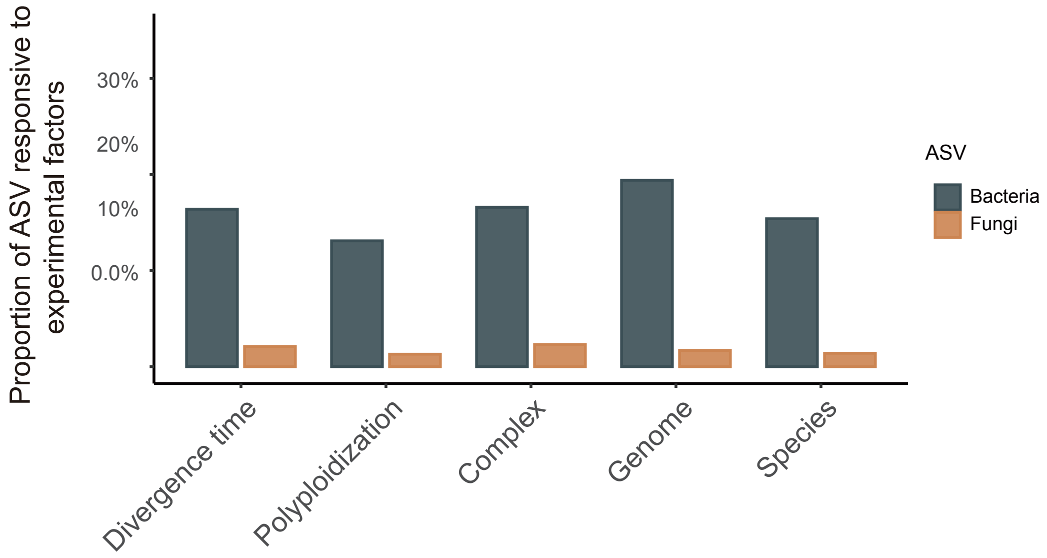

Fig. S14. The proportion of microbial ASVs responsive to host factors exhibited differential abundance.

The proportion of bacterial and fungal ASVs responsive to host factors was shown ($P < 0.05$, GLM). Negative binomial generalized linear models tested whether individual ASV exhibited different abundance across experimental factors (divergence time, polyploidization, complex, genome, and species).

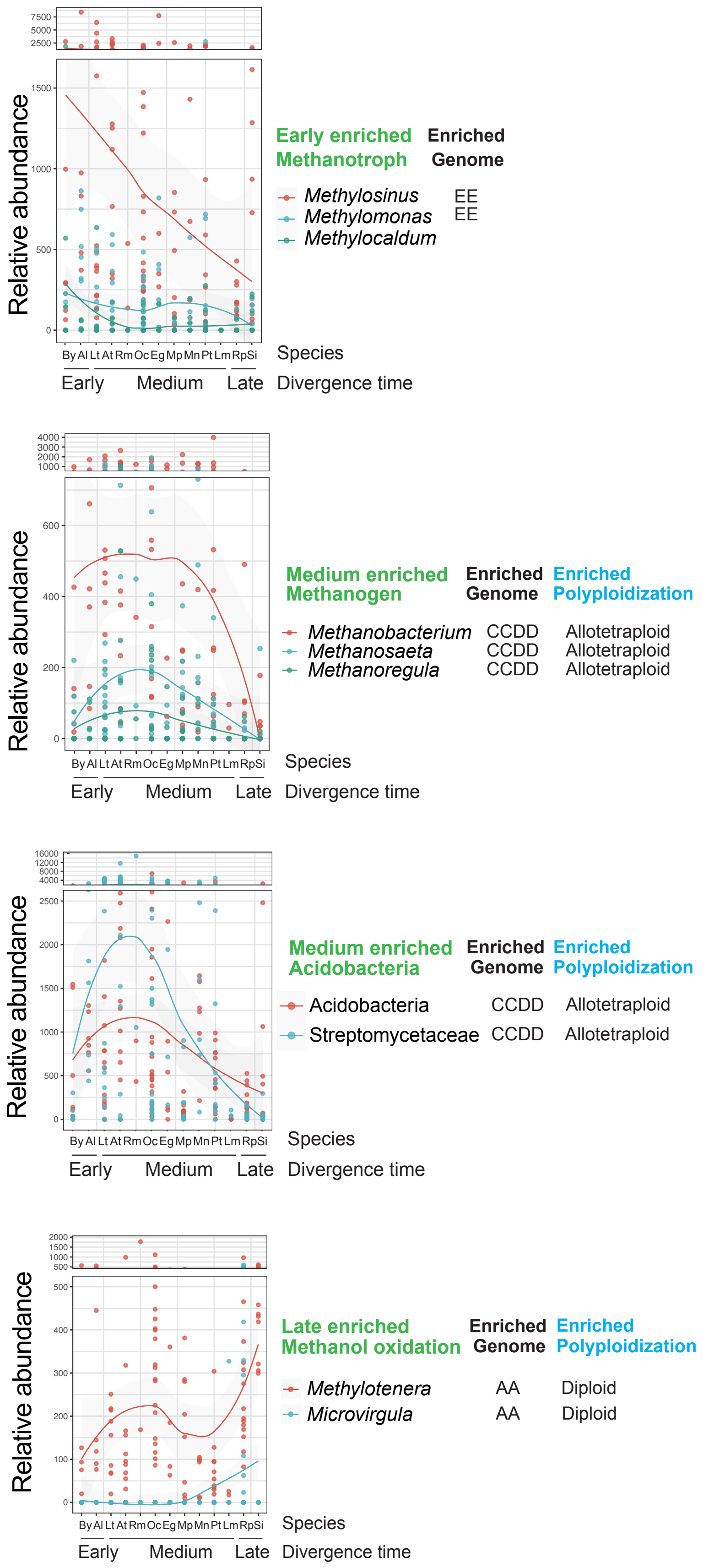

Figure S15 The relative abundance of the potential different bacterial and archaeal function taxa across Oryza species among the different Oryza evolutionary phases based on LEfSe analysis.

Their potential functions were inferred using three complementary approaches: phylogenetic placement, prior literature, and FAPROTAX predictions, rather than by direct experimental verification.
Species abbreviations: By (O. brachyantha), Al (O. australiensis), Lt (O. latifolia), At (O. alta), Rm (O. rhizomatis), Oc (O. officinalis), Eg (O. eichingeri), Mp (O. malampuzhaensis), Mn (O. minuta), Pt (O. punctata), Lm (O. longistaminata), Rp (O. rufipogon), Si (O. sativa indica).

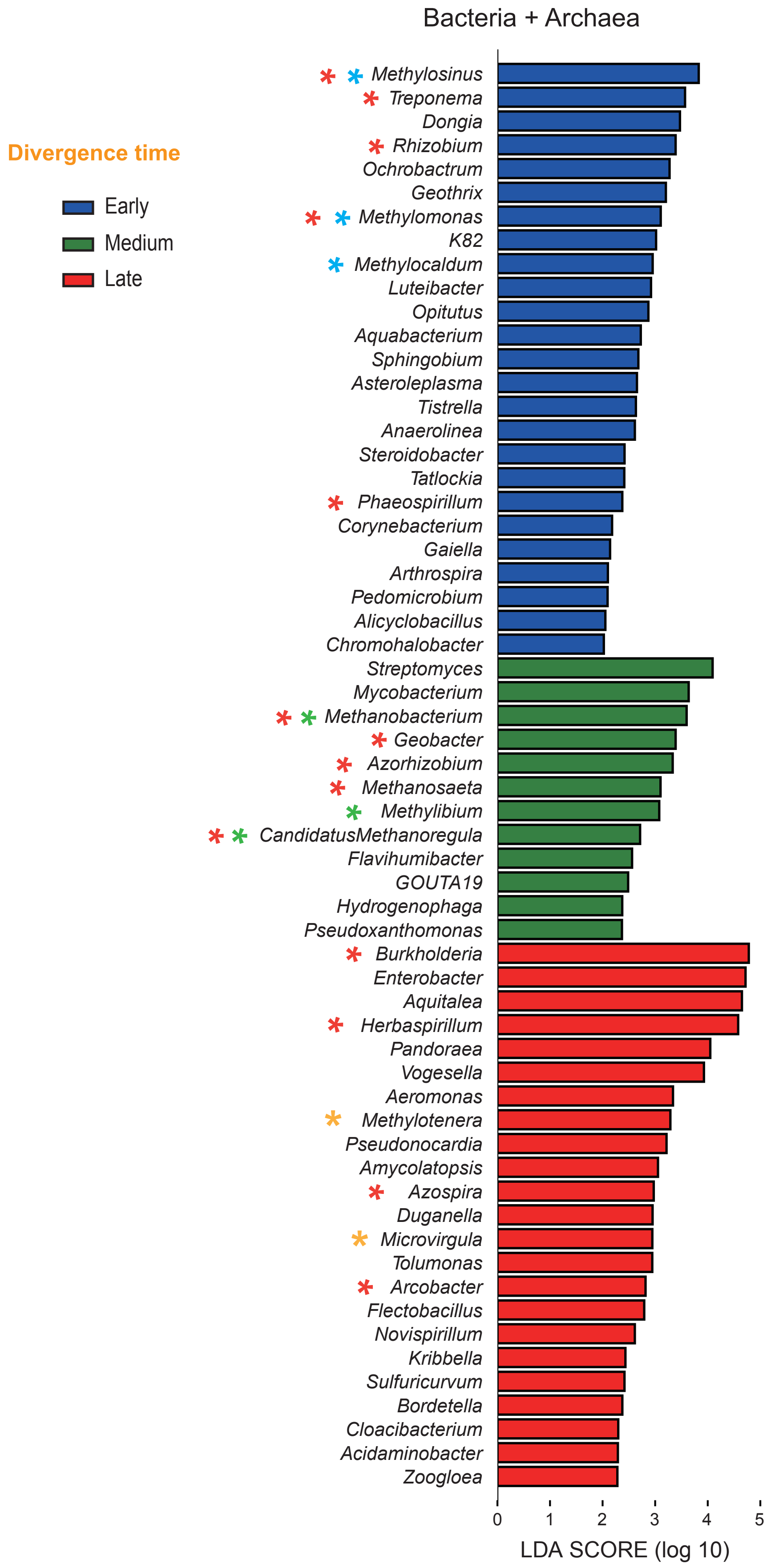

Fig. S16. LEfSe analysis revealed significant bacterial and archaeal genera differences among *Oryza* evolutionary phase.

The genera belonging to nitrogen-fixing taxa were marked by red asterisks. The genera belonging to methanogen were marked by green asterisks. The genera belonging to methanol oxidation were marked by orange asterisks.

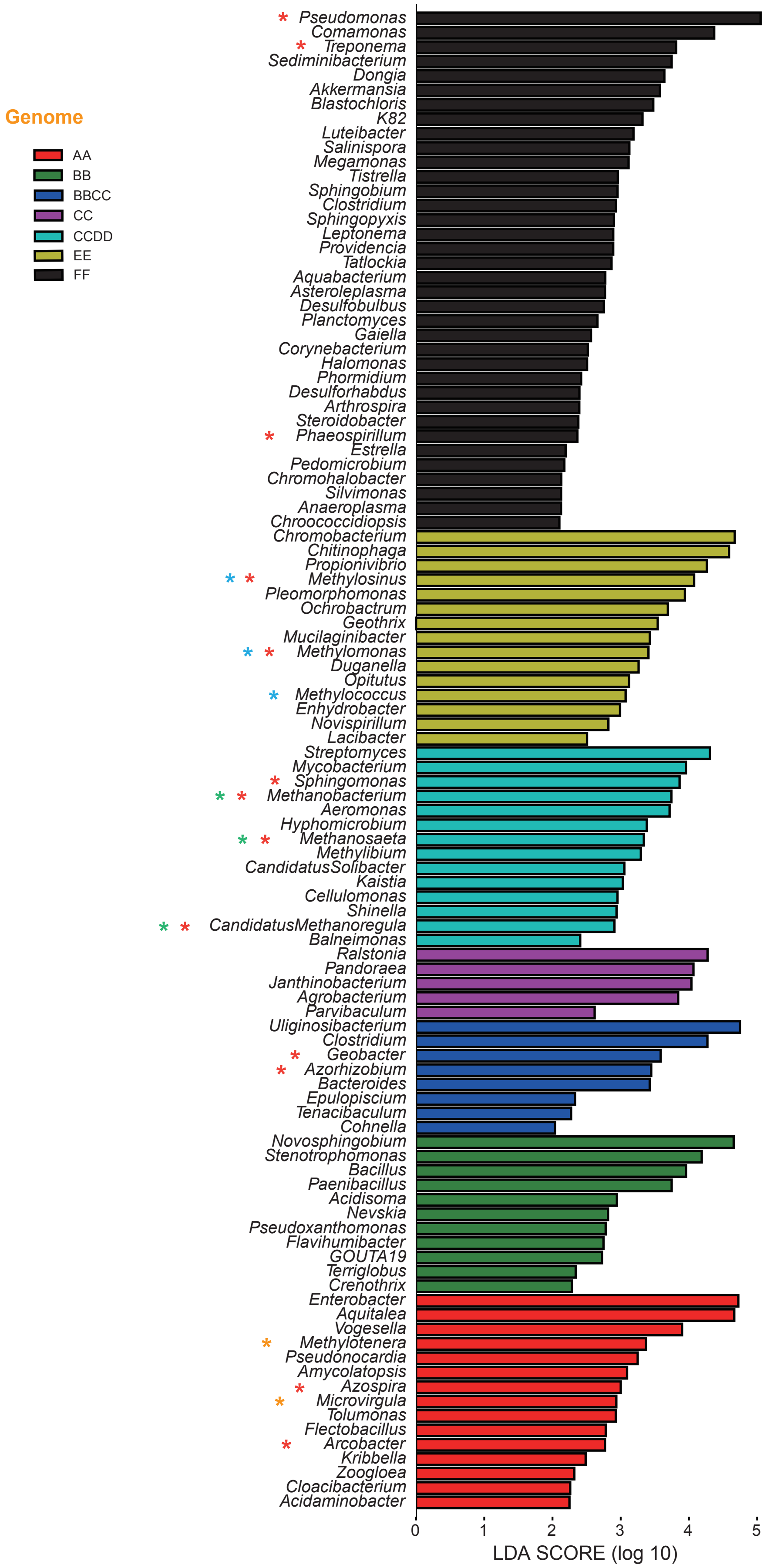

Fig. S17. LEfSe analysis revealed significant bacterial and archaeal genera differences among *Oryza* genome types at the genus level. The genera belonging to methanotrophs were marked by blue asterisks. The genera belonging to nitrogen-fixing taxa were marked by red asterisks. The genera belonging to methanogen were marked by green asterisks. The genera belonging to methanol oxidation were marked by orange asterisks.

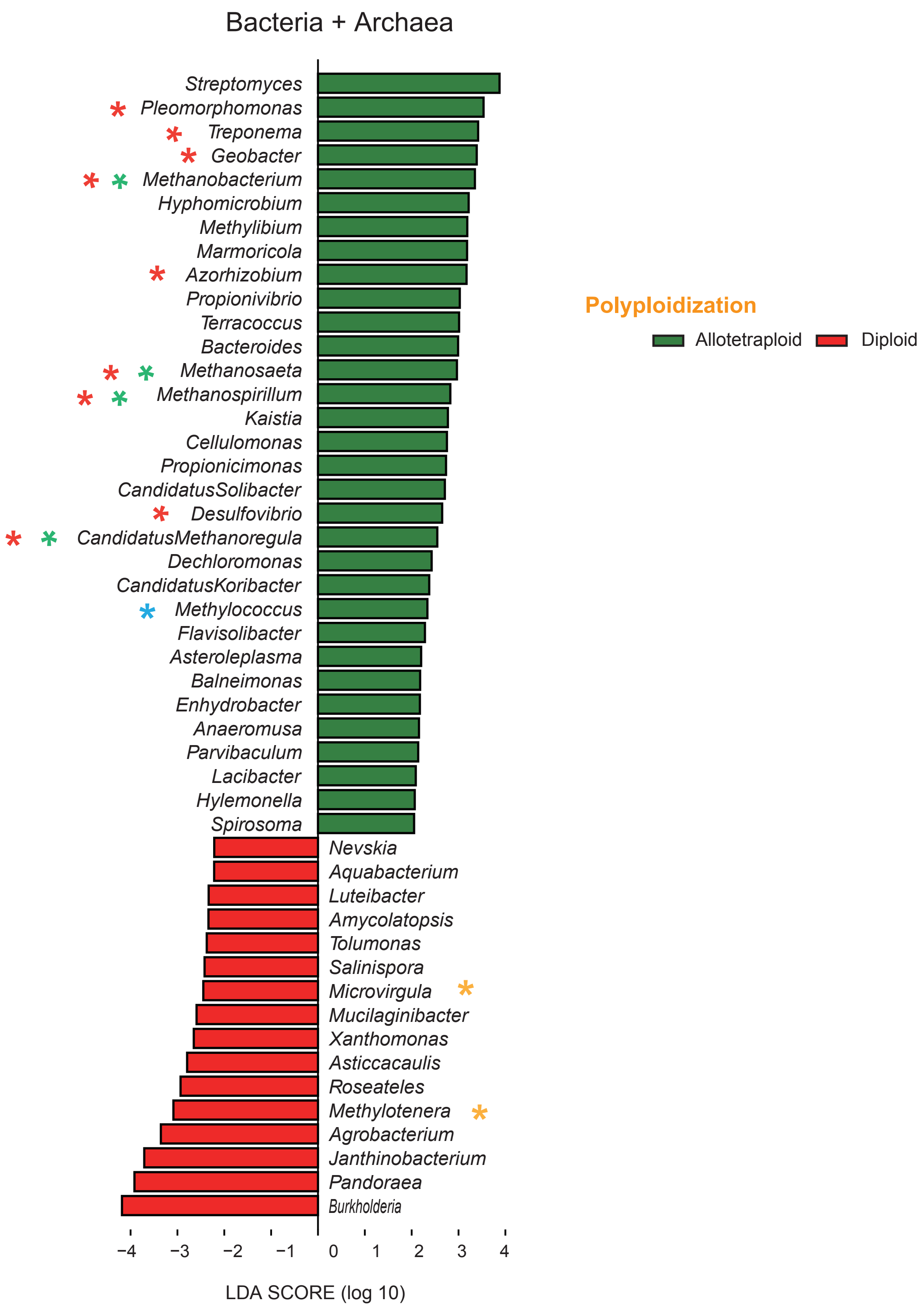

Fig. S18. LEfSe analysis revealed significant bacterial and archaeal genera differences between diploid and allotetraploid.
The genera belonging to methanotrophs were marked by blue asterisks. The genera belonging to nitrogen-fixing taxa were marked by red asterisks. The genera belonging to methanogen were marked by green asterisks. The genera belonging to methanol oxidation were marked by orange asterisks.

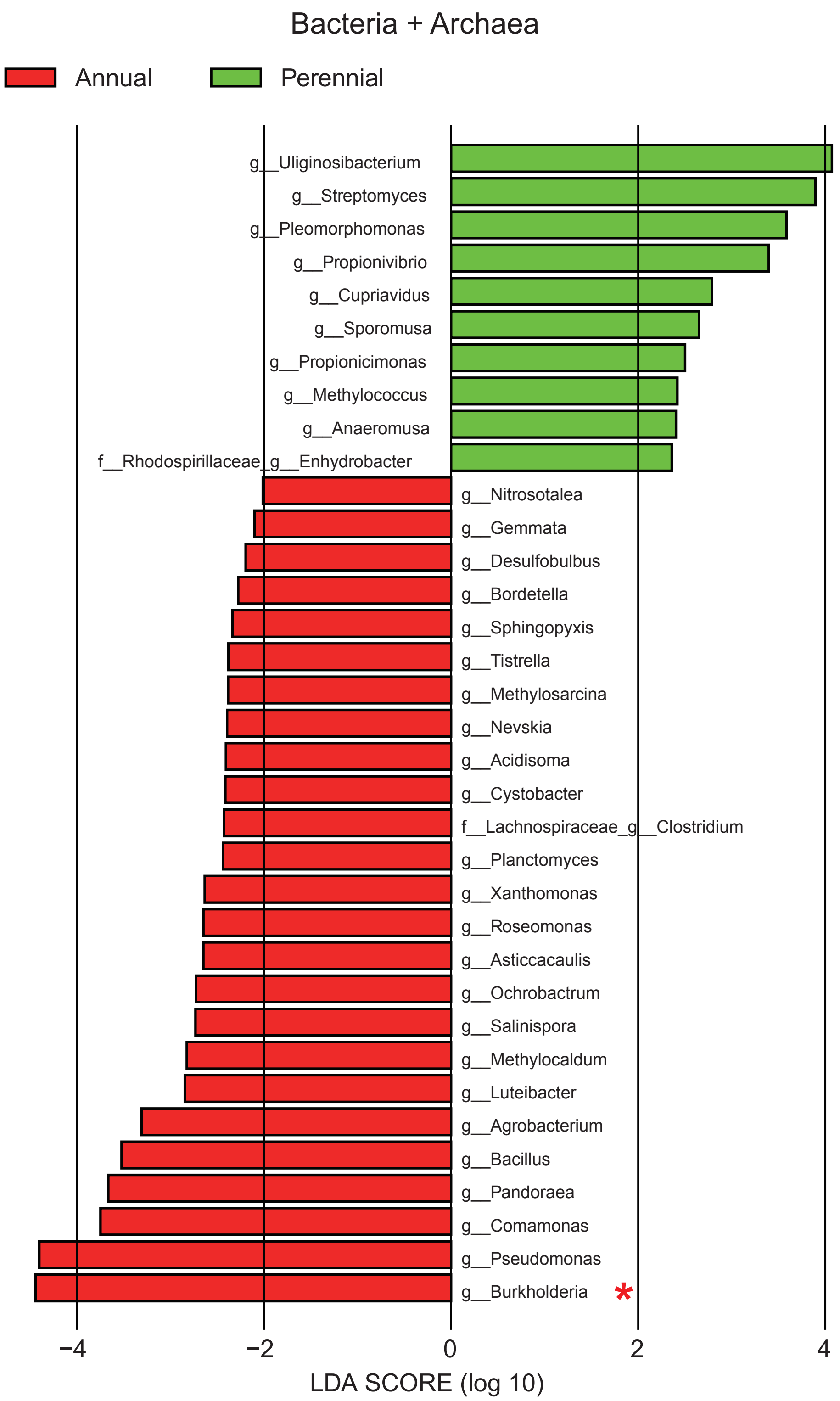

Fig. S19. LEfSe analysis revealed significant bacterial and archaeal genera differences between life cycle.

The genera belonging to nitrogen-fixing taxa were marked by red asterisks.

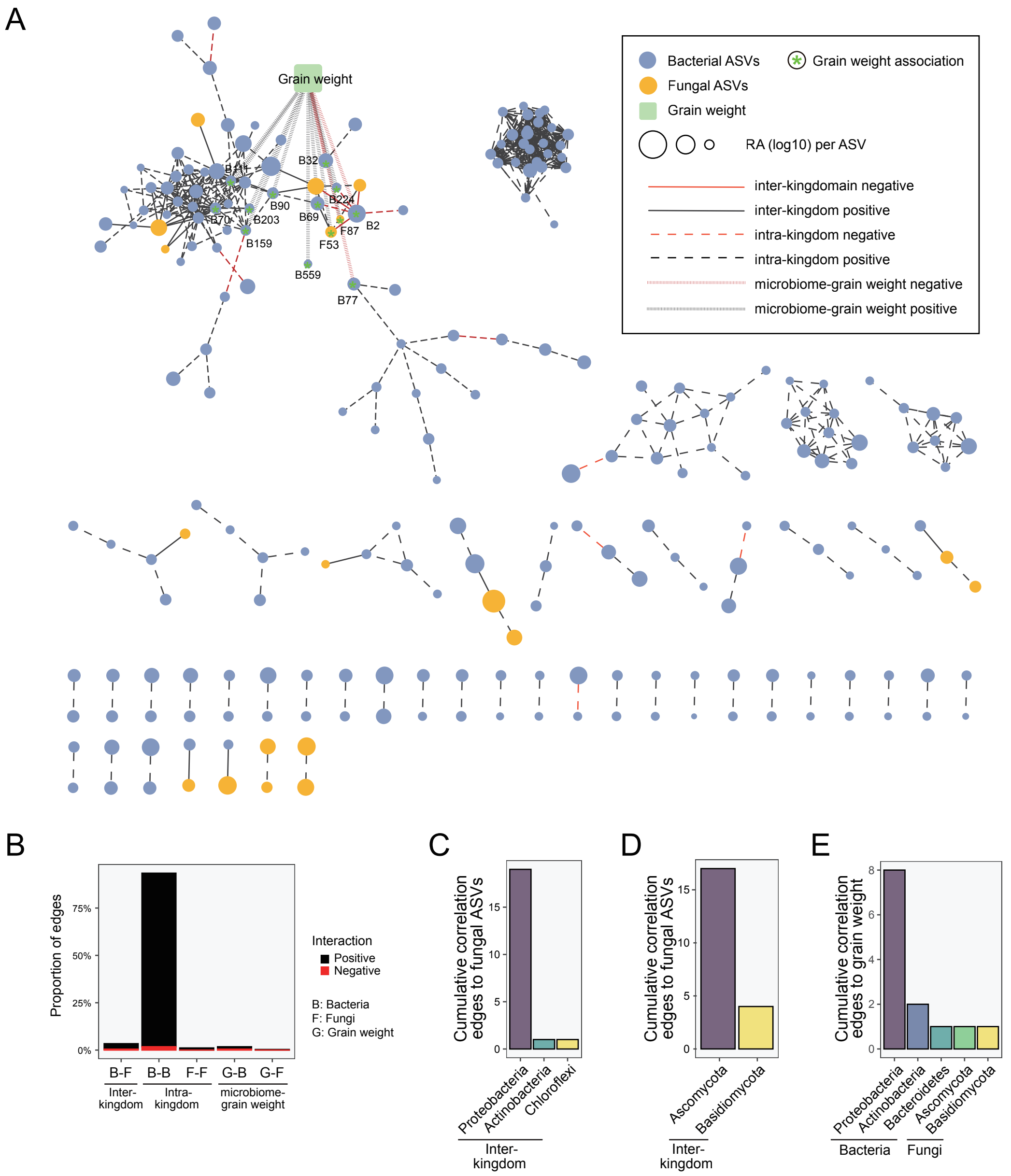

Fig. S20 Root microbial cross-kingdom network associated with grain weight.
(A) SparCC correlation-based network of root microbial ASVs detected in the endosphere across the Oryza species. Nodes were color-coded by microbial type and host trait (grain weight). ASVs belonging to different microbial kingdoms had distinct color codes, and node size reflects their relative abundance (RA) (log10-transformed) in the root endosphere. ASVs associated with grain weight were marked by green asterisks. Edges between nodes correspond to either positive (black) or negative (red) correlations inferred from abundance profiles using the SparCC method (pseudo $p < 0.05$, correlation values > 0.4). Intra-kingdom correlations were represented with dash lines and interkingdom correlations by solid lines. Microbiota and grain weight correlations were represented with vertical slash lines.
(B) The proportion of edges showing positive
(black) or negative (red) correlations in the network. B, bacteria; F, fungi; G, grain weight.
(C) Cumulative correlation edge number in the network between bacterial and fungal ASVs. Bacterial were grouped at the phylum level according to their cumulative correlation edge number with fungal ASVs.
(D) Cumulative correlation edge number in the network between bacterial and fungal ASVs. Fungal were grouped at the phylum level according to their cumulative correlation edge number with bacterial ASVs.
(E) Cumulative correlation edge number in the network between grain weight and microbial ASVs. Bacterial and fungal were grouped at the phylum level according to their cumulative correlation edge number with grain weight.

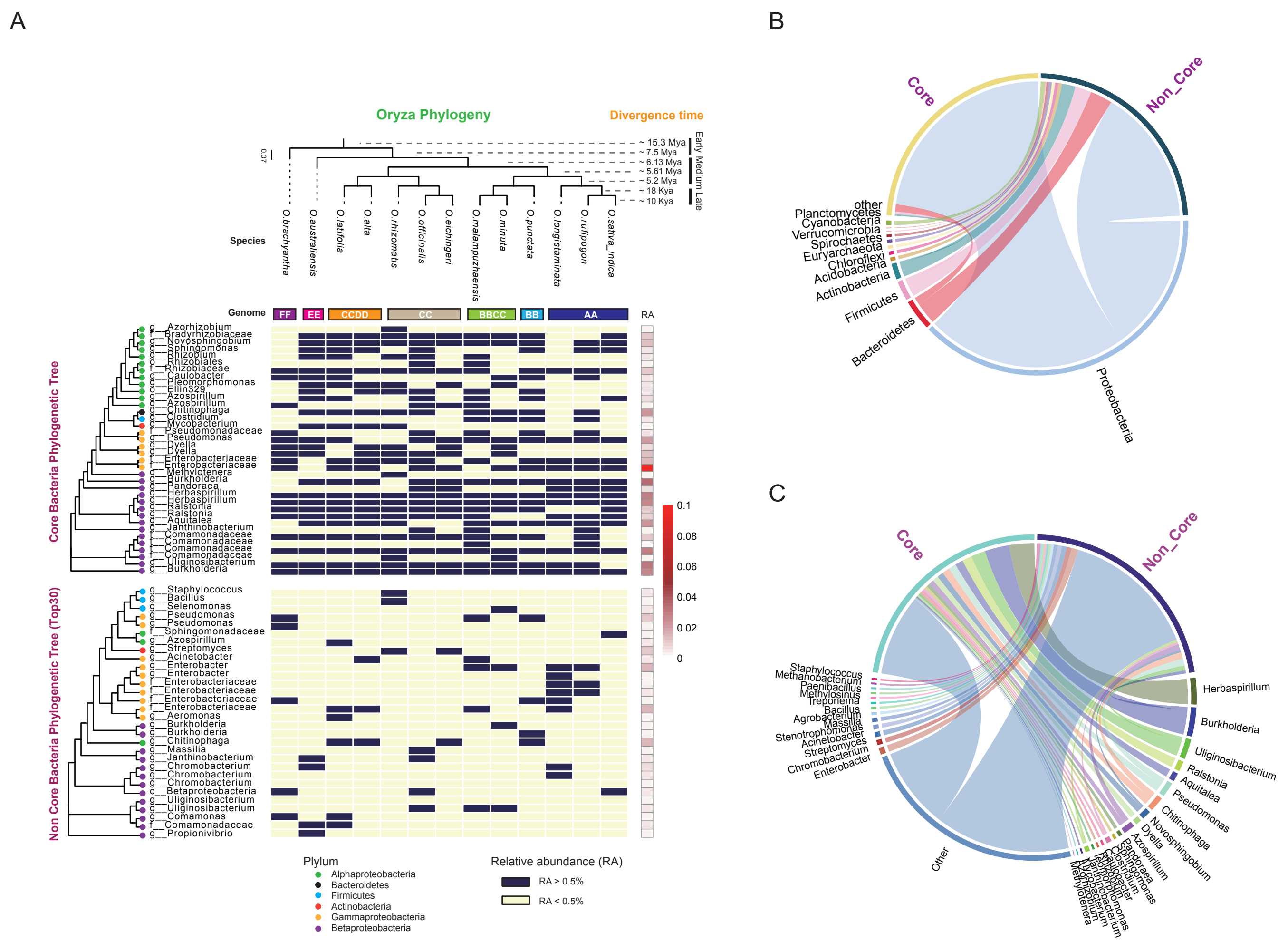

Fig. S21 The taxonomic distribution of root core and non-core bacterial taxa.
(A) The taxonomic distribution of root core and non-core bacterial taxa at different genome type of *Oryza* species.
The taxonomic distribution of root core and non-core bacterial taxa at the phylum (B) and genus (C) level.

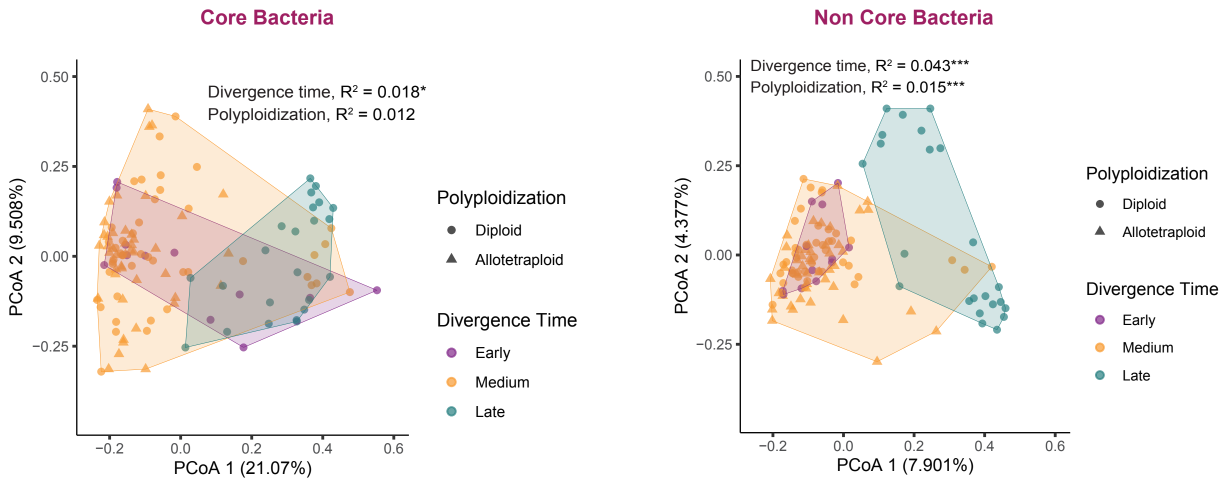

Fig. S22 PCoAs of root core and non-core bacterial communities based on the Bray-Curtis distance matrix.
Permanova showed significant differences in community composition
(*p < 0.05, **p < 0.01, ***p < 0.001).

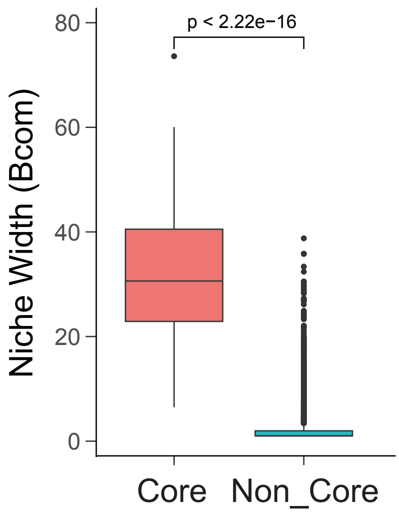

Fig. S23 Mean habitat niche breadths (Bcom) of core and non-core bacterial communities.

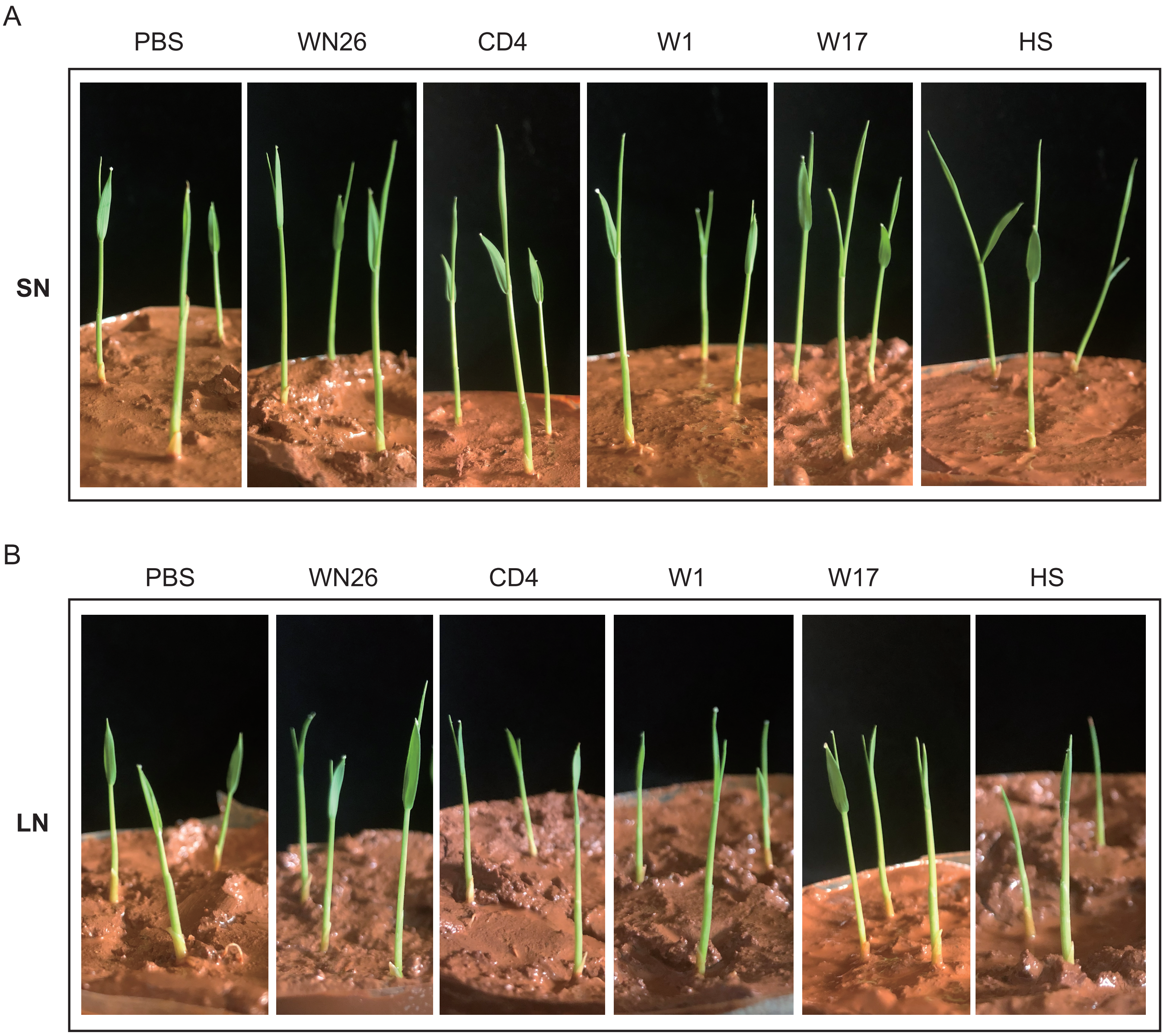

Figure S24. In situ growth of rice seedlings in the soil-autoclaved pathogenicity assay.

(A) Seedlings grown in standard-nitrogen (SN) soil for six days.
(B) Seedlings grown in low-nitrogen (LN) soil for six days.
In both panels, from left to right: Uninoculated control (PBS-treated), and seedlings inoculated with Pseudomonas sp. WN26, Burkholderia sp. CD4, Burkholderia sp. W1, Rhizobium sp. W17, and Herbaspirillum sp. HS. Strains W17 and HS served as non-pathogenic controls.
The soil was autoclaved prior to planting. All seedlings exhibited healthy above-ground morphology with no signs of disease after 6 days of growth in the greenhouse.

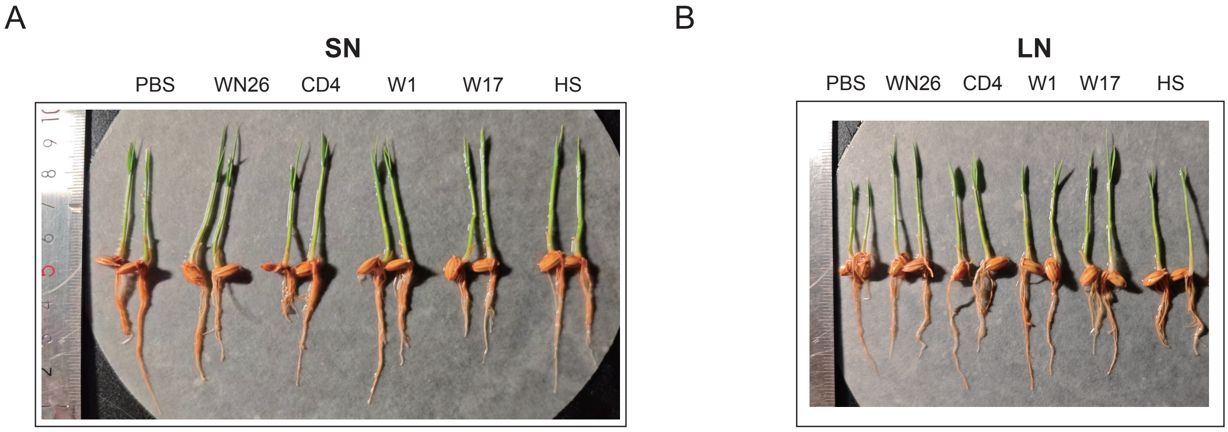

Figure S25. Root systems of rice seedlings from the soil-autoclaved pathogenicity assay after gentle rinsing.

(A) Seedlings grown under standard-nitrogen (SN) conditions for six days.
(B) Seedlings grown under low-nitrogen (LN) conditions for six days.
In both panels, from left to right: Uninoculated control (PBS-treated), and seedlings inoculated with Pseudomonas sp. WN26, Burkholderia sp. CD4, Burkholderia sp. W1, Rhizobium sp. W17, and Herbaspirillum sp. HS. Strains W17 and HS served as non-pathogenic controls. Soil was autoclaved prior to planting.
All seedlings exhibited healthy above-ground morphology with no signs of disease after 6 days of growth in the greenhouse. All seedlings exhibited healthy above-ground morphology with no signs of disease after six days of growth in the greenhouse. Side-by-side comparison shows that all root systems were healthy and intact, with no observable morphological differences or disease symptoms (e.g., necrosis, discoloration) between inoculated seedlings and uninoculated controls. This uniform, healthy phenotype across treatments indicates that none of the tested bacterial strains caused pathogenic effects under these controlled conditions.
